# Syntheses, homeomorphic and configurational isomerizations, and structures of macrocyclic aliphatic dibridgehead diphosphines; molecules that turn themselves inside out†

**DOI:** 10.1039/d2sc04724a

**Published:** 2022-10-20

**Authors:** Yun Zhu, Michael Stollenz, Samuel R. Zarcone, Sugam Kharel, Hemant Joshi, Nattamai Bhuvanesh, Joseph H. Reibenspies, John A. Gladysz

**Affiliations:** Department of Chemistry, Texas A&M University PO Box 30012 College Station Texas 77842-3012 USA gladysz@mail.chem.tamu.edu

## Abstract

The diphosphine complexes *cis*- or *trans*- <svg xmlns="http://www.w3.org/2000/svg" version="1.0" width="19.500000pt" height="16.000000pt" viewBox="0 0 19.500000 16.000000" preserveAspectRatio="xMidYMid meet"><metadata> Created by potrace 1.16, written by Peter Selinger 2001-2019 </metadata><g transform="translate(1.000000,15.000000) scale(0.014583,-0.014583)" fill="currentColor" stroke="none"><path d="M0 480 l0 -400 40 0 40 0 0 360 0 360 480 0 480 0 0 40 0 40 -520 0 -520 0 0 -400z"/></g></svg> PtCl_2_(P((CH_2_)_*n*_)_3_P <svg xmlns="http://www.w3.org/2000/svg" version="1.0" width="19.500000pt" height="16.000000pt" viewBox="0 0 19.500000 16.000000" preserveAspectRatio="xMidYMid meet"><metadata> Created by potrace 1.16, written by Peter Selinger 2001-2019 </metadata><g transform="translate(1.000000,15.000000) scale(0.014583,-0.014583)" fill="currentColor" stroke="none"><path d="M160 840 l0 -40 480 0 480 0 0 -360 0 -360 40 0 40 0 0 400 0 400 -520 0 -520 0 0 -40z"/></g></svg> ) (*n* = b/12, c/14, d/16, e/18) are demetalated by MC≡X nucleophiles to give the title compounds (P((CH_2_)_*n*_)_3_)P (3b–e, 91–71%). These “empty cages” react with PdCl_2_ or PtCl_2_ sources to afford *trans*-MCl_2_(P((CH_2_)_*n*_)_3_P). Low temperature ^31^P NMR spectra of 3b and c show two rapidly equilibrating species (3b, 86 : 14; 3c, 97 : 3), assigned based upon computational data to *in*,*in* (major) and *out*,*out* isomers. These interconvert by homeomorphic isomerizations, akin to turning articles of clothing inside out (3b/c: Δ*H*^‡^ 7.3/8.2 kcal mol^−1^, Δ*S*^‡^ −19.4/−11.8 eu, minor to major). At 150 °C, 3b, c, e epimerize to (60–51) : (40–49) mixtures of (*in*,*in*/*out*,*out*) : *in*,*out* isomers, which are separated *via* the bis(borane) adducts 3b, c, e·2BH_3_. The configurational stabilities of *in*,*out*-3b, c, e preclude phosphorus inversion in the interconversion of *in*,*in* and *out*,*out* isomers. Low temperature ^31^P NMR spectra of *in*,*out*-3b, c reveal degenerate *in*,*out*/*out*,*in* homeomorphic isomerizations (Δ*G*^‡^_Tc_ 12.1, 8.5 kcal mol^−1^). When (*in*,*in*/*out*,*out*)-3b, c, e are crystallized, *out*,*out* isomers are obtained, despite the preference for *in*,*in* isomers in solution. The lattice structures are analyzed, and the *D*_3_ symmetry of *out*,*out*-3c enables a particularly favorable packing motif. Similarly, (*in*,*in*/*out*,*out*)-3c, e·2BH_3_ crystallize in *out*,*out* conformations, the former with a cycloalkane solvent guest inside.

## Abstract 2

It’s not a magic trick. Molecules can turn themselves inside out, just like articles of clothing or other familiar household objects. This behavior is demonstrated for the title compounds through a combination of synthesis, rate, and NMR studies.

## Graphical abstract

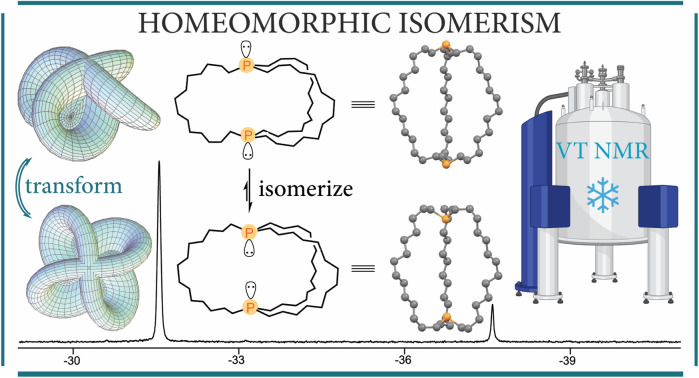

## Introduction

**Scheme 1:**
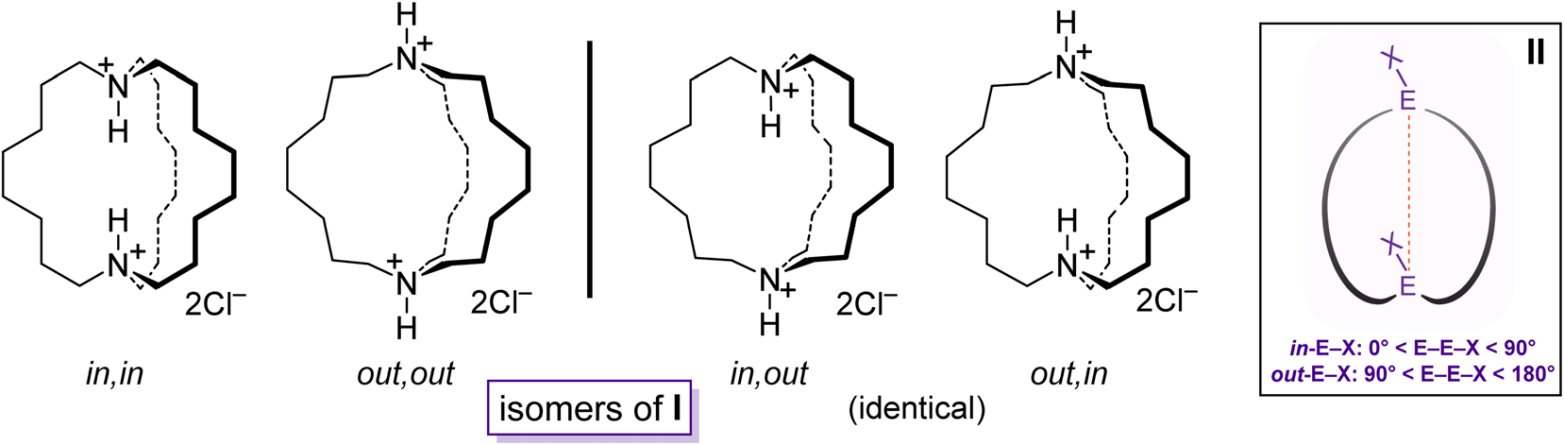
Dibridgehead diammonium salts that illustrate the limiting types of *in*/*out* isomers (I), and geometric criteria for *in*/*out* bridgeheads EX (II).

Bicyclic compounds with two bridgehead heteroatoms are quite common for nitrogen but less familiar for other elements. The many dinitrogen examples include DABCO (1,4-diazobicyclo[2.2.2]octane), cryptands,^1^ and the macrocyclic diprotonated diamines I (Scheme 1).^2^ The last group represents touchstones for many of the phenomena detailed below.^2^ In contrast, the corresponding aliphatic dibridgehead diphosphines, or Brønsted or Lewis acid adducts thereof, are much less explored. Prior to the work herein, compounds of the formula P((CH_2_)_*n*_)_3_P were unknown for *n* > 4.^3^

Macrocyclic versions of such molecules, as well as analogs with carbon bridgeheads, can exist as *in*/*out* isomers^4^ differing in the relative orientations of the bridgehead substituents. As shown for I (Scheme 1), four limits apply: *in*,*in*, *out*,*out*, *in*,*out*, and *out*,*in*. When the northern and southern hemispheres are identical, the last two are degenerate. For structures that lack a *C*_3_ axis, *in*/*out* geometries can be assigned from the angles defined by the two bridgehead atoms and their substituents, as shown in II. For some time, *in*/*out* isomerism has been a curiosity, but the phenomenon has now been coupled to function, for example in the selective transport of metal dichloride fragments.^5^

**Scheme 2:**
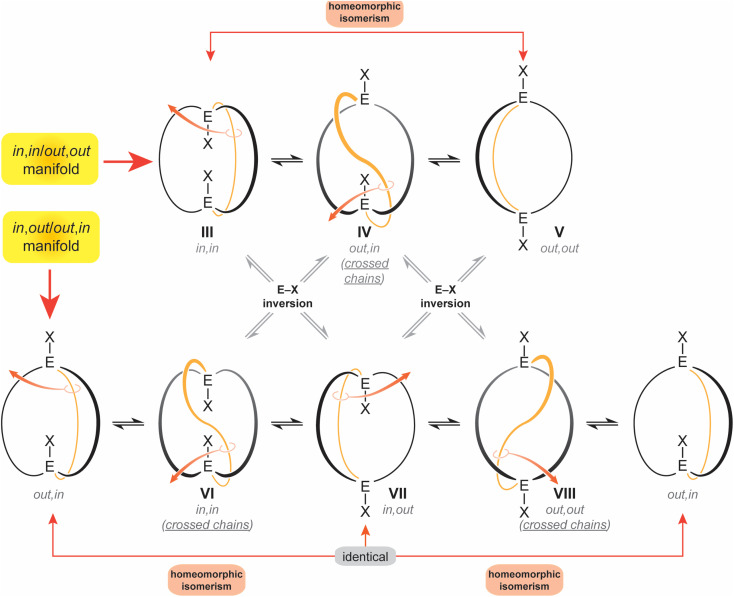
Conventional *in*,*in* (III), *out*,*out* (V), and *in*,*out* (VIII) isomers of a bicyclo[*z*.*z*.*z*] system with EX bridgeheads, and “crossed chain” variants IV, VII and VIII.

The diprotonated dibridgehead diamines I represent the historical “ground zero” for the study of homeomorphic isomerization.^4^ This terminology, imported from the field of topology,^6^ denotes a dynamic process that is tantamount to turning a molecule inside out. Essentially, one of the tethers connecting the bridgehead atoms is “pulled through” the macrocycle defined by the other two, like reaching inside a rubber glove and pulling the inner surface outside. As depicted in Scheme 2 as well as a video (ESI†), this interconverts *in*,*in* and *out*,*out* isomers, and *in*,*out* and *out*,*in* isomers – in both cases, an apparent inversion of configuration at each bridgehead atom.^7^

Interestingly, data for the *in*,*in*, *out*,*out* and *in*,*out* equilibria of the diprotonated dibridgehead diamines I (Scheme 1) implicated an alternative mechanism: namely, proton dissociation, pyramidal inversion of the resulting trivalent nitrogen atom, and nitrogen reprotonation.^2^ Indeed, when the bridgeheads are trivalent group 15 heteroatoms (E:), simple pyramidal inversion can effect *in*/*out* isomerization, as represented by the “×” pathways connecting the upper and lower manifolds in Scheme 2. However, in the absence of unprecedented cooperative phenomena, only *one inversion at a time* would be expected, and given well established trends in inversion barriers,^8,9^ this would be rapid at room temperature only in the case of nitrogen.

For the sake of completeness, three species with “crossed chains” (IV, VI, VIII) are also depicted in Scheme 2. The conformational details of these isomerizations remain beyond the scope of this study, but such architectures receive support in dynamic simulation computations, which locate abundant numbers of local minima.^10^ As exemplified in the Discussion section, isomers of the type VIII have been isolated for P–X and Si–X systems with sterically demanding X groups.^11–13^ In all of these contexts, the “inside out” nature of homeomorphic isomerization means that functionality might be directed in a convergent manner towards an interior domain in one conformation, and externally in the other.

Apart from this work, only five molecular systems have been definitively shown to undergo homeomorphic isomerization,^4,14,15^ as defined by (1) the ability to observe both *in*,*in* and *out*,*out* forms and establish a direct path between them, or (2) the ability to observe inequivalent bridgehead atoms of an *in*,*out* isomer (the E–X in VII are not related by a symmetry operation), and their exchange at higher temperatures. In other cases, there can be a strong inference of homeomorphic isomerization from NMR properties, but with data for both limits remaining elusive.^12,16,17^ Particularly noteworthy are earlier efforts by Habicher and Bauer that encompass several types of bicyclic dibridgehead diphosphorus compounds with *p*-phenylene linkers in the tethers.^16,17^ They and Alder^4^ were pioneers in articulating some of the concepts expressed above.

**Scheme 3:**
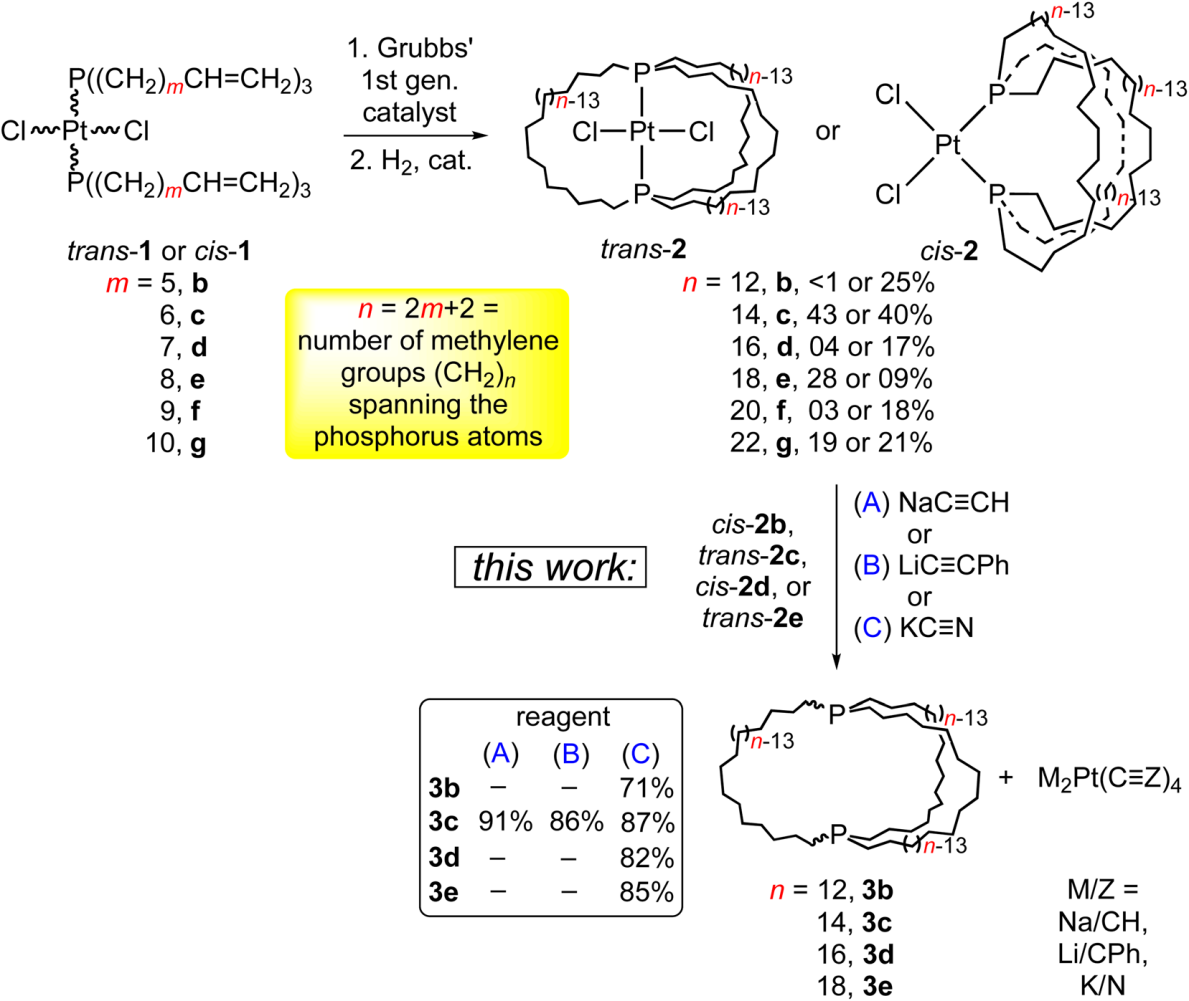
Syntheses of the title compounds (3b–e) from platinum complexes.

Previous synthetic studies from the authors’ laboratory lay the groundwork for the effort detailed herein. As shown in Scheme 3 (top), platinum dichloride complexes with two *trans* or *cis* alkene containing phosphines of the formula P((CH_2_)_*m*_CH=CH_2_)_3_ undergo three fold intramolecular ring closing metathesis when treated with Grubbs’ catalyst.^18,19^ Subsequent hydrogenations give what are termed “gyroscope-like” (*trans*) or “parachute-like” (*cis*) complexes in modest yields. The *trans* isomers are adducts of *in*,*in* dibridgehead diphosphine ligands, and the *cis* isomers adducts of *out*,*out* ligands. This underscores the appreciable conformational flexibility of the ligands.

In this paper, we report (1) the facile liberation of four aliphatic dibridgehead diphosphine ligands P((CH_2_)_*n*_)_3_P (3) from 2, (2) detailed variable temperature NMR analyses of 3, which in some cases allow the observation of distinct *in*,*in* and *out*,*out* isomers, (3) thermal epimerisations that equilibrate the *in*,*in*/*out*,*out* mixtures and *in*,*out* isomers, (4) the formation and deprotection of the corresponding bis(borane) adducts, (5) six crystals structures, and in-depth analyses thereof. Companion computational investigations that support certain assignments also deserve emphasis.^10,20^ Small portions of these data have been communicated, CCDC 838916 (out,out-3c·BH_3_·(C_5_H_9_CH_3_)) and CCDC 838917 (out,out-3c·2BH_3_·(C_6_H_11_CH_3_)).^5,21^

## Results

### Syntheses of title compounds 3

As shown in Scheme 3 (bottom), the platinum dichloride complex *trans*-2c was treated with excesses of NaC≡CH, LiC≡CPh, or KC≡N. Workups gave the dibridgehead diphosphine 3c (*n* = 14) in 86–91% yields. In the reaction with KC≡N, the concurrent formation of K_2_Pt(C≡N)_4_ was verified by ^13^C{^1^H} NMR and X-ray crystallography. In that with LiC≡CPh, the salt Li_2_Pt(C≡CPh)_4_·4THF could be isolated in 35% yield. A sample was independently synthesized from the reaction of LiC≡CPh and PtCl_2_(THT)_2_ (THT = tetrahydrothiophene). Similar reactions of *cis*-2b, d and *trans*-2e with KC≡N gave the diphosphines 3b, d, e in 71–85% yields. These feature 26- to 38-membered macrocycles.

Compounds 3b–e were isolated as low melting white solids that were moderately air sensitive, especially in solution.^22^ They were characterized by NMR spectroscopy (^1^H, ^13^C{^1^H}, ^31^P{^1^H}) and other techniques as described below. Data are summarized in the Experimental section. The three P*C̲*H_2_*C̲*H_2_*C̲*H_2_^13^C{^1^H} NMR signals showed comparable ^*n*^*J*_CP_ values (10–12 Hz), but more detailed assignments could not be made with certainty. Several *cis*-PtCl_2_(diphosphine) species similarly react with excess KC≡N to give K_2_Pt(C≡N)_4_.^23^

### Probes of stereochemistry and dynamic behavior

**Scheme 4:**
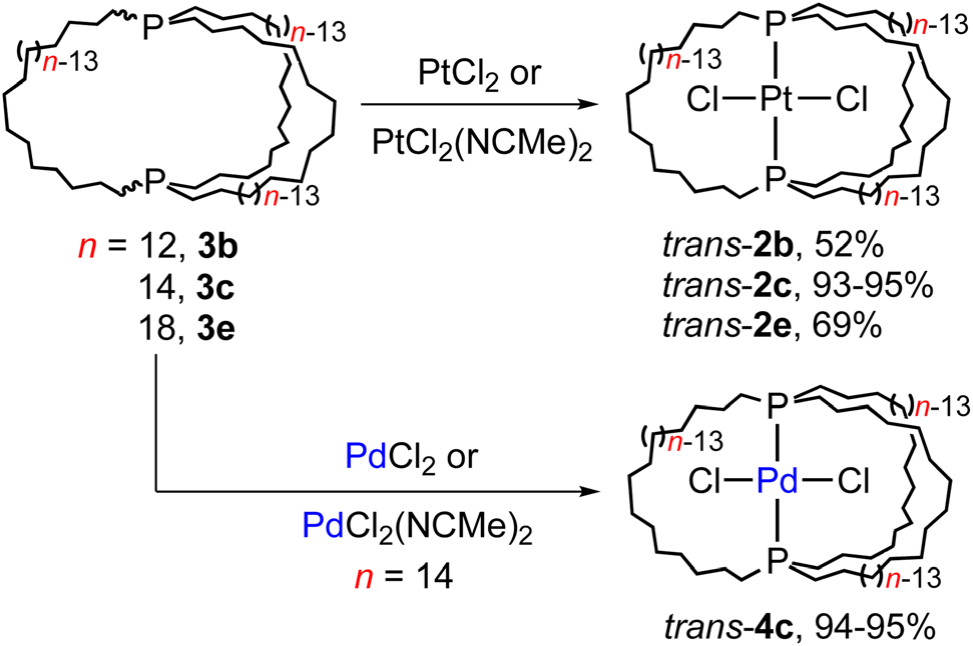
Reactions of title compounds with MCl_2_ sources.

One initial question concerns the distribution of *in*/*out* stereoisomers of 3b–e produced in Scheme 3. The diphosphine ligands in *trans*-2c, e and *cis*-2b, d have *in*,*in* and *out*,*out* orientations, respectively, and the corresponding free ligands can interconvert by homeomorphic isomerizations as summarized in Scheme 2. As shown in Scheme 4, the diphosphines 3b, c, e reacted with PtCl_2_ sources to generate the gyroscope-like platinum complexes *trans*-2b, c, e. Comparable reactions of 3c and PdCl_2_ sources afforded *trans*-4c, which had been previously synthesized by a route analogous to *trans*-2c.^18*a*^ Importantly, *trans*-2b is a new compound, unavailable in significant quantities by the direct route in Scheme 3.

Trialkylphosphines normally exhibit appreciable pyramidal inversion barriers (Δ*G*^‡^_403 K_ = 32–35 kcal mol^−1^),^8^ with temperatures of ≥140 °C typically required to effect racemization or epimerization. Hence, the efficient reconstitution of *trans*-2c, e under mild conditions in Scheme 4 seemingly excludes the formation of *in*,*out*-3b–e in Scheme 3. In separate studies, *trans*-2c–e have been shown to be much more stable than *cis*-2c–e (equilibration at *ca.* 160 °C, with Δ*G* for 2c 3–12 kcal mol^−1^ depending upon the medium and temperature by DFT).^19,24^ Hence, the absence of *cis* products in Scheme 4 is not surprising.

**Fig. 1:**
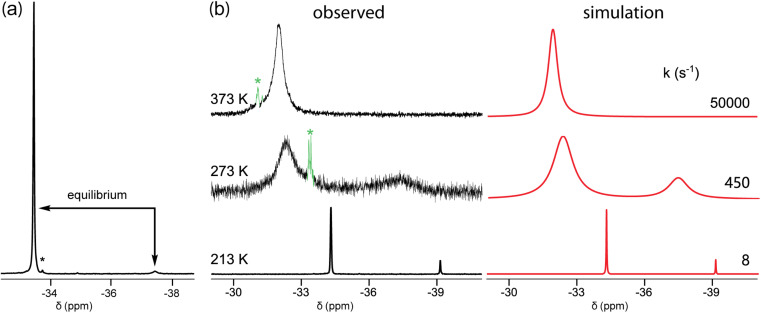
^31^P{^1^H} NMR spectra in toluene-*d*_8_: (a) 3c at 193 K; (b) 3b at 213 K, 273 K, and 373 K, together with simulated spectra. The label * indicates an impurity.

In the course of characterizing the reactivity of 3b–e, low temperature ^31^P{^1^H} NMR spectra were acquired. When toluene or toluene-*d*_8_ solutions of 3c were cooled, a small upfield peak reproducibly appeared as depicted in Fig. 1a and S1 (ESI†). The line widths for the major signal varied strongly (*w*_1/2_ 5.8 to 93.0 Hz, 300 K to 233 K, decreasing to 6.3 Hz at 193 K; Fig. S5, ESI†). A ^31^P EXSY experiment (Fig. S2†) confirmed that the species responsible for the two peaks are in equilibrium. The area ratio at 193 K (−80 °C), 97 : 3, corresponded to a Δ*G*_193K_ value of 1.33 kcal mol^−1^.

The smaller macrocycle 3b also exhibited two signals in toluene-*d*_8_ at lower temperatures (Fig. 1b and S3†). At 213 K (−60 °C), integration indicated a 86 : 14 isomer ratio, for a Δ*G*_213K_ value of 0.77 kcal mol^−1^, with the downfield signal again dominant. These coalesced at approximately 303 K (30 °C), and ^13^C{^1^H} NMR signals also sharpened above this temperature (Fig. S4†). Two ^31^P{^1^H} NMR signals were also observed in mesitylene (85 : 15, 213 K; *T*_c_ near 313 K) and THF (91 : 9, 213 K; *T*_c_ near 263 K).^25^ For both 3b and c, the spectra were simulated, as exemplified for 3b in Fig. 1b and S3.† Eyring plots of the rate constants derived from the line shapes^26^ (Fig. S7†) gave Δ*H*^‡^ and Δ*S*^‡^ values of 7.3 kcal mol^−1^ and −19.4 eu for 3b (minor to major; Δ*G*^‡^_213 K_ = 11.4 kcal mol^−1^ or 12.1 kcal mol^−1^ major to minor), and 8.2 kcal mol^−1^ and −11.8 eu for 3c (minor to major; Δ*G*^‡^_193 K_ = 10.4 kcal mol^−1^, or 11.5 major to minor).^26^

Accordingly, the two ^31^P{^1^H} NMR signals are attributed to *in*,*in* and *out*,*out* isomers of 3b, c that rapidly interconvert by homeomorphic isomerization, and these descriptors are henceforth coupled to their alphanumeric designations. Perhaps counterintuitively, the major species have been assigned (for all macrocycle sizes) as *in*,*in*-3b–e. A strong rationale is provided in accompanying computational papers (which also show the ^31^P{^1^H} NMR signals of *in* P: bridgeheads to be 3.5–8.0 ppm downfield of *out* P: bridgeheads).^10*b*,20,27^ As elaborated below, dispersion forces are thought to play a key role. Analogous trends were found in earlier computational studies of the hydrocarbon bicyclo[6.6.6]eicosane and related species.^29^ However, the *in*,*in* thermodynamic preference is not reflected in crystal structures (*vide infra*), and of course cannot extend to smaller ring systems (*e.g.*, DABCO).

### BH_3_ adducts and thermal phosphorus epimerization

In principle, *in*,*in*-3b–e and *out*,*out*-3b–e could interconvert by sequential pyramidal inversions at each phosphorus atom. Although it was viewed as highly unlikely that such processes played any roles in the preceding phenomena, authentic samples of several of the potential intermediates, *in*,*out*-3b–e, were sought. If they were to be stable with respect to the *in*,*in* and *out*,*out* isomers at room temperature, their intermediacies could be definitively excluded.^30^

**Scheme 5:**
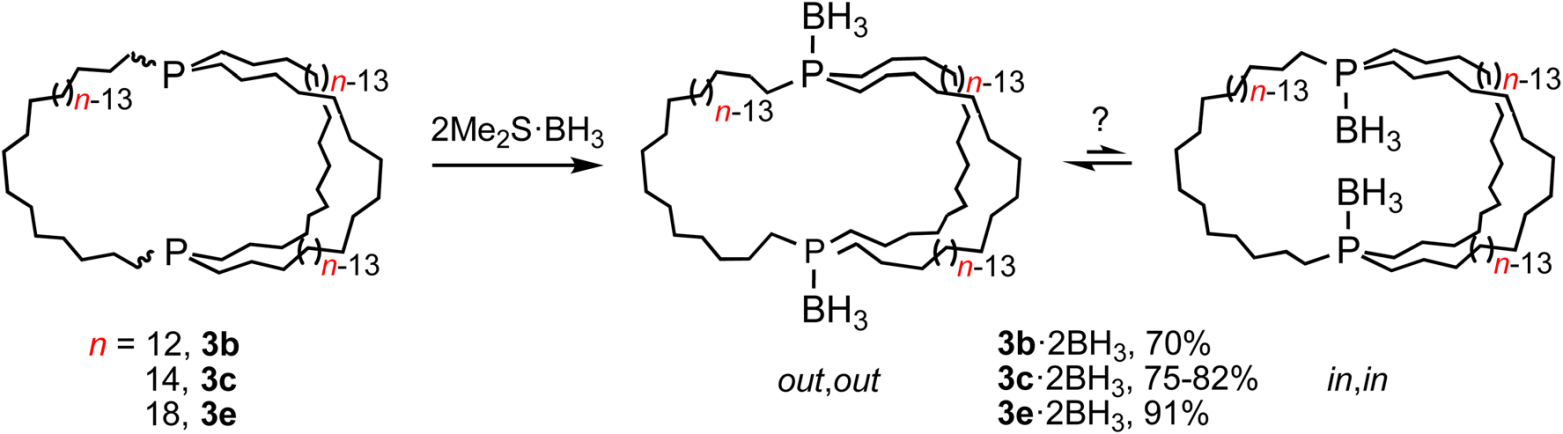
Conversion of title compounds to dibridgehead diphosphine diboranes.

As a prelude, (*in*,*in*/*out*,*out*)-3b, c, e were treated with moderate excesses of Me_2_S·BH_3_. As shown in Scheme 5, workups gave the diphosphine diboranes (*in*,*in*/*out*,*out*)-3b, c, e·2BH_3_ as white or light yellow analytically pure air stable solids or oils in 70–91% yields. These materials, unlike the diphosphines, can be purified chromatographically. They were characterized analogously and exhibited, like other phosphine boranes, broad boron-coupled ^31^P NMR and BH_3_^1^H NMR signals.^31^ Their isomer distributions are also of interest, but this topic is deferred to future papers.

**Scheme 6:**
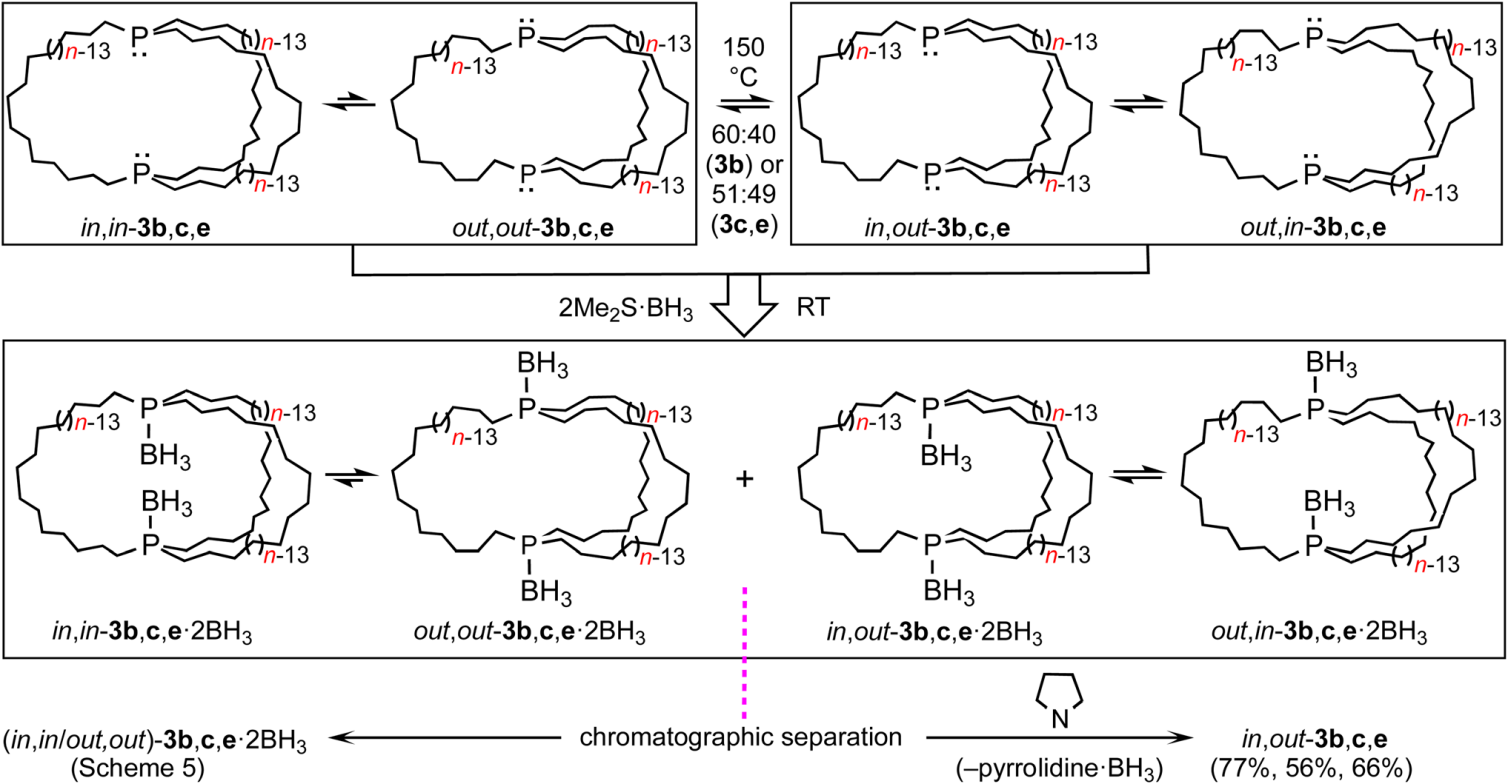
Thermal epimerization of (*in*,*in*/*out*,*out*)-3 to *in*,*out*-3 and separations *via* BH_3_ adducts.

As depicted in Scheme 6, mesitylene solutions of (*in*,*in*/*out*,*out*)-3b, c, e were kept at 150 °C and monitored by ^31^P{^1^H} NMR. As exemplified in Fig. S8,† a new signal gradually appeared in each case. These were assigned to the epimerization products *in*,*out*-3b, c, e. After 30–60 h, 60 : 40 (3b) to 51 : 49 (3c, e) equilibrium mixtures were obtained ((*in*,*in*/*out*,*out*):*in*,*out*). The rate of epimerization of 3c (*k*_1_ = 1.47 × 10^−5^ s^−1^) gave a Δ*G*^‡^_423 K_ value of 34.4 kcal mol^−1^ (Δ*G*_423 K_ = 0.03 kcal mol^−1^), in good agreement with pyramidal inversion barriers of trialkyl monophosphines.^8^

Practical direct separations of (*in*,*in*/*out*,*out*)-3b, c, e and *in*,*out*-3b, c, e could not be devised. Thus, as shown in Scheme 6, the samples were treated with excess Me_2_S·BH_3_. Silica gel chromatography gave the bis(borane) adducts (*in*,*in*/*out*,*out*)-3b, c, e·2BH_3_ (also prepared in Scheme 5) and *in*,*out*-3b, c, e·2BH_3_ in 43–23% and 42–16% yields, respectively. The latter were characterized analogously to the former,^32^ and representative ^13^C{^1^H} NMR spectra are compared in Fig. S9.† The phosphine boranes *in*,*out*-3b, c, e·2BH_3_ were deprotected using a standard protocol, neat refluxing pyrrolidine. Silica gel workups gave analytically pure *in*,*out*-3b, c, e in 77–56% yields as moderately air sensitive colorless oils.

### Additional probes of equilibria

**Fig. 2:**
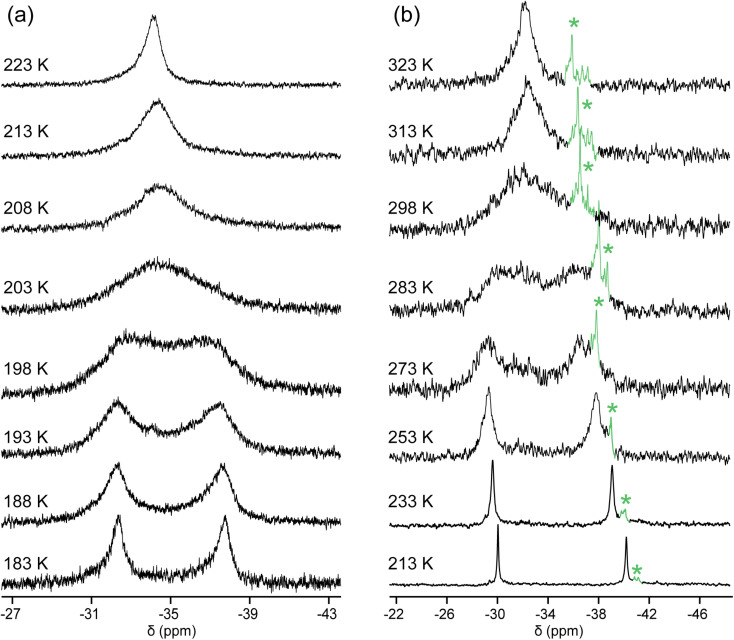
Variable temperature ^31^P{^1^H} NMR spectra (202 MHz) of (a) *in*,*out*-3c in CH_2_Cl_2_; (b) *in*,*out*-3b in CDCl_3_. The label * indicates an impurity.

Importantly, *in*,*out*-3b, c, e exhibited a single ^31^P{^1^H} NMR signal at ≥290 K, although by symmetry two would have been expected. This implies rapid homeomorphic isomerization (Scheme 6, upper right, or Scheme 2, lower manifold) on the NMR time scale. Accordingly, CH_2_Cl_2_ solutions were cooled, and ^31^P{^1^H} NMR spectra were recorded. In the cases of *in*,*out*-3b, c, two signals of nearly equal intensities decoalesced as shown in Fig. 2 (*T*_c_ = 290 K and 200 K). The data yielded Δ*G*^‡^_Tc_ values of 12.1 and 8.5 kcal mol^−1^, respectively. Thus, the activation energies increase as the macrocycles become smaller and degrees of freedom diminish.

The chemical shifts of the decoalesced signals, and the Δppm values, were similar to those in Fig. 1. Thus, the downfield signals are provisionally assigned to *in* bridgeheads, and the upfield signals to *out* bridgeheads. The ^31^P{^1^H} NMR spectra of the diphosphine diboranes (*in*,*in*/*out*,*out*)-3c·2BH_3_ and *in*,*out*-3c·2BH_3_, as well as ^1^H and ^13^C{^1^H} NMR spectra of the latter, were recorded over a similar temperature range in CD_2_Cl_2_ (Fig. S10–S13†). Although the chemical shifts and peak widths varied, there were no well-defined decoalescence phenomena.

### Crystal structures

**Fig. 3:**
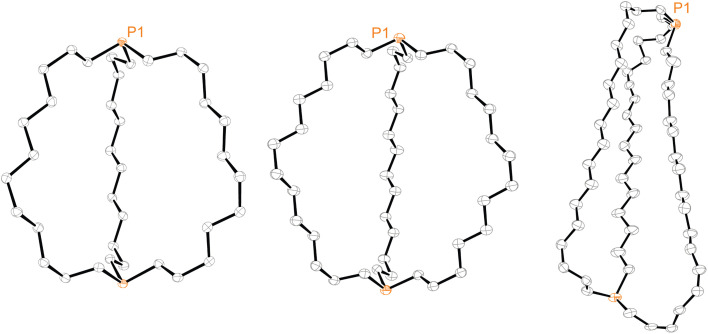
Thermal ellipsoid plots (50% probability level) for *out*,*out*-3b (left), *out*,*out*-3c (middle), and *out*,*out*-3e (right, dominant conformation).

Efforts were made to determine the crystal structures of as many of the preceding compounds as possible. Six were ultimately obtained as summarized in the ESI.† Key metrical parameters are presented in Table 1, and many additional distances and angles are tabulated in the ESI.† Interestingly, all compounds exhibited *out*,*out* geometries. Consider first the diphosphines *out*,*out*-3b, c, e, the thermal ellipsoid plots of which are compared in Fig. 3.

**Table 1:** Key crystallographic distances [Å] and angles [°]

	*out*,*out*-3b	*out*,*out*-3c	*out*,*out*-3e	*out*,*out*-3c·2BH_3_·(C_5_H_9_CH_3_)	*out*,*out*-3c·2BH_3_·(C_6_H_11_CH_3_)	*out*,*out*-3e·2BH_3_ (1)^a^	*out*,*out*-3e·2BH_3_ (2)^a^	*out*,*out*-3e·2BH_3_ (3)^a^
P–P^b^	10.8271(8)	12.948(3)	17.978(1)	13.212(4)	13.220(4)	19.407(3)	19.800(3)	19.540(3)
P–C	1.8535(15)	1.8361(15)	1.848(2)	1.840(11)	1.823(8)	1.825(8)	1.827(8)	1.805(8)
	1.8544(15)			1.87(3)	1.770(11)	1.827(8)	1.817(8)	1.802(8)
	1.8526(15)		1.832(2)	1.812(8)	1.929(12)	1.801(8)	1.814(8)	1.814(9)
	1.8541(16)			1.708(13)	1.837(10)	1.825(8)	1.822(9)	1.819(8)
	1.8530(15)		1.850(2)	1.92(3)	1.738(12)	1.819(8)	1.779(10)	1.825(9)
	1.8543(15)			1.852(10)	1.825(11)	1.831(8)	1.817(9)	1.830(8)
P–B	—	—	—	1.906(12)	1.864(12)	1.903(10)	1.903(10)	1.907(10)
				1.900(12)	1.881(12)	1.903(9)	1.920(10)	1.910(10)
C–P–C	98.67(7)	98.64(6)	102.06(10)	105.9(4)	105.7(5)	107.2(4)	105.8(4)	106.9(4)
	98.68(7)			102.0(16)	102.6(5)	103.9(4)	104.4(4)	101.7(4)
	98.02(7)		100.30(11)	106(2)	107.0(5)	106.6(4)	105.7(4)	110.0(4)
	97.65(7)			101.7(5)	108.5(6)	103.7(4)	105.8(5)	107.1(4)
	99.21(7)		100.32(10)	114.4(10)	105.0(5)	109.3(4)	107.1(4)	108.2(4)
	98.11(7)			97.1(11)	102.3(5)	107.8(4)	111.3(5)	103.3(4)
C–P–B	—	—	—	117.1(6)	115.9(5)	111.5(4)	112.2(4)	110.7(4)
				109.2(15)	120.4(6)	112.7(5)	112.8(4)	116.0(4)
				114.8(5)	103.7(5)	114.4(4)	115.1(4)	111.1(4)
				118.8(7)	112.8(6)	112.8(4)	106.4(5)	112.8(5)
				109.7(10)	116.5(6)	113.2(4)	113.9(5)	109.4(5)
				112.9(6)	110.7(6)	109.6(5)	112.2(5)	114.7(4)
P–P-lone pair or P–P–B	172.9	180.0	108.5	174.2(4)	176.1(4)	149.2(3)	153.0(3)	149.2(3)
	173.5			157.6(4)	158.4(4)	133.1(3)	129.2(4)	133.3(4)
Phosphorus pyramidalization^c^	295.4	295.9	302.7	313.9	315.3	317.7	315.9	318.6
	295.0			313.2	315.8	320.8	324.2	319.2

a The multiple columns refer to the three independent molecules in the unit cell.

b The corresponding value in the platinum complex *trans*-2c is 4.61 Å.

c Sum of the three C–P–C bond angles (360° = planar or sp^2^ limit, 328.4° = tetrahedral or sp^3^ limit; 270° = unhybridized p_*x*_/p_*y*_/p_*z*_ limit.

**Fig. 4:**
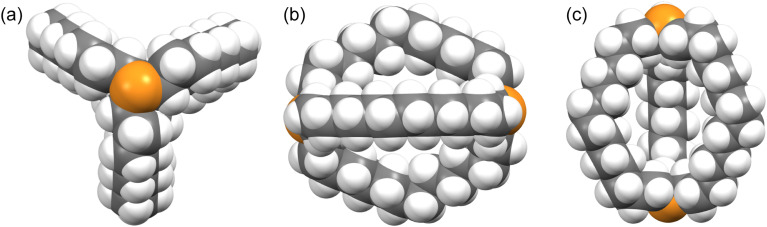
Space filling representations of *out*,*out*-3c from three orthogonal perspectives.

Crystalline *out*,*out*-3c exhibits unusual molecular symmetry (*D*_3_), such that the positions of all 44 non-hydrogen atoms can be defined from the atomic coordinates of only eight (P(CH_2_)_7_). As depicted in panel (a) of Fig. 4, a *C*_3_ axis passes through the two phosphorus atoms, and three *C*_2_ axes lie in a perpendicular plane (one of which runs perpendicular to the plane of the paper in panel (b)). However, the conformation remains chiral, with no internal mirror planes, although as required by the achiral space group *R*3̄*c*, both enantiomers are present in the unit cell. Panel (c) highlights the cage like nature and attendant interior space, which in the lattice is partially occupied by neighboring molecules (*vide infra*).

In the case of *out*,*out*-3b, there is a “near miss” with respect to *D*_3_ symmetry. Space filling views comparable to those in Fig. 4 are provided in Fig. S14,† and visually most deviations are slight. With *out*,*out*-3e (Fig. 3), a *C*_2_ axis passes through the midpoint of one P(CH_2_)_18_P chain and exchanges the phosphorus atoms and the other two chains. As detailed in the Experimental section, there is disorder in two chains but this is easily modeled and only the dominant conformation (77%) is treated. As may be facilitated by the larger ring sizes in *out*,*out*-3e, the chains partially collapse in on each other, stopping just short of van der Waals contacts and retaining a smidgen of interior space (Fig. S15†). Furthermore, the two phosphorus atoms no longer occupy geometric apices.

Features associated with the phosphorus–phosphorus vectors of *out*,*out*-3b, c, e are of interest. As summarized in Table 1, their lengths increase from 10.8271(8) to 12.948(3) to 17.978(1) Å. This constitutes an immense expansion of the phosphorus–phosphorus distances in the crystalline platinum complexes 2c, g (4.611–4.620 Å), emphasizing the structural flexibility of the diphosphines. The phosphorus–phosphorus-lone pair angles in *out*,*out*-3c are both 180°, whereas in *out*,*out*-3b they decrease slightly to 173.5°–172.9°. However, in *out*,*out*-3e the angles are both 108.5°, approaching the 90° cutoff for *out*,*out* and *in*,*in* isomers diagrammed in II (Scheme 1).

**Fig. 5:**
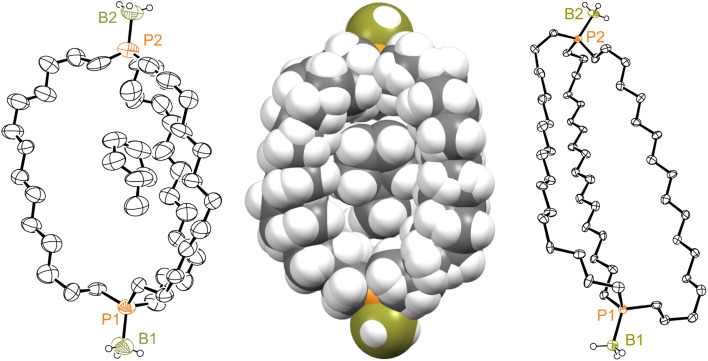
Thermal ellipsoid plots (50% probability level) for *out*,*out*-3c·2BH_3_·(C_5_H_9_CH_3_) (left, dominant conformation) and *out*,*out*-3e·2BH_3_ (right, one of three independent molecules in the unit cell), and a space filling representation of *out*,*out*-3c·2BH_3_·(C_5_H_9_CH_3_) (middle).

Consider next the diphosphine diboranes *out*,*out*-3c, e·2BH_3_. The former could be crystallized as both methylcyclopentane^33^ and methylcyclohexane monosolvates, *out*,*out*-3c·2BH_3_·(C_5_H_9_CH_3_) and *out*,*out*-3c·2BH_3_·(C_6_H_11_CH_3_). These exhibited very similar unit cell parameters (Table S1†) and molecular structures, with the solvent molecules occupying the interior of the diphosphine cages. The former (the better structure) is depicted in Fig. 5, but both are quite similar. Solvent is analogously incorporated into the diphosphine cages of crystalline digold complexes of the type *out*,*out*-3c·2AuX.^11,34^ The phosphorus–phosphorus vectors in *out*,*out*-3c·2BH_3_·(C_5_H_9_CH_3_) and *out*,*out*-3c·2BH_3_·(C_6_H_11_CH_3_) are 2.1% longer than that in the parent diphosphine *out*,*out*-3c (13.212(4)–13.220(4) Å *vs.* 12.948(3) Å), with one P–P–B angle close to 180° (174.2–176.1°) and the other somewhat smaller (157.6(4)–158.4(4)°).

Although *out*,*out*-3e·2BH_3_ crystallized without solvent incorporation, three independent molecules were present in the unit cell. One has been arbitrarily selected for Fig. 5. As with the congener *out*,*out*-3e, the cages in all three molecules have collapsed inward, with the P(CH_2_)_18_P chains close to but not quite in van der Waals contact. As expected, the phosphorus–phosphorus vectors (19.407(3)–19.800(3) Å) are much longer than those of the *out*,*out*-3c·2BH_3_ monosolvates, and for conformational reasons longer than that in *out*,*out*-3e (17.978(1) Å). The P–P–B angles (153.0(3)–129.2(4)°; avg 141.2°) are smaller than the P–P–(B or lone pair) angles of all the compounds except *out*,*out*-3e (108.5°).

### Crystal lattices

**Fig. 6:**
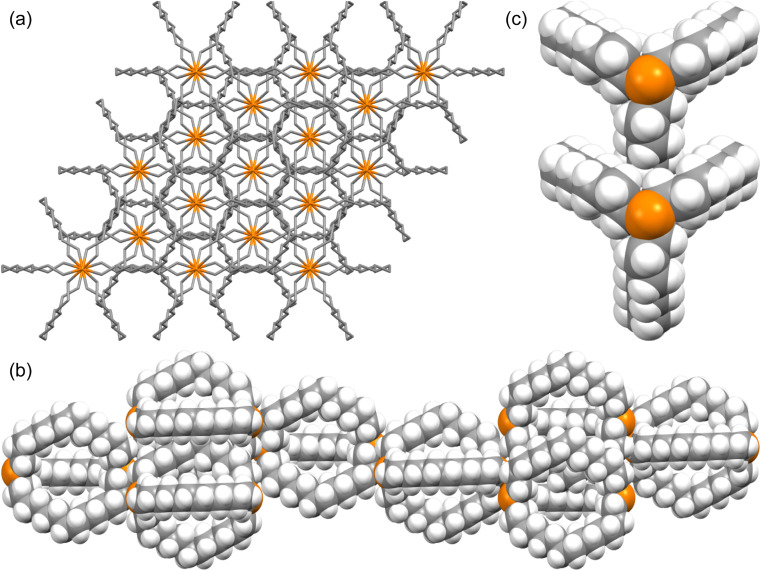
Views of the crystal lattice of *out*,*out*-3c with the long *c* axis perpendicular to the plane of the paper (a and c) or in the plane of the paper (b).

The unexpected uniformity with which the preceding compounds crystallized as *out*,*out* isomers prompted close inspections of the crystal lattices for potential “packing forces”. The crystal systems exhibited by *out*,*out*-3b, c, e (triclinic, rhombohedral, monoclinic; *Z* = 2, 6, 4) differed. However, the unit cells always featured one axis that was very much longer than the others (Table S1†), as particularly pronounced for *out*,*out*-3c (*c* = 89.58(2) Å *vs.* 2 × 9.1903(19) Å). When viewed along this axis, the phosphorus–phosphorus vectors are aligned, as highlighted in orange in panel (a) of Fig. 6. Each stack is surrounded by six others, all equidistant and in a hexagon motif.

There are six (CH_2_)_14_ segments radiating with 60° spacings from each phosphorus–phosphorus stack, the PCH_2_ segments of which generate apparent rhomboids, followed by “tails” representing the remaining CH_2_ groups. Three are associated with one layer of molecules aligned along the *c* axis, and the other three with a layer that is three layers above or below (Fig. 6, panel (b)). Within each layer, the (CH_2_)_14_ bridge of one molecule intercalates between two (CH_2_)_14_ bridges of a neighboring molecule (panel (c)). Between adjacent layers, the molecules are offset, the phosphorus atoms of one abutting the 
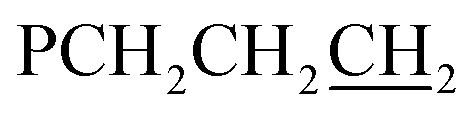
 groups of the other. However, the closest intermolecular distances fall just short of van der Waals contacts.

**Fig. 7:**
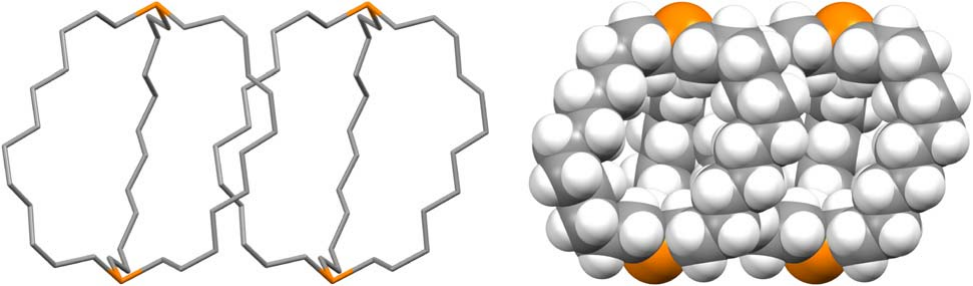
Two adjacent molecules in the crystal lattice of *out*,*out*-3b.

Although *out*,*out*-3b does not give as symmetrical a lattice as *out*,*out*-3c, the (CH_2_)_12_ chains similarly nest within the interstices generated by two (CH_2_)_12_ chains of a neighboring molecule (Fig. 7). The two bis(borane) adducts *out*,*out*-3c·2BH_3_·(C_5_H_9_CH_3_) and *out*,*out*-3c·2BH_3_·(C_6_H_11_CH_3_) pack similarly. Here, the interior solvent molecules require expression as *out*,*out* isomers, so crystal packing forces cannot be playing a direct role.

**Fig. 8:**
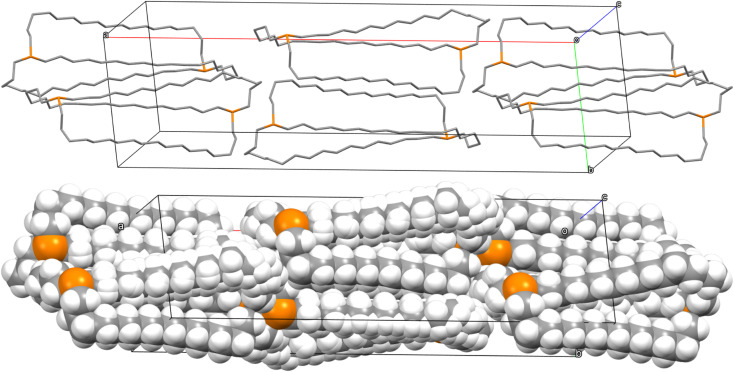
Adjacent molecules in the crystal lattice of *out*,*out*-3e (dominant conformations only).

The long dimensions of the P(CH_2_)_18_P systems *out*,*out*-3e and *out*,*out*-3e·2BH_3_ are also roughly aligned in the respective crystal lattices. However, as illustrated in Fig. 8, there is no intercalation as in Fig. 6. Nonetheless, the *inter*molecular spacings between (CH_2_)_18_ chains are comparable to the intramolecular spacings, which as noted above are slightly greater than van der Waals contacts. Interestingly, *out*,*out*-3e and *out*,*out*-3e·2BH_3_ exhibit the highest densities in each series (*e.g.*, *ρ* 1.022 (*out*,*out*-3e) *vs.* 0.991–0.990 (*out*,*out*-3b, c) Mg m^−3^).

## Discussion

### Syntheses of dibridgehead diphosphines

**Scheme 7:**
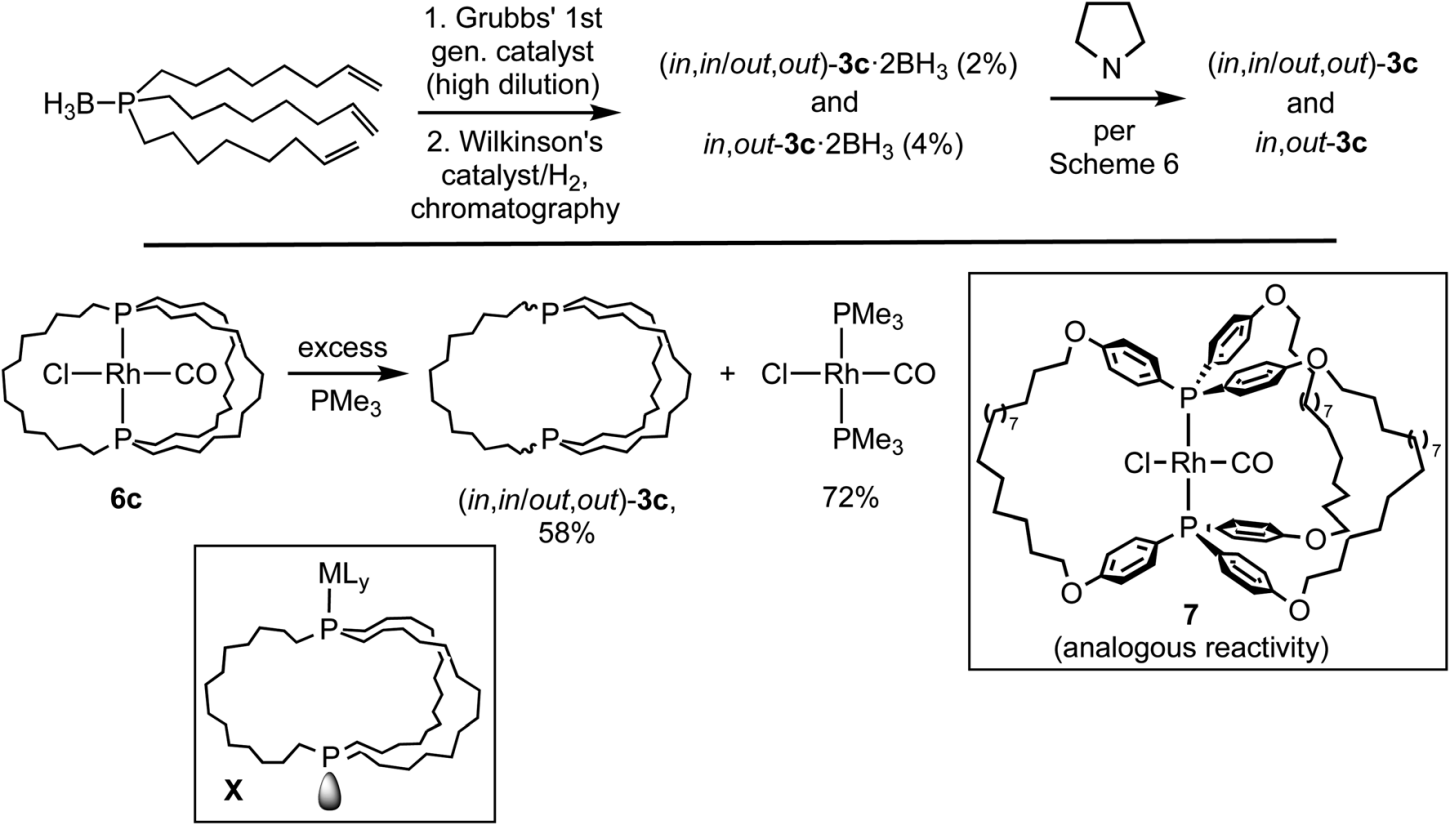
Additional routes to the title molecules and related species.

As shown in Scheme 3, this study has established the synthetic availability of an extensive family of aliphatic macrocyclic dibridgehead diphosphines P((CH_2_)_*n*_)_3_P (3) that can exist as *in*,*in*, *out*,*out*, and *in*,*out* isomers. There currently seems to be no obstacle to extending this chemistry to *n* ≥ 20, or ≥42 membered macrocycles. As depicted in Scheme 7 (top), isomers of 3c (*n* = 14) have also been accessed *via* three-fold intermolecular olefin metatheses of the metal-free phosphine borane H_3_B·P((CH_2_)_6_CH=CH_2_)_3_.^32^ However, the metathesis steps proceed in much lower yields than the platinum templated pathway, and the overall yields are miniscule.

As exemplified by 6c in Scheme 7 (bottom), demetallation of gyroscope-like rhodium(i) complexes can also be effected,^35^ but in efforts to date yields have not been superior to the routes in Scheme 3. Precursors such as 7 afford related dibridgehead di(triaryl)phosphines.^35*b*^ However, there have recently been promising developments regarding alternative routes that involve inexpensive metals.^28^ These have included the iron-based syntheses of the dibridgehead diarsines As((CH_2_)_*n*_)_3_As (*n* = 10, 12, 14).^36^

The mechanisms of these demetalations, which are receiving ongoing attention, are beyond the scope of this study. However, since metal fragments can generally be reintroduced (Scheme 4), intermediates such as X (Scheme 7) derived from metal–phosphorus bond cleavage and homeomorphic isomerization have been suggested. The platinum byproducts M_2_Pt(C≡X)_4_ generated in Scheme 3 would be derived from the displacement of all the metal–ligand bonds in X by –C≡X nucleophiles. Accordingly, excess KC≡N has been shown to convert various *cis*-PtCl_2_(diphosphine) adducts to K_2_Pt(C≡N)_4_.^23^

**Scheme 8:**
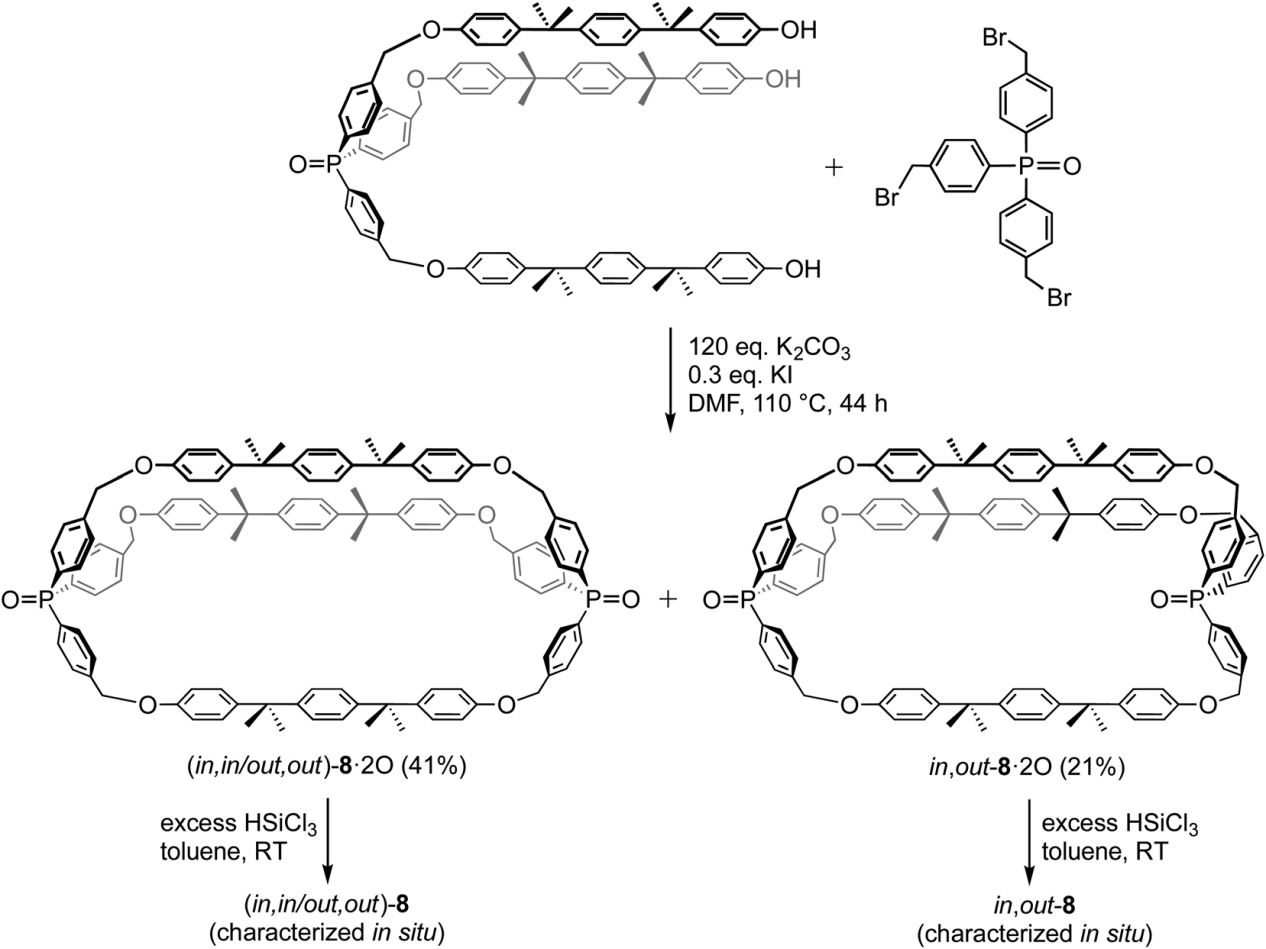
Macrocyclic dibridgehead diphosphorus compounds reported by Bauer.^17*b*^

The most closely related dibridgehead diphosphorus macrocycles in the literature are depicted in Scheme 8.^17*b*^ The dibridgehead di(triaryl)phosphine dioxides 8·2O were constructed *via* Williamson ether syntheses that afforded both (*in*,*in*/*out*,*out*) and *in*,*out* isomers. Subsequent reductions gave 8, which were characterized *in situ* due to their air sensitivity. It has not yet proved possible to quantify equilibrium ratios or obtain crystal structures for any of these species. Nonetheless, the *in*/*out* isomers could be assigned based upon rate trends in Scheme 8 and derivatization reactions. Recently, several additional types of novel compounds exhibiting *in*/*out* isomerism have been reported.^15,37^

### Isomer assignments

In certain cases, unequivocal isomer assignments are possible for the preceding compounds. For example, the thermal epimerisation of 3b, c, e in Scheme 4 give species that can exhibit two ^31^P{^1^H} NMR signals of equal areas at low temperatures. These can only be *in*,*out* isomers that undergo rapid homeomorphic isomerization. The other isomers must therefore represent the *in*,*in*/*out*,*out* manifold as mapped in Scheme 2. Similarly, there are two cases in which the *in*,*in*/*out*,*out* isomers exhibit two ^31^P{^1^H} NMR signals (unequal areas) at low temperature. One of these must represent an *in*,*in* isomer, and the other *out*,*out*. Our rationale for assigning the dominant isomer as *in*,*in*, which is perhaps counterintuitive, is as follows.

First, the earliest computational probes of such equilibria, conducted with the hydrocarbons HC((CH_2_)_*n*_)_3_CH, pointed to an increasing and eventually dominant proportion of *in*,*in* isomers as *n* is increased.^29^ Second, DFT calculations with (P(CH_2_)_*n*_)_3_P (*n* = 8–20) have always given parallel results.^10,21^ As reported separately, DFT has also been used to compute the ^31^P NMR chemical shifts (*n* = 10–20),^10*b*,20^ and two relationships emerge: (1) the chemical shifts of the *in*,*in* isomers are downfield of the *out*,*out* isomers, and (2) the chemical shifts of the *in* bridgeheads of the *in*,*out* isomers are downfield of the *out* bridgeheads (Δppm similar to experiment in both cases).^27^ Inverting the assignments made above would contradict these findings.

Molecular dynamics simulations have also been carried out.^10^ These indicate an abundance of conformers that are relatively closely spaced in energy for all limiting isomers. Several factors point to increased attractive intramolecular dispersion (van der Waals) forces in the *in*,*in* as opposed to *out*,*out* isomers. For example, a reduced surface area should translate into a reduced void space in these cage-like structures. The latter should in turn increase the dispersive (attractive) van der Waals forces between methylene linkers. Accordingly, the Connolly contact surfaces^38^ generated using various probe radii (*e.g.*, 4.0 Å, comparable to a small solvent molecule) are on the average lower for the ensemble of low-energy conformations for the *in*,*in* isomers. We note in passing that additional computational studies have addressed other aspects of *in*/*out* isomerism.^12,15,37^

### Further analyses of equilibria and dynamic properties

The *in*,*in* and *out*,*out* equilibrium ratios for 3b, c, 86 : 14 and 97 : 3 (213 K and 193 K, toluene-*d*_8_), correspond to small Δ*G* values (0.77, 1.33 kcal mol^−1^) at the temperatures of measurement. Importantly, the proportion of *out*,*out* isomer increases in the smaller macrocycle,^27^ as would be expected as a limiting bicyclo[2.2.2]octane-based structure such as DABCO is approached. The equilibrium ratios of (*in*,*in*/*out*,*out*)-3b, c, e*versus in*,*out*-3b, c, e, as established at 150 °C (Scheme 6), are not very dependent upon the macrocycle size. The slightly greater bias of 3b towards the (*in*,*in*/*out*,*out*)-isomers (60 : 40 *vs.* 51 : 49) is consistent with a small decrease in the relative stabilities of *in*,*out* isomers with decreasing ring sizes.

The Δ*G*^‡^ values for the homeomorphic isomerization of *out*,*out*-3 to *in*,*in*-3 increase as the ring sizes decrease (10.4 kcal mol^−1^ for 3c, 193 K *vs.* 11.4 kcal mol^−1^ for 3b, 213 K). Importantly, the Δ*S*^‡^ values become more negative as the ring sizes decrease (−11.8 eu, 3c; −19.4 eu, 3b). This is consistent with a more pronounced loss of degrees of freedom in turning the smaller macrocycle inside out. However, the Δ*H*^‡^ values decrease (8.2 kcal mol^−1^, 3c; 7.3 kcal mol^−1^, 3b), hinting at a possible isokinetic relationship.^39^ Interestingly, the Δ*G*^‡^ value for the degenerate homeomorphic isomerization of *in*,*out*-3c (8.5 kcal mol^−1^, 200 K) is less than those for the non-degenerate isomerizations of *in*,*in*-3c and *out*,*out*-3c (10.4 or 11.5 kcal mol^−1^, 193 K, depending upon direction). The trend for *in*,*out*-3b compared to *in*,*in*- and *out*,*out*-3b is analogous but less pronounced (290 K: 12.1 *vs.* 13.1 or 13.8 kcal mol^−1^).

The epimerisations of (*in*,*in*/*out*,*out*)-3b, c, e (Scheme 6) require similar temperatures and time scales. As noted above, the Δ*G*^‡^ value for (*in*,*in*/*out*,*out*)-3c (34.4 kcal mol^−1^, 150 °C or 423 K) is typical for inversions of acyclic trialkylphosphines. This suggests a common bridgehead inversion mechanism, as opposed to unconventional pathways involving phosphorus–phosphorus interactions that could show a dependency upon macrocycle size. Reactions of dibridgehead diphosphines with P(CH_2_)_*n*_P linkages with *n* ≤ 4 can afford species with phosphorus–phosphorus bonds, for which inversions at phosphorus have been documented.^40^

**Scheme 9:**
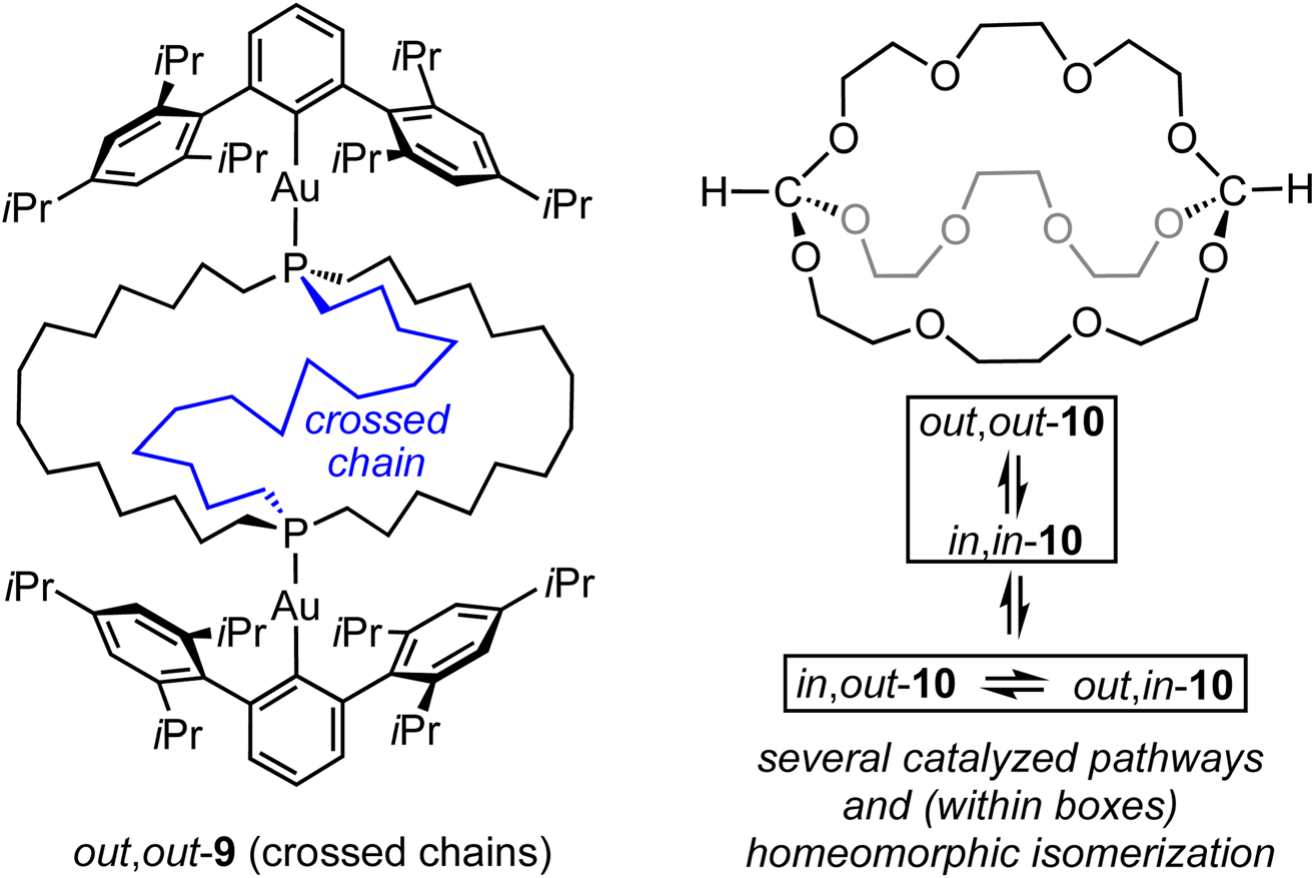
Additional types of compounds exhibiting *in*/*out* isomerism.

Of course, all the same questions can be posed with regard to the diphosphine diboranes 3·2BH_3_. These are beyond the scope of this paper, but it is clear that equilibria involving *in*,*in* and *out*,*out* isomers, or degenerate *in*,*out* species, remain rapid on NMR time scales. Despite the greater steric demand of a bridgehead BH_3_ substituent as opposed to a lone pair, we believe that the diphosphines in this study provide ample clearance for *in*,*in* and *in*,*out* isomers. However, when the BH_3_ groups of *in*,*out*-3c·2BH_3_ are replaced by bulky gold(i) Lewis acids AuAr as in 9 (Scheme 9), steric interactions greatly increase. Accordingly, the structure “flips” into an *out*,*out* form with crossed chains (see VIII, Scheme 2), both in solution and the solid state.^11^

Finally, it should be kept in mind that a wide variety of mechanisms may be operative in *in*/*out* isomerizations. For example, dibridgehead diorthoesters with (OCH_2_CH_2_)_*n*_ bridges have recently been prepared, as exemplified by 10 in Scheme 9.^15^ All three *in*/*out* orientations of the HC(OR)_3_ units can be observed. These can equilibrate by both homeomorphic isomerizations and catalyzed pathways involving cleavage of the carbon–oxygen bonds.

### Crystal structures

As shown in Fig. 3, the dibridgehead diphosphines 3b, c, e all crystallize as *out*,*out* isomers, despite the conclusions in the preceding sections that *in*,*in* isomers dominate in solution. There are many cases where a compound crystallizes as the less stable of two possible isomers, which is commonly attributed to packing forces. However, this is rarely observed for an entire series of compounds. In the case of *out*,*out*-3c, Fig. 6 convincingly documents a highly symmetric molecular structure (*D*_3_) that affords an exceptional lattice with optimal intermolecular contacts as analyzed above. The dibridgehead diarsine As((CH_2_)_14_)_3_As crystallizes analogously.^41^

Crystalline *out*,*out*-3b as well as the arsenic homolog As((CH_2_)_12_)_3_As^36^ are just a few CH_2_–CH_2_ rotations removed from *D*_3_ symmetry, and share several packing features with *out*,*out*-3c (*e.g.*, Fig. 7). As shown in Fig. 8, *out*,*out*-3e crystallizes in a much different motif, but still with a visually impressive degree of van der Waals contacts or near-contacts. In any event, these apparently trump the greater intramolecular dispersion forces posited for individual *in*,*in* isomers. Nonetheless, this dichotomy remains an obvious focus for further study and interpretation.

The bridgehead phosphorus atoms in *out*,*out*-3b, c, e are much more pyramidalized than in *out*,*out*-3c, e·2BH_3_, as evidenced by the sums of the three carbon–phosphorus–carbon bond angles (295.0–302.7° *vs.* 313.2–324.3°; Table 1). The solvent occupied cages in the two structures of *out*,*out*-3c·2BH_3_ raise the issue of whether any of the equilibria or other phenomena described above might be affected by encapsulated solvent. We view the solvate molecules as simple consequences of crystal growth, as our gyroscope-like complexes (*e.g.*, *trans*-2g in Scheme 3)^18*b*^ occasionally but by no means routinely crystallize with a solvent molecule within a macrocycle.^34^ Also, attempts to detect toluene-*d*_*n*_ adducts of *in*,*in*-3c or *out*,*out*-3c at low temperatures in CD_2_Cl_2_ and other solvents have been unsuccessful.

## Conclusion

This study has brought heretofore unavailable definition to equilibria and dynamic and configurational processes involving the most fundamental type of large-ring aliphatic bicyclic compounds with bridgehead heteroatoms, XE((CH_2_)_*n*_)_3_EX. The new dibridgehead diphosphines 3b–e are highly flexible with extensive manifolds of conformations and coordination modes. They represent very promising building blocks for both monometallic and polymetallic or polymeric systems – research directions that will be facilitated as improved syntheses are developed.^28^ Monometallic adducts of *in*,*in* isomers have been termed gyroscope-like and are attractive springboards for molecular gyroscopes.^42^ The dynamic properties of 3c, e have already been put to use in metal transport protocols,^5^ and all of the preceding directions of inquiry are actively being extended to diarsine^36,41^ and phosphine oxide analogs (EX = As, PO).^13^ These and related themes will be the subject of future reports from this laboratory.

## Experimental section

### (*in*,*in*/*out*,*out*)-P((CH_2_)_12_)_3_P ((*in*,*in*/*out*,*out*)-3b)

A Schlenk flask was charged with *cis*-PtCl_2_(P((CH_2_)_12_)_3_P (*cis*-2b;^19^ 0.1010 g, 0.1213 mmol), KCN (0.097 g, 1.489 mmol), THF (15 mL) and degassed water (0.5 mL). The mixture was stirred (24 h). The solvent was removed from the filtrate by oil pump vacuum. The residue was filtered through a pad of silica (1.5 × 1 cm). The filter cake was washed with CH_2_Cl_2_ (2 × 10 mL). The solvent was removed from the filtrate by oil pump vacuum to give (*in*,*in*/*out*,*out*)-3b (0.0488 g, 0.0861 mmol, 71%) as a white solid, mp 52–55 °C. Anal. calcd for C_36_H_72_P_2_ (566.90): C, 76.27; H, 12.80; found: C, 76.49; H, 12.82.

NMR (CDCl_3_, *δ*/ppm): ^1^H (500 MHz) 1.46–1.35, 1.35–1.25 (2 br m, 72H, C*H̲*_2_); ^13^C{^1^H} (126 MHz) 30.9 (d, *J*_CP_ = 9.5 Hz, *C̲*H_2_), 28.5 (s, *C̲*H_2_), 28.4 (s, *C̲*H_2_), 28.1 (s, *C̲*H_2_), 26.3–25.9 (br s, *C̲*H_2_), 25.0–24.7 (br s, *C̲*H_2_); ^31^P{^1^H} (202 MHz) −29.7 to −32.1 (br s).

NMR (toluene-*d*_8_, *δ*/ppm, 373 K): ^1^H (500 MHz) 1.45–1.35, 1.35–1.23 (2 br m, 72H, C*H̲*_2_); ^13^C{^1^H} (126 MHz) 30.8 (d, *J*_CP_ = 10.1 Hz, *C̲*H_2_), 28.71 (s, *C̲*H_2_), 28.70 (s, *C̲*H_2_), 28.67 (s, *C̲*H_2_), 28.3 (d, *J*_CP_ = 14.6 Hz, *C̲*H_2_), 26.0 (d, *J*_CP_ = 13.4 Hz, *C̲*H_2_); ^31^P{^1^H} (202 MHz) −31.9 (br s).

### (*in*,*in*/*out*,*out*)-P((CH_2_)_14_)_3_P ((*in*,*in*/*out*,*out*)-3c)

A Schlenk flask was charged with *trans*-PtCl_2_(P((CH_2_)_14_)_3_P (*trans*-2c;^18*a*^ 0.4356 g, 0.475 mmol), KCN (0.4674 g, 7.177 mmol), THF (20 mL) and degassed water (0.5 mL). The mixture was stirred (24 h) and filtered to remove a yellow precipitate. The solvent was removed from the filtrate by oil pump vacuum to give *in*,*in*/*out*,*out*-3c (0.2692 g, 0.411 mmol, 87%) as a white solid, mp 68 °C. Anal. calcd for C_42_H_84_P_2_ (651.06): C, 77.48; H, 13.00; found: C, 77.67; H, 13.08.

NMR (CDCl_3_, *δ*/ppm): ^1^H (500 MHz) 1.37–1.32, 1.31–1.23 (2 br m, 84H, C*H̲*_2_); ^13^C{^1^H} (126 MHz) 31.2 (d, *J*_CP_ = 10.3 Hz, *C̲*H_2_), 29.2 (s, *C̲*H_2_), 29.14 (s, *C̲*H_2_), 29.08 (s, *C̲*H_2_), 28.7 (s, *C̲*H_2_), 26.3 (d, *J*_CP_ = 12.2 Hz, *C̲*H_2_), 25.2 (d, *J*_CP_ = 11.0 Hz, *C̲*H_2_); ^31^P{^1^H} (202 MHz) −30.1 (s).

A ^13^C{^1^H} NMR spectrum of the yellow precipitate showed signals (D_2_O, *δ*/ppm) for K_2_Pt(CN)_4_ (126.5; lit^43^ 126.5) and KCN (166.8). The precipitate was dissolved in water (5 mL) and the solution allowed to slowly concentrate. After 7 d, thin colorless plates of K_2_Pt(CN)_4_ were obtained, as verified by X-ray crystallography.^44^

### (*in*,*in*/*out*,*out*)-P((CH_2_)_16_)_3_P ((*in*,*in*/*out*,*out*)-3d)

A Schlenk flask was charged with *cis*-PtCl_2_(P((CH_2_)_16_)_3_P (*cis*-2d;^19^ 0.2453 g, 0.245 mmol), KCN (0.2393 g, 3.675 mmol), THF (15 mL), and degassed water (0.5 mL). The mixture was stirred. After 24 h, the mixture was filtered. The filter cake was washed with THF (2 × 5 mL). The solvent was removed from the filtrate by oil pump vacuum, and CH_2_Cl_2_ (25 mL) added to the solid residue. The sample was filtered through a pad of silica (1.5 × 1 cm). The filter cake was washed with CH_2_Cl_2_ (2 × 10 mL). The solvent was removed from the filtrate by oil pump vacuum to give (*in*,*in*/*out*,*out*)-3d (0.1476 g, 0.201 mmol, 82%) as a white solid, mp 56–59 °C.

NMR (CDCl_3_, *δ*/ppm) ^1^H (500 MHz) 1.46–1.33, 1.33–1.23 (2 br m, 96H, C*H̲*_2_); ^13^C{^1^H} (126 MHz) 31.2 (d, *J*_CP_ = 10.2 Hz, *C̲*H_2_), 29.2 (s, 2 × *C̲*H_2_), 29.1 (s, *C̲*H_2_), 29.0 (s, *C̲*H_2_), 28.8 (s, *C̲*H_2_), 26.6 (d, *J*_CP_ = 12.0 Hz, *C̲*H_2_), 25.4 (d, *J*_CP_ = 11.6 Hz, *C̲*H_2_); ^31^P{^1^H} (202 MHz) −30.7 (s).

### (*in*,*in*/*out*,*out*)-P((CH_2_)_18_)_3_P ((*in*,*in*/*out*,*out*)-3e)

A Schlenk flask was charged with *trans*-PtCl_2_(P((CH_2_)_18_)_3_P (*trans*-2e;^18^ 0.2529 g, 0.233 mmol), KCN (0.2319 g, 3.561 mmol), THF (15 mL), and degassed water (0.5 mL) with stirring. After 24 h, the mixture was filtered. The filter cake was washed with THF (2 × 5 mL). The solvent was removed from the filtrate by oil pump vacuum, and CH_2_Cl_2_ (25 mL) added to the solid residue. The sample was filtered through a pad of silica (1.5 × 1 cm). The filter cake was washed with CH_2_Cl_2_ (2 × 10 mL). The solvent was removed from the filtrate by oil pump vacuum to give (*in*,*in*/*out*,*out*)-3e (0.1622 g, 0.198 mmol, 85%) as a white solid, mp 54–57 °C. Anal. calcd for C_54_H_108_P_2_ (819.38): C, 79.15; H, 13.29; found: C, 79.16; H, 13.48.

NMR (C_6_D_6_, *δ*/ppm): ^1^H (500 MHz) 1.60–1.52 (br m, 12H, C*H̲*_2_), 1.49–1.40 (br m, 24H, C*H̲*_2_), 1.40–1.29 (br m, 72H, C*H̲*_2_); ^13^C{^1^H} (126 MHz) 32.1 (d, *J*_CP_ = 10.5 Hz, *C̲*H_2_), 30.34 (s, *C̲*H_2_), 30.32 (s, *C̲*H_2_), 30.3 (s, *C̲*H_2_), 30.2 (s, *C̲*H_2_), 30.1 (s, *C̲*H_2_), 30.0 (s, *C̲*H_2_), 28.4 (d, *J*_CP_ = 13.6 Hz, *C̲*H_2_), 26.8 (d, *J*_CP_ = 13.0 Hz, *C̲*H_2_); ^31^P{^1^H} (202 MHz) −32.8 (s). IR (cm^−1^, powder film): 2916 (s), 2847 (s), 1466 (s), 1442 (s), 725 (s).

### (*in*,*in*/*out*,*out*)-3b·2BH_3_

A Schlenk flask was charged with (*in*,*in*/*out*,*out*)-3b (0.0705 g, 0.124 mmol) and THF (10 mL). Then Me_2_S·BH_3_ (2.0 M in THF; 0.13 mL, 0.26 mmol) was added with stirring. After 1 d, the solvent was removed by oil pump vacuum. The residue was chromatographed on a silica column (4 × 30 cm) using hexanes/CH_2_Cl_2_ (1 : 2 v/v). The solvents were removed from the product fractions by oil pump vacuum to give (*in*,*in*/*out*,*out*)-3b·2BH_3_ (0.0521 g, 0.0876 mmol, 70%) as a light yellow oil. Anal. calcd for C_36_H_78_B_2_P_2_ (594.57): C, 72.72; H, 13.22; found: C, 72.72; H, 13.01.

NMR (CDCl_3_, *δ*/ppm): ^1^H (500 MHz) 1.63–1.53 (m, 12H, C*H̲*_2_), 1.53–1.44 (m, 12H, C*H̲*_2_), 1.41–1.37 (m, 12H, C*H̲*_2_), 1.37–1.23 (m, 36H, C*H̲*_2_), 0.48 and 0.27 (br apparent d, 6H, B*H̲*_3_); ^13^C {^1^H} (126 MHz) 30.3 (d, *J*_CP_ = 11.9 Hz, *C̲*H_2_), 28.9 (s, *C̲*H_2_), 28.5 (s, *C̲*H_2_), 28.1 (s, *C̲*H_2_), 22.4 (d, *J*_CP_ = 34.0 Hz, *C̲*H_2_), 21.9 (d, *J*_CP_ = 2.9 Hz, *C̲*H_2_); ^31^P{^1^H} (202 MHz): 15.0–13.7 (br s).^31*b*^

### (*in*,*in*/*out*,*out*)-3c·2BH_3_

A J. Young NMR tube was charged with *in*,*in*/*out*,*out*-3c (0.0271 g, 0.0416 mmol), CDCl_3_ (0.6 mL), and Me_2_S·BH_3_ (0.20 mL, 2.0 M in THF, 0.40 mmol). After 1 d, the solvent was removed *in vacuo*. The residue was passed through silica gel (1 × 15 cm) using hexanes/CH_2_Cl_2_ (2 : 1 v/v). The solvent was removed *in vacuo* to give *in*,*in*/*out*,*out*-3c·2BH_3_ (0.0211 g, 0.0311 mmol, 75%) as a white solid, mp 112 °C. DSC (*T*_i_/*T*_e_/*T*_p_/*T*_c_/*T*_f_): 95.8/110.6/112.7/114.2/117.5 °C (endotherm). TGA: onset of mass loss, 282 °C. Anal. calcd for C_42_H_90_B_2_P_2_ (678.73): C, 74.32; H, 13.37; found C, 74.01; H, 13.08.

NMR (CDCl_3_, *δ*/ppm): ^1^H (500 MHz) 1.60–1.49 (m, 12H, C*H̲*_2_), 1.50–1.41 (m, 12H, C*H̲*_2_), 1.41–1.32 (m, 12H, C*H̲*_2_), 1.32–1.20 (m, 48H, C*H̲*_2_), 0.38 and 0.26 (br apparent d, 6H, B*H̲*_3_); ^13^C{^1^H} (126 MHz) 30.7 (d, *J*_CP_ = 12.1 Hz, *C̲*H_2_), 29.31 (s, *C̲*H_2_), 29.25 (s, *C̲*H_2_), 29.0 (s, *C̲*H_2_), 28.5 (s, *C̲*H_2_), 22.5 (d, *J*_CP_ = 34.1 Hz, *C̲*H_2_), 22.2 (d, *J*_CP_ = 2.6 Hz, *C̲*H_2_); ^31^P{^1^H} (202 MHz) 16.2–14.4 (br s).^31*b*^ IR (cm^−1^, powder film): 2922 (s), 2853 (s), 2366 (m), 1467 (w), 1061 (m), 718 (m). MS (EI): 678 (M^+^, 1%), 665 ([M − BH_3_]^+^, 38%), 651 ([M − 2BH_3_]^+^, 100%).

### (*in*,*in*/*out*,*out*)-3e·2BH_3_

A Schlenk flask was charged with (*in*,*in*/*out*,*out*)-3e (0.2147 g, 0.262 mmol) and THF (15 mL). Then Me_2_S·BH_3_ (2.0 M in THF; 0.65 mL, 1.3 mmol) was added with stirring. After 1 d, the solvent was removed by oil pump vacuum. The residue was chromatographed on a silica column (4 × 46 cm) using hexanes/CH_2_Cl_2_ (1 : 0 to 0 : 1 v/v). The solvents were removed from the product fractions by oil pump vacuum to give (*in*,*in*/*out*,*out*)-3e·2BH_3_ (0.2027 g, 0.239 mmol, 91%) as a white powder, mp 67–69 °C. Anal. calcd for C_54_H_114_P_2_B_2_ (847.07): C, 76.57; H, 13.57; found: C, 76.28; H, 13.62.

NMR (CDCl_3_, *δ*/ppm): ^1^H (500 MHz): 1.60–1.52 (m, 12H, C*H̲*_2_), 1.52–1.42 (m, 12H, C*H̲*_2_), 1.42–1.34 (m, 12H, C*H̲*_2_), 1.34–1.21 (m, 72H, C*H̲*_2_), 0.45 and 0.28 (br apparent d, 6H, B*H̲*_3_); ^13^C{^1^H} (126 MHz): 31.0 (d, *J*_CP_ = 12.0 Hz, *C̲*H_2_), 29.60 (s, *C̲*H_2_), 29.58 (s, *C̲*H_2_), 29.50 (s, *C̲*H_2_), 29.45 (s, *C̲*H_2_), 29.2 (s, *C̲*H_2_), 28.8 (s, *C̲*H_2_), 22.8 (d, *J*_CP_ = 34.2 Hz, *C̲*H_2_), 22.4 (d, *J*_CP_ = 2.7 Hz, *C̲*H_2_); ^31^P{^1^H} 14.9–13.6 (br s).^31*b*^ IR (cm^−1^, powder film): 2915 (s), 2846 (m), 2361 (m), 1468 (m), 1063 (m), 716 (m).

### Epimerization of 3b; *in*,*out*-3b·2BH_3_ and (*in*,*in*/*out*,*out*)-3b·2BH_3_

A Schlenk flask was charged with (*in*,*in*/*out*,*out*)-3b (0.0488 g, 0.0861 mmol) and mesitylene (10 mL). The solution was stirred at 150 °C. After 60 h, a ^31^P{^1^H} NMR spectrum showed a 60 : 40 (*in*,*in*/*out*,*out*)-3b/*in*,*out*-3b mixture. The solvent was removed by oil pump vacuum, the residue dissolved in THF (15 mL), and Me_2_S·BH_3_ (2.0 M in THF; 0.30 mL, 0.60 mmol) added with stirring. After 24 h, the solvent was removed by oil pump vacuum. The residue was chromatographed on a silica column (1 × 26 cm) using hexanes/CH_2_Cl_2_ (1 : 1 v/v). The solvents were removed from the product fractions by oil pump vacuum. First *in*,*out*-3b·2BH_3_ eluted (colorless oil, 0.0131 g, 0.0220 mmol, 26%). Anal. calcd for C_36_H_72_P_2_ (594.57): C, 72.72; H, 13.22; found: C, 73.02; H, 13.13.

NMR (CDCl_3_, *δ*/ppm): ^1^H (500 MHz) 1.64–1.54 (m, 12H, C*H̲*_2_), 1.54–1.45 (m, 12H, C*H̲*_2_), 1.45–1.37 (m, 12H, C*H̲*_2_), 1.37–1.24 (m, 36H, C*H̲*_2_), 0.51 and 0.29 (br apparent d, 6H, B*H̲*_3_); ^13^C{^1^H} (126 MHz) 30.4 (d, *J*_CP_ = 11.2 Hz, *C̲*H_2_), 27.7 (s, *C̲*H_2_), 27.6 (s, *C̲*H_2_), 27.5 (s, *C̲*H_2_), 23.4 (d, *J*_CP_ = 34.3 Hz, *C̲*H_2_), 22.2 (s, *C̲*H_2_); ^31^P{^1^H} (202 MHz): 15.6–14.1 (br s).^31*b*^

Next (*in*,*in*/*out*,*out*)-3b·2BH_3_ eluted (colorless oil, 0.0194 g, 0.326 mmol, 38%). The NMR data agreed with that from the independent synthesis above.

### *in*,*out*-P((CH_2_)_12_)_3_P (*in*,*out*-3b)

A Schlenk flask was charged with *in*,*out*-3b·2BH_3_ (0.0070 g, 0.0117 mmol) and pyrrolidine (2 mL). The mixture was refluxed (60 °C) for 3 d. The solvent was removed by oil pump vacuum. Toluene (5 mL) was added, and the suspension passed through a pad of silica gel using a pipette (0.7 × 3 cm). The filter cake was washed with toluene (10 mL). The solvent was removed from the filtrate by oil pump vacuum to give *in*,*out*-3b (0.0052 g, 0.009 mmol, 77%) as a colorless oil.

NMR (CDCl_3_, *δ*/ppm): ^1^H (500 MHz) 1.49–1.36, 1.36–1.23 (2 br m, 72H, C*H̲*_2_); ^13^C{^1^H} (126 MHz) 30.9 (d, *J*_CP_ = 9.7 Hz, *C̲*H_2_), 28.7 (s, 2 × *C̲*H_2_), 28.6 (s, *C̲*H_2_), 27.0–26.5 (br s, *C̲*H_2_), 25.5 (d, *J*_CP_ = 10.6 Hz, *C̲*H_2_); ^31^P{^1^H} (202 MHz) −29.8 to −36.9 (br s) and (from Fig. 2b) −30.1/−40.4 (s/s, 213 K), −29.4/−38.0 (br s/br s, 253 K), −29.2/−36.5 (br s/br s, 273 K), −32.2 (br s, 298 K), −32.3 (br s, 323 K).

### Epimerization of 3c; *in*,*out*-3c·2BH_3_ and (*in*,*in*/*out*,*out*)-3c·2BH_3_

A Schlenk flask was charged with (*in*,*in*/*out*,*out*)-3c (0.252 g, 0.387 mmol) and mesitylene (10 mL). The solution was stirred at 150 °C. After 40 h, a ^31^P{^1^H} NMR spectrum showed a 51 : 49 (*in*,*in*/*out*,*out*)-3c/*in*,*out*-3c mixture. The solvent was removed by oil pump vacuum, the residue dissolved in THF (15 mL), and Me_2_S·BH_3_ (2.0 M in THF; 1.3 mL, 2.6 mmol) added with stirring. After 2 d, the solvent was removed by oil pump vacuum. The residue was chromatographed on a silica column (4 × 46 cm) using hexanes/CH_2_Cl_2_ (1 : 0 to 0 : 1 v/v). The solvents were removed from the product fractions by oil pump vacuum. First eluted *in*,*out*-3c·2BH_3_ (colorless oil, 0.110 g, 0.162 mmol, 42%). Anal. calcd for C_42_H_90_B_2_P_2_ (678.73): C, 74.32; H, 13.37; found: C, 73.86; H, 13.49.

NMR (CDCl_3_, *δ*/ppm): ^1^H (500 MHz) 1.56–1.51 (m, 12H, C*H̲*_2_), 1.49–1.42 (m, 12H, C*H̲*_2_), 1.39–1.33 (m, 12H, C*H̲*_2_), 1.31–1.21 (m, 48H, C*H̲*_2_), 0.45 and 0.27 (br apparent d, 6H, B*H̲*_3_); ^13^C {^1^H} (126 MHz) 30.5 (d, *J*_CP_ = 11.3 Hz, *C̲*H_2_), 28.35 (s, *C̲*H_2_), 28.28 (s, *C̲*H_2_), 28.2 (s, *C̲*H_2_), 28.1 (s, *C̲*H_2_), 23.0 (d, *J*_CP_ = 34.3 Hz, *C̲*H_2_), 22.2 (d, *J*_CP_ = 1.9 Hz, *C̲*H_2_); ^31^P{^1^H} (202 MHz): 15.8–15.4 (br m).^31*b*^

Next eluted (*in*,*in*/*out*,*out*)-3c·2BH_3_ (colorless oil, 0.114 g, 0.168 mmol, 43%), which solidified to a white powder. The NMR data agreed with that from the independent synthesis above.

### *in*,*out*-P((CH_2_)_14_)_3_P (*in*,*out*-3c)

A Schlenk flask was charged with *in*,*out*-3c·2BH_3_ (0.048 g, 0.071 mmol) and pyrrolidine (3 mL). The mixture was refluxed (60 °C) for 11 d. The solvent was removed by oil pump vacuum. Toluene (5 mL) was added, and the suspension passed through a pad of silica gel on a Schlenk frit (1.5 × 1 cm). The filter cake was washed with toluene (3 × 4 mL). The solvent was removed from the filtrate by oil pump vacuum to give *in*,*out*-3c (0.026 g, 0.040 mmol, 56%) as a colorless oil. Anal. calcd for C_42_H_84_P_2_ (651.06): C, 77.48; H, 13.00; found: C, 77.66; H, 13.09.

NMR (CDCl_3_, *δ*/ppm): ^1^H (500 MHz) 1.45–1.35, 1.32–1.23 (2 br m, 84H, C*H̲*_2_); ^13^C{^1^H} (126 MHz) 30.9 (d, *J*_CP_ = 10.3 Hz, *C̲*H_2_), 29.0 (s, 2 × *C̲*H_2_), 28.94 (s, *C̲*H_2_), 28.86 (s, *C̲*H_2_), 27.0 (d, *J*_CP_ = 11.5 Hz, *C̲*H_2_), 25.6 (d, *J*_CP_ = 11.8 Hz, *C̲*H_2_); ^31^P{^1^H} (202 MHz) −31.3 (s).

NMR (CDCl_2_F,^45^*δ*/ppm, 263 K): ^1^H (500 MHz) 1.46–1.25 (br m, 84H, C*H̲*_2_); ^13^C{^1^H} (126 MHz) 31.0 (d, *J*_CP_ = 10.3 Hz, *C̲*H_2_), 29.0 (s, 2 × *C̲*H_2_), 28.98 (s, *C̲*H_2_), 28.90 (s, *C̲*H_2_), 26.8 (d, *J*_CP_ = 10.9 Hz, *C̲*H_2_), 25.5 (d, *J*_CP_ = 11.5 Hz, *C̲*H_2_); ^31^P{^1^H} (202 MHz) −32.5 (s).

^31^P{^1^H} NMR (CH_2_Cl_2_, *δ*/ppm, 202 MHz selected data from Fig. 2a) −32.3/−37.7 (br s/br s, 183 K), −32.4/−37.4 (br s/br s, 193 K), −34.5 (br s, 208 K), −34.1 (br s, 223 K).

### Epimerization of 3e; *in*,*out*-3e·2BH_3_ and (*in*,*in*/*out*,*out*)-3e·2BH_3_

A Schlenk flask was charged with (*in*,*in*/*out*,*out*)-3e (0.1934 g, 0.2360 mmol) and mesitylene (10 mL). The solution was stirred at 150 °C. After 30 h, a ^31^P{^1^H} NMR spectrum showed a 51 : 49 (*in*,*in*/*out*,*out*)-3e/*in*,*out*-3e mixture. The solvent was removed by oil pump vacuum, the residue dissolved in THF (15 mL), and Me_2_S·BH_3_ (2.0 M in THF; 0.24 mL, 0.48 mmol) added with stirring. After 24 h, the solvent was removed by oil pump vacuum. The residue was chromatographed on a silica column (4 × 46 cm) using hexanes/CH_2_Cl_2_ (1 : 3 v/v). The solvents were removed from the product fractions by oil pump vacuum. First eluted *in*,*out*-3e·2BH_3_ (colorless oil, 0.0310 g, 0.0366 mmol, 16%). Anal. calcd for C_54_H_114_B_2_P_2_ (847.07): C, 76.57; H, 13.57; found: C, 76.53; H, 13.45.

NMR (CDCl_3_, *δ*/ppm): ^1^H (500 MHz) 1.59–1.52 (m, 12H, C*H̲*_2_), 1.52–1.44 (m, 12H, C*H̲*_2_), 1.41–1.34 (m, 12H, C*H̲*_2_), 1.34–1.22 (m, 72H, C*H̲*_2_), 0.49 and 0.29 (br apparent d, 6H, B*H̲*_3_); ^13^C{^1^H} (126 MHz) 31.0 (d, *J*_CP_ = 11.9 Hz, *C̲*H_2_), 29.4 (s, *C̲*H_2_), 29.31 (s, *C̲*H_2_), 29.28 (s, *C̲*H_2_), 29.27 (s, *C̲*H_2_), 29.0 (s, *C̲*H_2_), 28.8 (s, *C̲*H_2_), 23.1 (d, *J*_CP_ = 34.5 Hz, *C̲*H_2_), 22.5 (d, *J*_CP_ = 2.4 Hz, *C̲*H_2_); ^31^P{^1^H} (202 MHz): 15.1–13.9 (br s).^31*b*^

Next eluted (*in*,*in*/*out*,*out*)-3e·2BH_3_ (colorless oil, 0.0464 g, 0.0548 mmol, 23%), which solidified (6–8 h) to a white powder. The NMR data agreed with that from the independent synthesis above.

### *in*,*out*-P((CH_2_)_18_)_3_P (*in*,*out*-3e)

A Schlenk flask was charged with *in*,*out*-3e·2BH_3_ (0.031 g, 0.0366 mmol) and pyrrolidine (2 mL). The mixture was refluxed (60 °C) for 3 d. The solvent was removed by oil pump vacuum. Toluene (5 mL) was added, and the suspension passed through a pad of silica gel on a Schlenk frit (1.5 × 2 cm). The filter cake was washed with toluene (20 mL). The solvent was removed from the filtrate by oil pump vacuum to give *in*,*out*-3e (0.0197 g, 0.024 mmol, 66%) as a colorless oil. Anal. calcd for C_54_H_108_P_2_ (819.38): C, 79.15; H, 13.28; found: C, 79.02; H, 13.29.

NMR (CDCl_3_, *δ*/ppm): ^1^H (500 MHz) 1.46–1.33, 1.32–1.22 (2 br m, 108H, C*H̲*_2_); ^13^C{^1^H} (126 MHz) 31.2 (d, *J*_CP_ = 10.5 Hz, *C̲*H_2_), 29.43 (s, *C̲*H_2_), 29,42 (s, *C̲*H_2_), 29.39 (s, *C̲*H_2_), 29.38 (s, *C̲*H_2_), 29.3 (s, *C̲*H_2_), 29.1 (s, *C̲*H_2_), 27.2 (d, *J*_CP_ = 11.9 Hz, *C̲*H_2_), 25.8 (d, *J*_CP_ = 12.0 Hz, *C̲*H_2_); ^31^P{^1^H} (202 MHz) −31.5 (s).

### Conversion of (*in*,*in*/*out*,*out*)-3b to *trans*-2b

A J. Young NMR tube was charged with (*in*,*in*/*out*,*out*)-3b (0.0110 g, 0.0194 mmol), PtCl_2_ (0.0054 g, 0.020 mmol), and C_6_D_6_ (0.6 mL) in a glove box. The mixture was kept at 55 °C for 24 h and chromatographed (SiO_2_ column, 0.7 × 3 cm, 5 : 1 v/v hexanes/CH_2_Cl_2_). The solvent was removed from the product fractions by rotary evaporation to give *trans*-2b (0.0084 g, 0.0101 mmol, 52%) as a yellow powder, mp 160–161 °C. Anal. calcd for C_36_H_72_P_2_Cl_2_Pt (832.89): C, 51.91; H, 8.71; found: C, 53.25; H, 8.98.^46^

NMR (C_6_D_6_, *δ*/ppm): ^1^H (500 MHz) 1.92–1.82, (br m, 12H, PCH_2_C*H̲*_2_), 1.72–1.62 (br m, 12H, PC*H̲*_2_), 1.55–1.41 (br m, 48H, remaining C*H̲*_2_); ^13^C{^1^H} (126 MHz) 30.2 (virtual t, *J*_CP_ = 6.9 Hz, PCH_2_CH_2_*C̲*H_2_), 27.9 (s, *C̲*H_2_), 27.8 (s, *C̲*H_2_), 27.0 (s, *C̲*H_2_), 25.4 (virtual t, *J*_CP_ = 16.3 Hz, P*C̲*H_2_), 24.3 (s, P CH_2_*C̲*H_2_); ^31^P{^1^H} (202 MHz) 9.3 (s, *J*_PPt_ (satellite) = 2442 Hz).

### Conversion of (*in*,*in*/*out*,*out*)-3c to *trans*-2c

(A) A round bottom flask was charged with (*in*,*in*/*out*,*out*)-3c (0.0635 g, 0.097 mmol), PtCl_2_ (0.0306 g, 0.115 mmol), and CH_2_Cl_2_ (7 mL) in a glove box. The mixture was stirred for 24 h and chromatographed (SiO_2_ column, 1 × 5 cm, 4 : 1 v/v hexanes/CH_2_Cl_2_). The solvent was removed from the product fractions by rotary evaporation to give *trans*-2c (0.0825 g, 0.090 mmol, 93%) as a yellow powder.^47^ (B) A round bottom flask was charged with *in*,*in*/*out*,*out*-3c (0.0629 g, 0.096 mmol), PtCl_2_(NCCH_3_)_2_ (0.0400 g, 0.115 mmol), and THF (7 mL) in a glove box. The mixture was stirred for 6 h and worked up as in A to give *trans*-2c (0.0835 g, 0.091 mmol, 95%) as a yellow powder.^47^

### Conversion of (*in*,*in*/*out*,*out*)-3e to *trans*-2e

A round bottom flask was charged with (*in*,*in*/*out*,*out*)-3e (0.0788 g, 0.096 mmol), PtCl_2_ (0.0260 g, 0.098 mmol), and CH_2_Cl_2_ (10 mL) in a glove box. The mixture was stirred for 24 h and chromatographed (SiO_2_ column, 1 × 10 cm, 5 : 1 v/v hexanes/CH_2_Cl_2_). The solvent was removed from the product fractions by rotary evaporation to give *trans*-2e (0.0719 g, 0.0662 mmol, 69%) as a yellow powder.^47^

### Conversion of (*in*,*in*/*out*,*out*)-3c to *trans*-PtCl_2_(P((CH_2_)_14_)_3_P (*trans*-4c)

(A) A round bottom flask was charged with *in*,*in*/*out*,*out*-3c (0.0668 g, 0.102 mmol), PdCl_2_ (0.0216 g, 0.122 mmol), and CH_2_Cl_2_ (7 mL) in a glove box. The mixture was stirred for 24 h and chromatographed (SiO_2_ column, 1 × 5 cm, 4 : 1 v/v hexanes/CH_2_Cl_2_). The solvent was removed from the product fractions by rotary evaporation to give *trans*-4c (0.0795 g, 0.096 mmol, 94%) as a yellow powder.^45^ (B) A round bottom flask was charged with *in*,*in*/*out*,*out*-3c (0.0740 g, 0.113 mmol), PdCl_2_(NCCH_3_)_2_ (0.0311 g, 0.120 mmol), and THF (7 mL) in a glove box. The mixture was stirred for 6 h and worked up as in A to give *trans*-4c (0.0886 g, 0.107 mmol, 95%) as a yellow powder.^47^

## Data availability

Additional experimental data are deposited in the ESI† associated with this publication and any further data are available upon request.

## Author contributions

All authors except J.A.G. contributed to the experimental work. All authors participated in data analysis. Overall supervision and funding acquisition was carried out by J.A.G. The manuscript was written by J.A.G., Y.Z., and M.S. with contributions by all authors.

## Conflicts of interest

The authors declare no competing financial interest.

## Supplementary Material

SC-013-D2SC04724A-s001

SC-013-D2SC04724A-s002

SC-013-D2SC04724A-s003
