# Supplementary material for: Syntheses, homeomorphic and configurational isomerizations, and structures of macrocyclic aliphatic dibridgehead diphosphines; molecules that turn themselves inside out
Source: Chem Sci. 2022 Oct 20;13(45):13368–86. doi: 10.1039/d2sc04724a (PMC9682899; doi:10.1039/d2sc04724a)
Supplement: SC-013-D2SC04724A-s001 [file SC-013-D2SC04724A-s001.pdf]

Electronic Supplementary Material (ESI) for Chemical Science.
This journal is © The Royal Society of Chemistry 2022

Syntheses, Homeomorphic and Configurational Isomerizations, and Structures of Macrocyclic Aliphatic Dibridgehead Diphosphines; Molecules that Turn Themselves Inside Out

Yun Zhu, Michael Stollenz, Samuel R. Zarcone, Sugam Kharel, Hemant Joshi, Nattamai Bhuvanesh, Joseph H. Reibenspies, and John A. Gladysz*

Department of Chemistry, Texas A&M University, PO Box 30012, College Station, Texas 77842-3012, USA

■ EXPERIMENTAL SECTION (continued)

General. All diphosphines were manipulated under inert atmospheres. Other chemicals were treated as follows: hexanes, benzene, and THF, distilled from Na/benzophenone or Na; CH_2Cl_2 and toluene, purified by a Glass Contour system; NaC≡CH, isolated by filtration of a xylene suspension (18 wt%, Acros), washed with toluene under argon, and dried in vacuo (18 h); PhC≡CH (97%, TCI) and mesitylene (97%, TCI), degassed by three freeze-pump-thaw cycles; $CDCl_3$, C_6D_6, toluene-d_8 (3 × Cambridge Isotopes), $Me_2S{\cdot}BH_3$ (2.0 M in THF, Acros), pyrrolidine (99%, Alfa Aesar), KCN (97%, Alfa Aesar), ethyl acetate (99.5%, Sigma-Aldrich), PtO_2 (99%, ChemScene), Grubbs' catalyst (97%, Sigma-Aldrich), *n*-BuLi (2.5 or 1.6 M in hexanes, Acros), celite (EMD), and neutral alumina (Brockmann I, for chromatography, 40–300 μm, 60A, Acros), used as received; $CDCl_2F$, prepared by a literature procedure[s1] and distilled from CaH_2.

NMR spectra were recorded on a Varian NMRS 500 spectrometer at ambient probe temperature unless noted and referenced as follows (δ/ppm): ^1H, residual internal $CHCl_3$ (7.26), C_6D_5H (7.16), toluene-d_7 (7.09), or acetone-d_5 (2.05); ^{13}C, internal $CDCl_3$ (77.16), C_6D_6 (128.06), toluene-d_8 (137.48), or acetone-d_6 (29.84); ^{31}P, external 85% H_3PO_4 (0.00). IR spectra were obtained on a Shimadzu IRAffinity-1 spectrometer with a Pike MIRacle ATR system (diamond/ZnSe crystal). Melting points were recorded using a Stanford Research Systems MPA100 (OptiMelt) automated apparatus. Microanalyses were conducted by Atlantic Microlab.

***cis*-$\overline{PtCl_2(P((CH_2)_{12})_3P)}$ (*cis*-2b).** Higher yield procedure. A three-neck flask fitted with a condenser was charged with *cis*-**1b** (0.7523 g, 0.8258 mmol)[s2] and CH_2Cl_2 (800 mL). A solution of Grubbs' catalyst (0.849 g, 0.103 mmol) in CH_2Cl_2 (50 mL) was added dropwise at 0 °C over 40 min with stirring. The mixture was refluxed. After 18 h, the sample was cooled to room temperature and another charge of Grubbs' catalyst added (0.0854 g, 0.1038 mmol). The mixture was refluxed. After 24 h, the solvent was removed by rotary evaporation. The residue was chromatographed on neutral alumina (4 × 13 cm column) with CH_2Cl_2 and ethyl acetate. The solvent was removed by rotary evaporation from the ethyl acetate eluates. The light brown/grey solid was transferred to a Fisher-Potter bottle that was charged with PtO_2 (0.0427 g, 0.1880 mmol),

CH_2Cl_2 (ca. 10 mL), and H_2 (5 atm). The mixture was stirred at 45 °C for 168 h and after depressurization filtered through celite. The filtrate was concentrated and chromatographed on neutral alumina (2.5 × 15 cm column) with CH_2Cl_2 and ethyl acetate. The solvent was removed by rotary evaporation from the ethyl acetate eluates to give *cis*-**2b** (0.1792 g, 0.2152 mmol, 25%) as a white solid. NMR data agreed with those published earlier.[s3]

LiC≡CPh.[s4] A Schlenk flask was charged with PhC≡CH (0.998 g, 9.58 mmol) and hexanes (40 mL). The solution was cooled to –78 °C and *n*-BuLi (2.5 M in hexanes; 6.0 mL, 9.6 mmol) was added dropwise with stirring. A white precipitate formed. The mixture was slowly warmed to room temperature overnight, and concentrated by oil pump vacuum. The precipitate was isolated by filtration, washed with hexanes (4 × 10 mL), and dried by oil pump vacuum to give LiC≡CPh as a white powder (1.010 g, 9.35 mmol, 98%).

$Li_2[Pt(C{\equiv}CPh)_4]\cdot 4THF$.[s5] A Schlenk flask was charged with $PtCl_2$(tetrahydrothiophene)$_2$ (0.161 g, 0.364 mmol)[s6] and THF (15 mL). The solution was cooled to –78 °C and a –78 °C solution of LiC≡CPh (0.158 g, 1.46 mmol) in THF (15 mL) added with stirring. The mixture was slowly warmed to room temperature, and after 18 h concentrated to 15 mL. A white precipitate formed. The sample was warmed to dissolve the precipitate, and then kept at –35 °C overnight. The solvent was decanted from the microcrystalline residue, which was washed with THF (–78 °C, 2 × 5 mL) and dried by oil pump vacuum (2 h) to give $Li_2[Pt(C{\equiv}CPh)_4]\cdot 4THF$ as a white powder (0.199 g, 0.221 mmol, 61%).

NMR (acetone-d_6, δ/ppm): ^1H (500 MHz) 7.24-7.23 (m, 8H, *o*-Ph), 7.14-7.11 (m, 8H, *m*-Ph), 7.04-7.00 (m, 4H, *p*-Ph), 3.64-3.61 (m, 16H, OCH_2), 1.80-1.77 (m, 16H, $OCH_2C\underline{H_2}$); $^{13}C\{^1H\}$ (126 MHz) 132.9 (s, *o*-Ph), 131.8 (s, *i*-Ph), 129.4 (s, *m*-Ph), 126.0 (s, *p*-Ph), 111.6 (br s, PtC≡), 107.3 (s, $^2J_{PtC}$ = 294 Hz,[s7] ≡CPh), 69.1 (s, OCH_2), 27.1 (s, $OCH_2\underline{C}H_2$). IR (cm^{-1}, powder film) $\nu_{C\equiv C}$ 2089 (s).

(*in,in*/*out,out*)-$P((CH_2)_{14})_3P$ ((*in,in*/*out,out*)-3c) using NaC≡CH. A Schlenk flask was charged with a suspension of NaC≡CH (1.368 g, 28.49 mmol) in THF (30 mL). A solution of *trans*-**2c** (0.523 g, 0.570 mmol)[s8] in THF (20 mL) was added with stirring. After 3 d, the mixture

was filtered. The filter cake was washed with THF (3 × 5 mL). The solvent was removed from the filtrate by oil pump vacuum, and hexanes (50 mL) added. The suspension was filtered through celite and the filter cake washed with hexanes (3 × 5 mL). The solvents were removed from the filtrate by oil pump vacuum (15 h) to give (*in,in*/*out,out*)-**3c** as a white powder (0.339 g, 0.521 mmol, 91%).[s8]

(*in,in*/*out,out*)-3c using LiC≡CPh. A Schlenk flask was charged with *trans*-**2c** (0.0706 g, 0.0770 mmol)[s8] and LiC≡CPh (0.0403 g, 0.373 mmol) in a glove box, and THF (3 mL) added with stirring. After 4 d, a white precipitate had formed. The solvent was removed by oil pump vacuum, and benzene added. The mixture was shaken. After 5 min, the clear supernatant was transferred by cannula to a second Schlenk flask. The solvent was removed by heating and oil pump vacuum to give (*in,in*/*out,out*)-**3c** as a yellow solid (0.0431 g, 0.0662 mmol, 86%).

The residue from the first Schlenk flask was washed with hexanes (12 mL), recrystallized from THF, and dried by oil pump vacuum to give $Li_2[Pt(C{\equiv}CPh)_4]\cdot 4THF$ (see independent synthesis above) as a white powder (0.0241 g, 0.0267 mmol, 35%).

NMR (acetone-d_6, δ/ppm): ^{1}H (500 MHz) 7.24-7.22 (m, 8H, *o*-Ph), 7.14-7.10 (m, 8H, *m*-Ph), 7.04-7.00 (m, 4H, *p*-Ph), 3.64-3.61 (m, 16H, OCH_2), 1.80-1.77 (m, 16H, $OCH_2C\underline{H}_2$); $^{13}C\{^1H\}$ (101 MHz) 133.0 (s, *o*-Ph), 131.7 (s, *i*-Ph), 129.4 (s, *m*-Ph), 126.0 (s, *p*-Ph), 111.2 (br s, PtC≡), 107.3 (s, $^2J_{PtC}$ = 293 Hz,[s7] PhC≡), 69.1 (s, OCH_2), 27.1 (s, $OCH_2\underline{C}H_2$). IR (cm^{-1}, powder film): $\nu_{C\equiv C}$ 2085 (s).

Crystallography.

A. *out,out*-3b. A hexanes solution of (*in,in*/*out,out*)-**3b** was allowed to slowly concentrate under argon at –38 °C. After 7 d, clear plates were collected and data obtained per Table s1. Cell parameters were derived from 45 frames using a 1° scan and refined with 17631 reflections. Integrated intensity information for each reflection was obtained by reduction of the data frames with the program APEX3.[s9] Lorentz, polarization, crystal decay, and adsorption corrections were applied, the last with the program SADABS.[s10] The space group was determined from systematic reflection conditions and statistical tests. Non-hydrogen atoms were refined with anisotropic

thermal parameters. Hydrogen atoms were fixed in idealized positions using a riding model. The absence of additional symmetry or voids was confirmed using PLATON (ADDSYM).[s11] The structure was refined (weighted least squares refinement on F^2) to convergence.[s12]

B. *out,out*-3c. A saturated hexanes solution of (*in,in*/*out,out*)-**3c** was kept at 4 °C. Hexagonal plates were collected and data obtained per Table s1. Integrated intensities for each reflection were obtained by reduction of the data frames with the program SAINT.[s13] Cell parameters were obtained and refined with 14394 (1047 independent) reflections.[s14] Lorentz, polarization, and adsorption corrections were applied, the last with the program SADABS.[s10] The space group was determined from systematic reflection conditions and statistical tests. The structure was solved by direct methods and refined (weighted least squares refinement on F^2) using SHELXL-97.[s12] Non-hydrogen atoms were refined with anisotropic thermal parameters. The hydrogen atoms were placed in idealized positions, and refined using a riding model.

C. *out,out*-3e. A CH_2Cl_2 solution of (*in,in*/*out,out*)-**3e** was allowed to slowly concentrate under a N_2 atmosphere at −35 °C. After 18 d, yellows plates were collected and data obtained per Table s1. Cell parameters were obtained from 45 frames using a 1° scan and refined with 46796 reflections. Integrated intensity information for each reflection was obtained by reduction of the data frames with the program APEX3.[s9] Lorentz, polarization, and adsorption corrections were applied, the last with the program SADABS.[s10] The space group was determined from systematic reflection conditions and statistical tests. Non-hydrogen atoms were refined with anisotropic thermal parameters. Hydrogen atoms were fixed in idealized positions using a riding model. Elongated or abnormal thermal ellipsoids for all carbon atoms of two methylene chains (related by a C_2 symmetry axis passing through the midpoint of the third chain) indicated possible disorder, which was modeled between two positions with an occupancy ratio of 0.77:0.23. The absence of additional symmetry or voids was confirmed using PLATON (ADDSYM).[s11] The structure was refined (weighted least squares refinement on F^2) to convergence.[s12]

D. *out,out*-3c·2BH$_3$·(C$_5$H$_9$CH$_3$) and *out,out*-3c·2BH$_3$·(C$_6$H$_{11}$CH$_3$). Hexanes and methylcyclohexane solutions of (*in,in*/*out,out*)-**3c**·$2BH_3$ were allowed to slowly concentrate at

room temperature. Data were obtained on the resulting crystals per Table s1. Integrated intensities for each reflection were acquired by reduction of the data frames with the program SAINT.[s13] Cell parameters were obtained from 3960 frames using ω and φ scans and refined with 15936 and 14070 reflections, respectively.[s14] Lorentz, polarization, and adsorption corrections were applied, the last with the program SADABS.[s10] For *out,out*-**3c**·$2BH_3$·($C_5H_9CH_3$), the methylene chains C1 to C14 and C15 to C28 exhibited elongated thermal ellipsoids, suggesting disorder that was modeled with occupancy ratios of 0.81/0.19 (C1 to C14) and 0.52/0.48 (C15 to C28).[s15] Restraints were used to keep the bond distances and thermal ellipsoids meaningful. For *out,out*-**2**·$2BH_3$·($C_6H_{11}CH_3$), the thermal parameters of the phosphorus atoms, the boron atoms, and one methylene chain (C1 to C14) were very well defined. The ellipsoids of the other methylene chains (C15 to C28 and C29 to C42) were elongated, indicating possible disorder. At this point the R factor was 12.9%. The restraints SIMU and DELU were applied to the latter two chains, resulting in a final R factor of 13.4%. Attempts to model the disorder increased the numbers of parameters and restraints, as well as the R factor (>16%).

E. *out,out*-3e·$2BH_3$. A mesitylene solution of (*in,in*/*out,out*)-**3e**·$2BH_3$ was allowed to slowly concentrate. After 20 d, colorless blocks were collected and data collected and refined by protocols analogous to those in C. Three independent molecules were present in the unit cell.

Rate and 2D NMR Measurements. A (Figure s8)**.** A J. Young valve NMR tube was charged with (*in,in*/*out,out*)-**3c** (0.050 M mesitylene solution, 0.56 mL) and immersed in a 150 ± 1 °C oil bath (hot plate thermostat). At various intervals, the tube was cooled (ice water) and $^{31}P\{^1H\}$ NMR spectra recorded. The (*in,in*/*out,out*)-**3c**/*in,out*-**3c** ratio was determined by integration. The tube was returned to the bath. The rate constant was calculated from the slope of a ln([(*in,in*/*out,out*)-**3c**]–[(*in,in*/*out,out*)-**3c**]$_{eq}$) vs. t plot (–slope = $k_{obs} = k_1 + k_{-1}$; k_1 is the forward direction for the experiment).[s16] K_{eq} and k_1 values of 49:51 (*in,out*/(*in,in*/*out,out)*) and $1.47 \times 10^{-5}\ s^{-1}$ were determined. The $\Delta G^{\ddagger}{}_{423\ K}$ value (34.4 kcal/mol) was calculated from the Eyring equation ($\ln(k_1) = \ln(kT/h) - \Delta G^{\ddagger}/RT$). **B** (Figure 2)**.** The T_c values in Figure 2 were extrapolated

(300 K and 200 K) and the $\Delta G^{\ddagger}_{Tc}$ values (degenerate *in,out*-**3b**,**c** ⇆ *out,in*-**3b**,**c**) calculated using the Gutowsky-Holm and Eyring equations.[s17] **C** (Figure s2). The $^{31}P\{^{1}H\}$ EXSY NMR spectrum was recorded on at 193 K in toluene-d_8. Two-dimensional data were collected using the pulse sequence PNOSE (p_1: (x, –x) p_2: $(x)_8$ $(-x)_8$ p_3: $(x)_2$, $(-x)_2$, (y_2), $(-y_2)$).[s18]

Mass Spectrometry (*m*/*z*).

(*in,in*/*out,out*)-**3c** (ESI+): 651.6 ($[M+H]^+$, 58%)

in,out-**3c** (ESI+): 651.5 ($[M+H]^+$, 56%).

(*in,in*/*out,out*)-**3c**·$2BH_3$ ((MALDI+): 651.6 ($[M-2BH_3+H]^+$, 100%).

■ REFERENCES

(s1) Siegel, J. S.; Anet, F. A. L. Dichlorofluoromethane-d: a versatile solvent for VT-NMR experiments. *J. Org. Chem.* **1988**, *53*, 2629-2630.

(s2) Nawara-Hultzsch, A. J.; Skopek, K.; Shima, T.; Barbasiewicz, M.; Hess, G. D.; Skaper, D.; Gladysz, J. A. Syntheses and Palladium, Platinum, and Borane Adducts of Symmetrical Trialkylphosphines with Three Terminal Vinyl Groups, $P((CH_2)_mCH=CH_2)_3$. *Z. Naturforsch. B: Anorg. Chem., Org. Chem.* **2010**, *65*, 414-424.

(s3) Joshi, H.; Kharel, S.; Ehnbom, A.; Skopek, K.; Hess, G. D.; Fiedler, T.; Hampel, F.; Bhuvanesh, N.; Gladysz, J. A. Three Fold Intramolecular Ring Closing Alkene Metatheses of Square Planar Complexes with *cis* Phosphorus Donor Ligands $P(X(CH_2)_mCH=CH_2)_3$ (X/*m* = –/5-10, O/3-5); Syntheses, Structures, and Thermal Properties of Macrocyclic Dibridgehead Diphosphorus Complexes. *J. Am. Chem. Soc.* **2018**, *140*, 8463-8478

(s4) (a) Bauer, W.; Seebach, D. Bestimmung des Aggregationsgrads lithiumorganischer Verbindungen durch Kyroskope in Tetrahydrofuran. *Helv. Chim. Acta* **1984**, *67*, 1972. (b) Hässig, R.; Seebach, D. Bestimmung der Struktur von Phenyläthinyllithium in Lösung mittels Tieftemperatur-NMR-Spektroskope. *Helv. Chim. Acta* **1983**, *66*, 2269.

(s5) Falvello, L. R.; Fornies, J.; Gómez, J.; Lalinde, E.; Martín, A.; Moreno, M. T.; Sacristán, J. Reactivity of Alkynyl Platinum Complexes towards PPh_2H and $PPh_2(O)H$; Unexpected Formation of Alkynyl Tetralithium Diplatinum Compounds Stabilized by μ3-(κ^3*P*,*O*,*O*-PPh_2O^-) Ligands. *Chem. Eur. J.* **1999**, *5*, 474-491.

(s6) Turley, P. C.; Haake, P. Proton Magnetic Resonance Spectra of Platinum(II) Com-

plexes. II. *cis*- and *trans*-Bis(dialkyl suflide)dichloroplatinum(II) Complexes. Mechanism of Inversion at Sulfur and Vicinal Platinum-Proton Couplings. *J. Am. Chem. Soc.* **1967**, *89*, 4617-4621.

(s7) This coupling represents a satellite (d, ^{195}Pt = 33.8%) and is not reflected in the peak multiplicity given.

(s8) Nawara-Hultzsch, A. J.; Stollenz, M.; Barbasiewicz, M.; Szafert, S.; Lis, T.; Hampel, F.; Bhuvanesh, N.; Gladysz, J. A. Gyroscope-Like Molecules Consisting of PdX_2/PtX_2 Rotators within Three-Spoke Dibridgehead Diphosphine Stators: Syntheses, Substitution Reactions, Structures, and Dynamic Properties. *Chem. Eur. J.* **2014**, *20*, 4617-4637.

(s9) *APEX3, Program for Data Collection on Area Detectors*. Bruker AXS Inc., Madison, WI 53711-5373 USA.

(s10) Sheldrick, G. M. *SADABS, Program for Absorption Correction for Data from Area Detector Frames.* Bruker AXS Inc., Madison, WI 53711-5373 USA.

(s11) Spek, A. L. Single-crystal structure validation with the program *PLATON*. *J. Appl. Cryst.* **2003**, *36*, 7-13.

(s12) (a) Sheldrick, G. M. SHELXT – Integrated space-group and crystal structure determination. *Acta Cryst.* **2015**, *A71*, 3-8. (b) Sheldrick, G. M. Crystal structure refinement with *SHELXL*. *Acta Cryst.* **2015**, *C71*, 3-8.

(s13) *SAINT (Version 7), Program for Data Integration from Area Detector Frames.* Bruker AXS Inc., Madison, WI 53711-5373 USA.

(s14) *FRAMBO v. 4.1.05, Program for Data Collection on Area Detectors*; Bruker AXS Inc., Madison, WI 53711-5373 USA.

(s15) For *out,out*-**3**·$2BH_3\cdot(C_5H_9CH_3)$, the numbering of carbon atoms C1-C42 has been changed from that in the CIF to be comparable with *out,out*-**3**·$2BH_3\cdot(C_6H_{11}CH_3)$.

(s16) Capellos, C.; Bielski, B. H. J. *Kinetic Systems*; Wiley: New York, 1972; Chapter 4 or 8.

(s17) Gutowsky, H. S.; Holm, C. H. Rate Processes and Nuclear Magnetic Resonance Spectra. II. Hindered Internal Rotation of Amides. *J. Chem. Phys.* **1956**, *25*, 1228.

(s18) Braun, S.; Kalinowski, O.-H.; Berger, S. *100 and More Basic NMR Experiments*, VCH, Weinheim, 1992, Chapter 10.

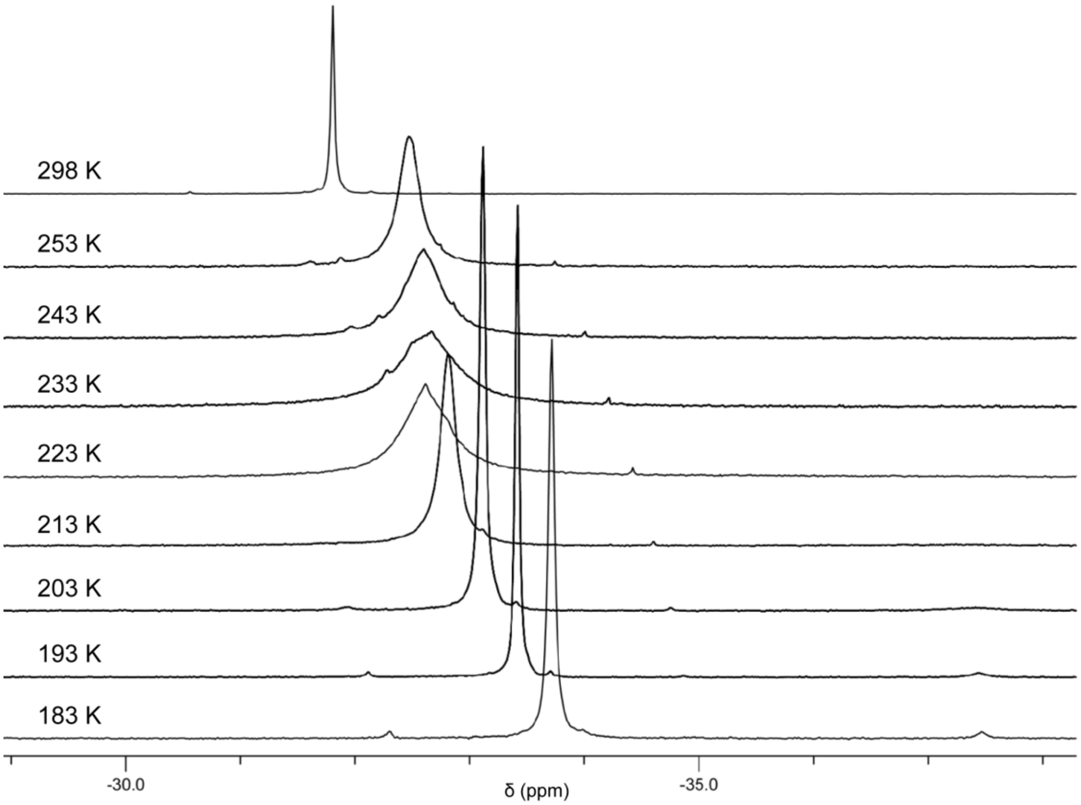

Figure s1. Variable-temperature $^{31}P\{^{1}H\}$ NMR spectra of (*in*,*in*/*out,out*)-**3c** in toluene-d_8 (162 MHz). Selected data (δ/ppm, major/minor): 183 K, –33.7/–37.5; 193 K, –33.4/–37.5; 203 K, –33.1; 213 K –32.8; 223 K –32.6; 253 K, –32.4; 298 K –31.8 s.

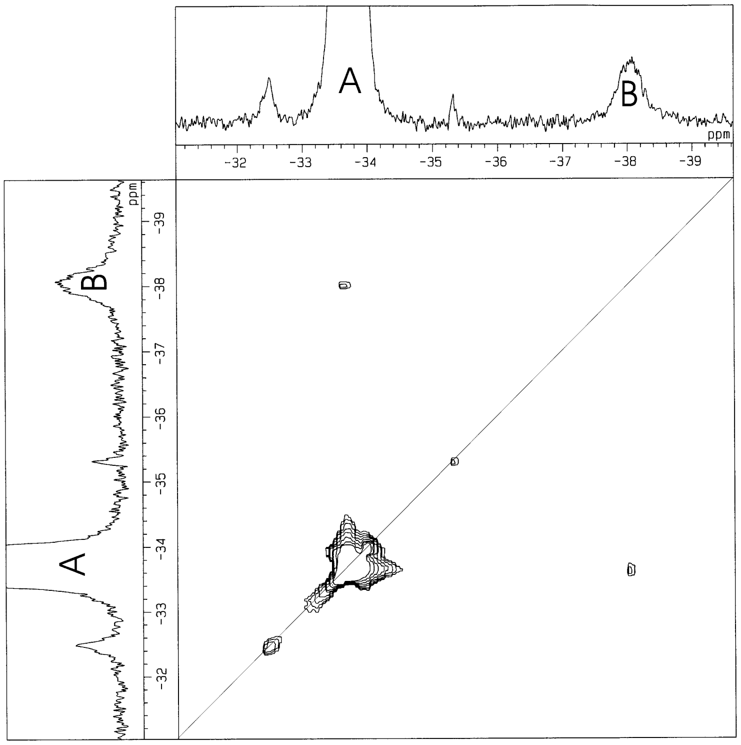

Figure s2. $^{31}P\{^{1}H\}$ EXSY NMR spectrum of (*in*,*in*/*out,out*)-**3c** at 193 K in toluene-d_8 (202 MHz), mixing time 0.8 s.

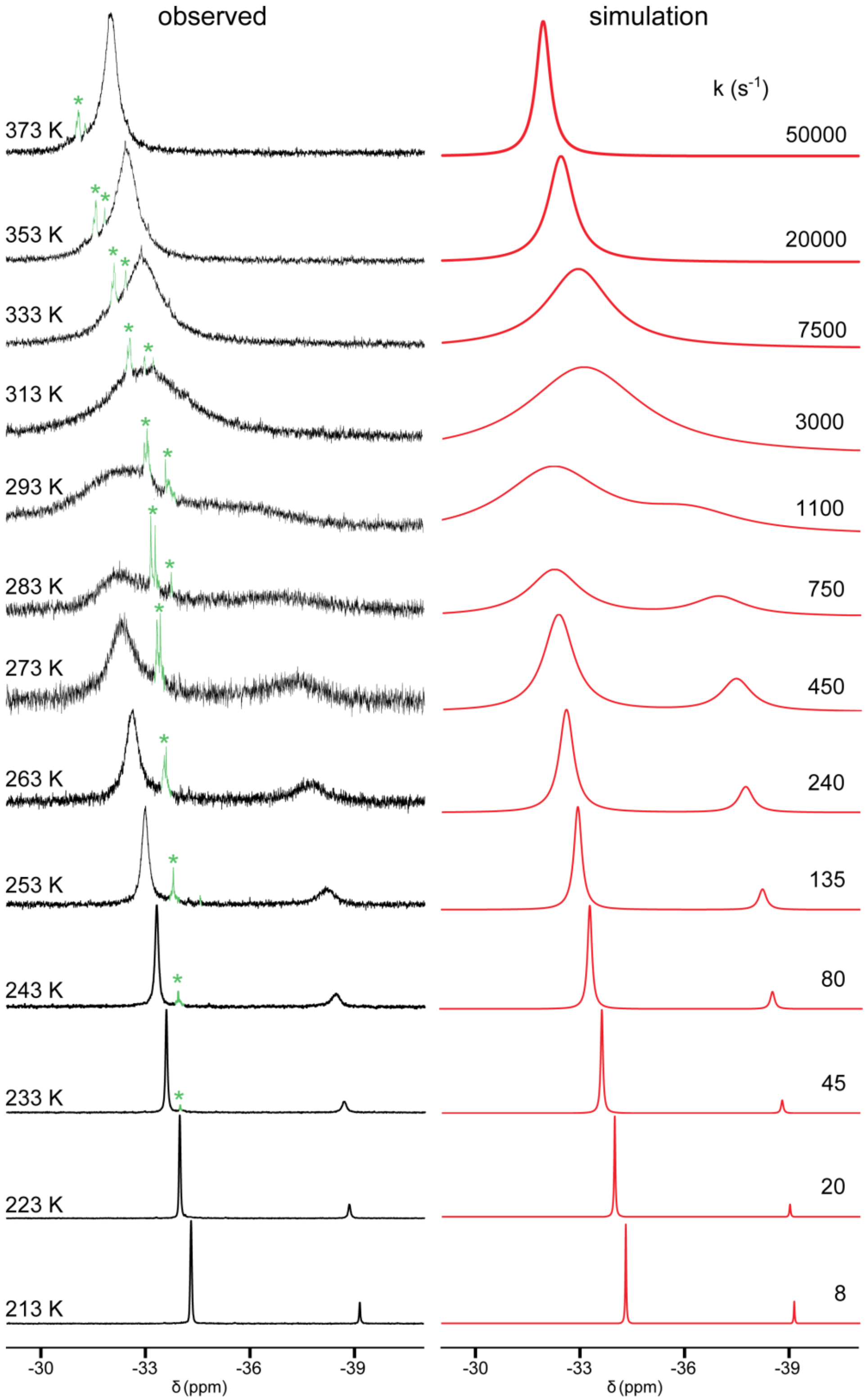

Figure s3. Variable-temperature $^{31}P\{^1H\}$ NMR spectra of (*in,in*/*out,out*)-**3b** in toluene-d_8 (left, 202 MHz) and simulated line shapes with rate constants (minor to major isomer); the asterisks * denotes unknown impurities. Selected data (δ/ppm, major/minor): 213 K, –34.3/–39.1; 223 K, –33.9/–39.0; 233 K, –33.6/–38.7; 263 K –32.6/–37.8, 283 K –32.1/–37.0; 333 K, –32.8; 373 K –31.9 (213 K, mesitylene: –34.6/–41.1; 213 K, THF: –33.5/–38.4).

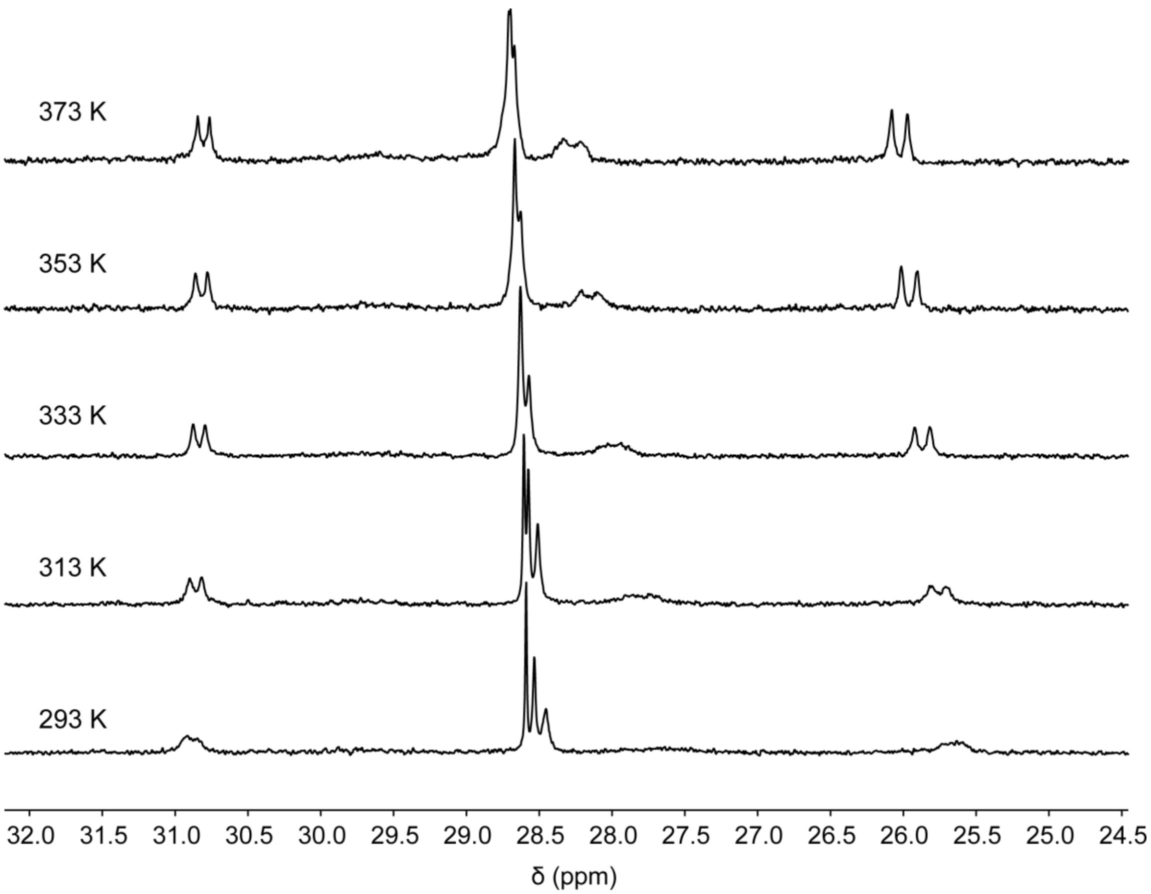

Figure s4. Variable-temperature $^{13}C\{^{1}H\}$ NMR spectra of *in,in*/*out,out*-**3b** in toluene-d_8 (126 MHz).

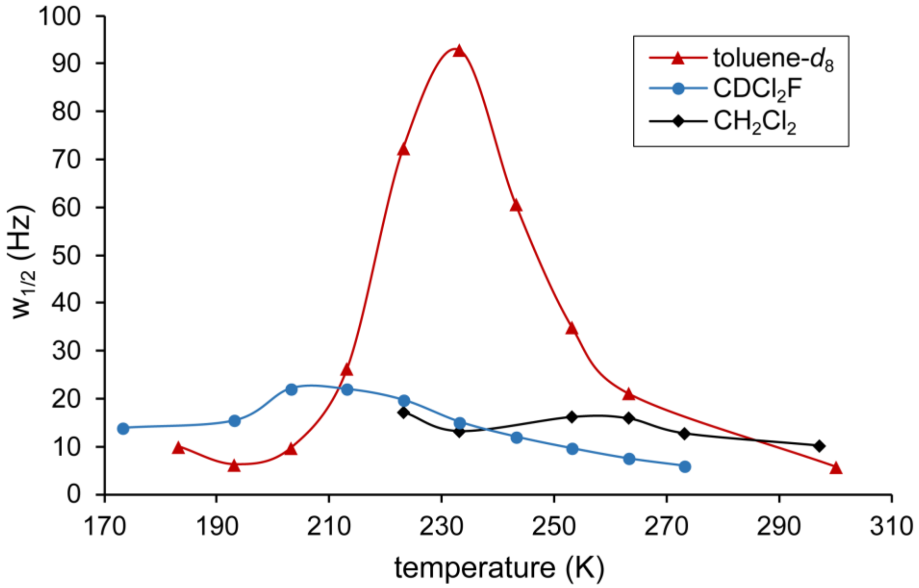

Figure s5. Peak widths ($w_{1/2}$) for the $^{31}P\{^{1}H\}$ NMR signal of (*in,in/out,out*)-**3c** as a function of solvent and temperature (162 or 202 MHz). Below T_c in toluene-d_8 (Figure s1), the peak width is for the major isomer (*in,in*-**3c**). Possible interpretations of the data in the chlorinated solvents include, *inter alia*, (a) that still lower temperatures are required for the decoalescence of the signals of *in,in*- and *out,out*-**3c**, or (b) that the K_{eq} has greatly increased from the 97:3 in toluene-d_8.

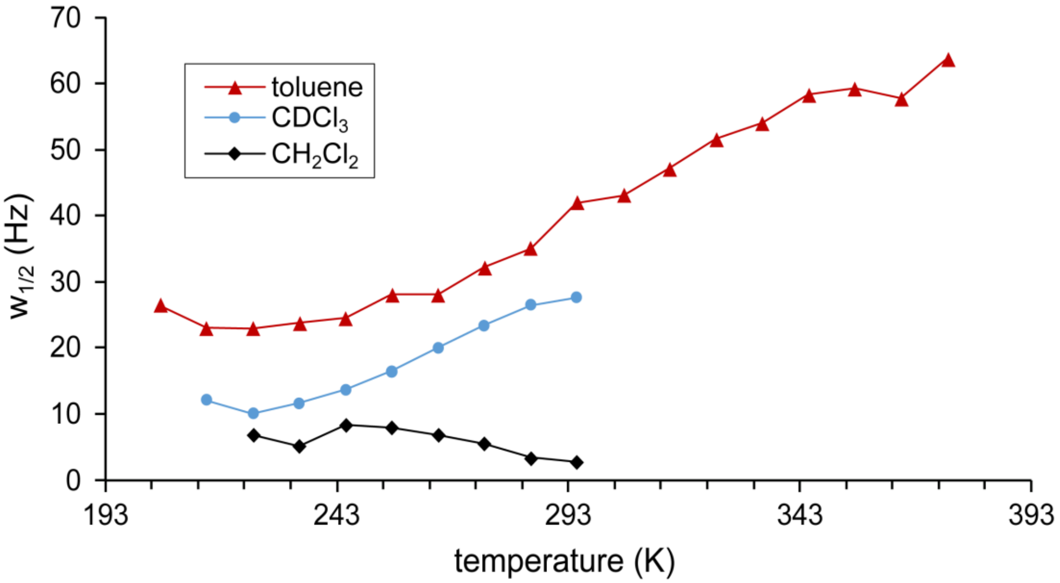

Figure s6. Peak widths ($w_{1/2}$) for the $^{31}P\{^1H\}$ NMR signal of (*in,in*/*out,out*)-**3e** as a function of solvent and temperature (202 MHz). One interpretation of these data is that the barriers for interconverting *in,in*- and *out,out*-**3e** are much lower than for the lower homologs.

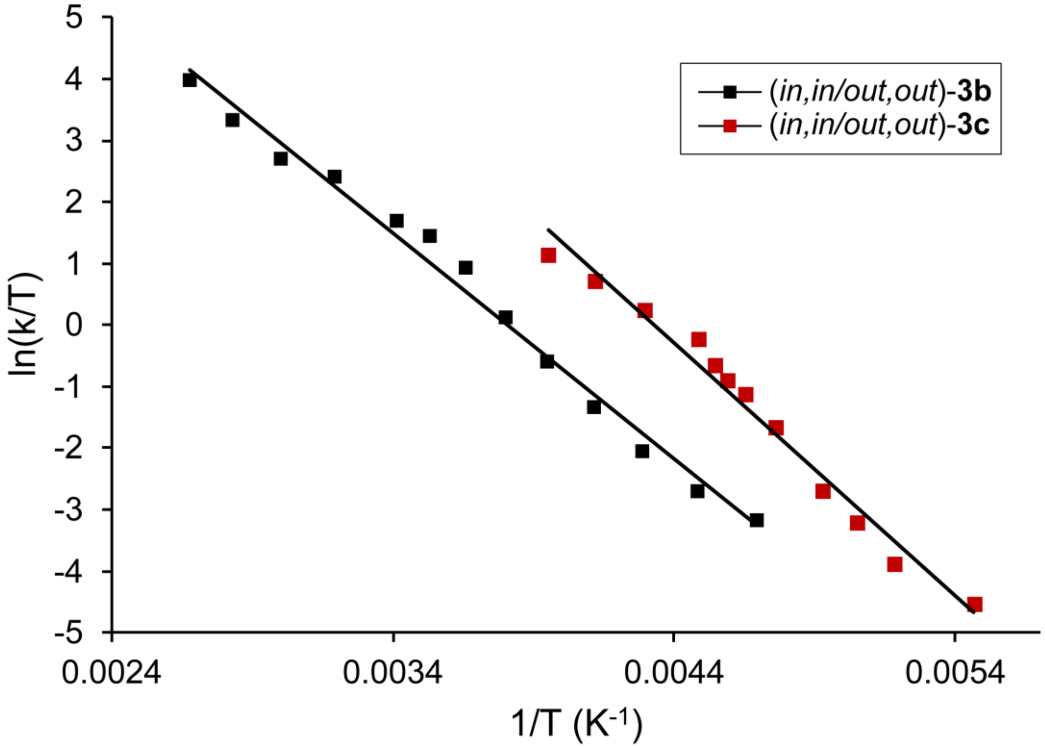

Figure s7. Eyring plots for the rate constants derived from Figures 1, s1, and s3 (minor to major isomers). Rate constants for *out,out*-**3c** to *in,in*-**3c** that are not displayed (k, s^{-1}): 183 K, 2; 193 K, 4; 203 K, 14; 213 K, 70; 223 K, 180; 233 K, 300; 243K 500; 253 K, 800.

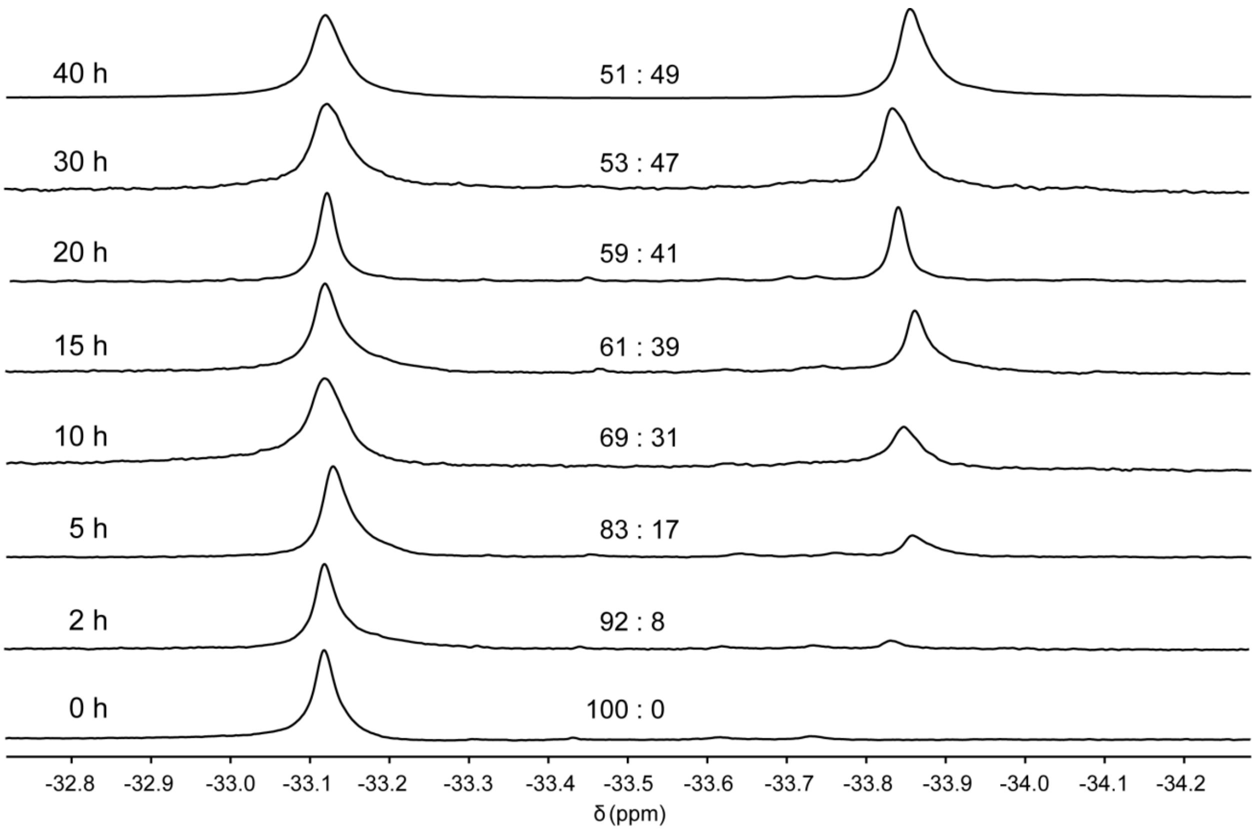

Figure s8. $^{31}P\{^{1}H\}$ NMR spectra (202 MHz, ambient probe temperature) of a mesitylene solution (*in,in*/*out,out*)-**3c** otherwise kept at 150 °C, showing the gradual epimerization to an equilibrium mixture with *in,out*-**3c**.

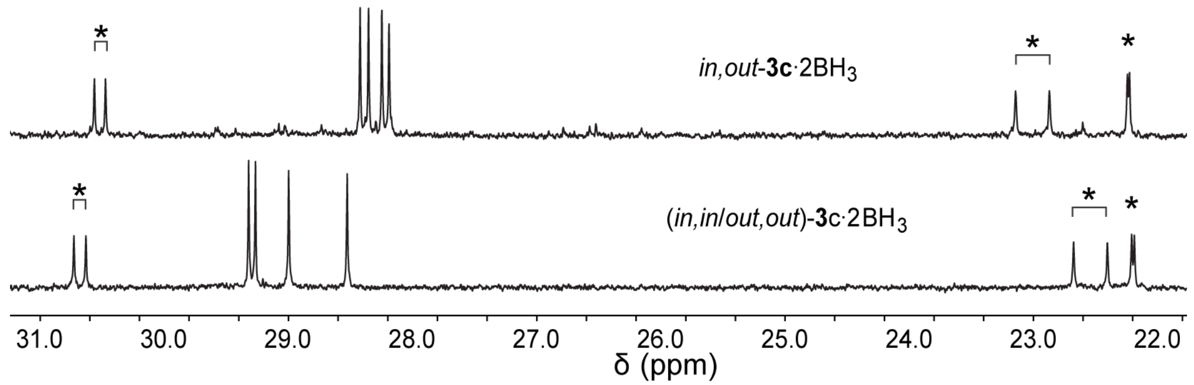

Figure s9. $^{13}C\{^{1}H\}$ NMR spectra of (*in,in*/*out,out*)-**3c**·$2BH_3$ and *in,out*-**3c**·$2BH_3$ in $CDCl_3$ (100 MHz). Doublets are marked with asterisks *.

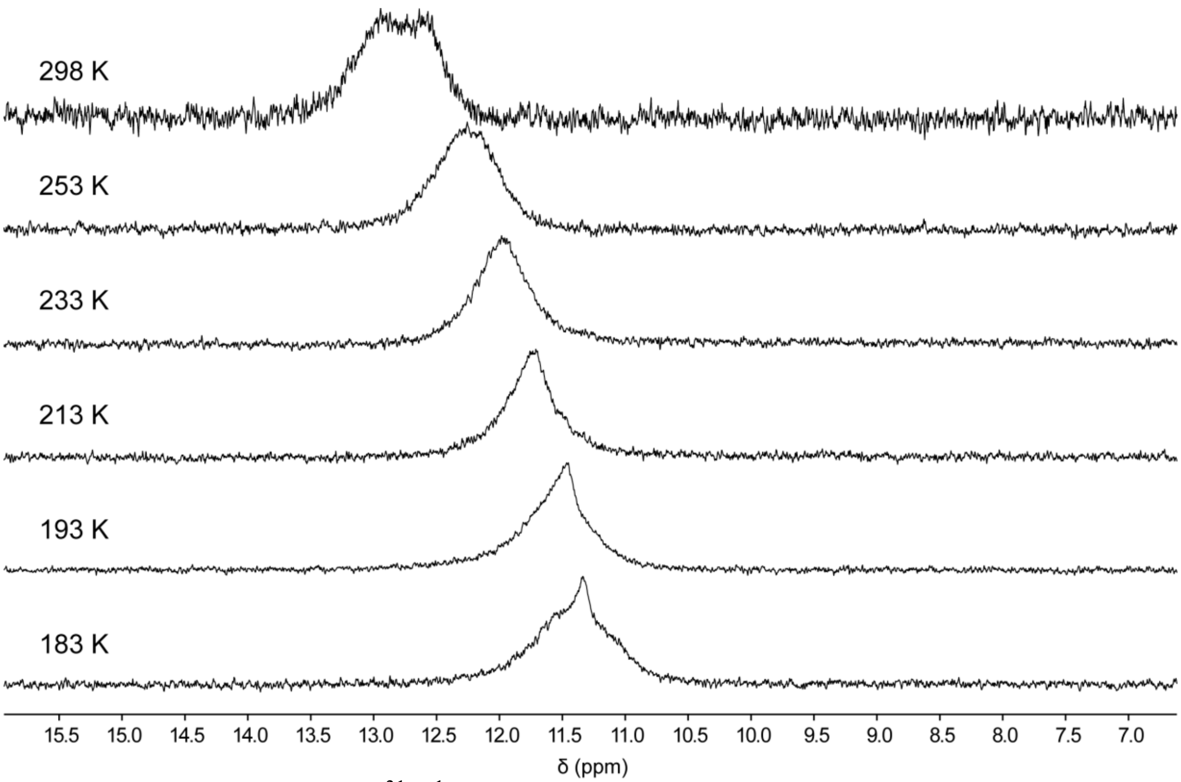

Figure s10. Variable-temperature $^{31}P\{^1H\}$ NMR spectra of (*in,in*/*out,out*)-**3c**·$2BH_3$ in CD_2Cl_2 (202 MHz).

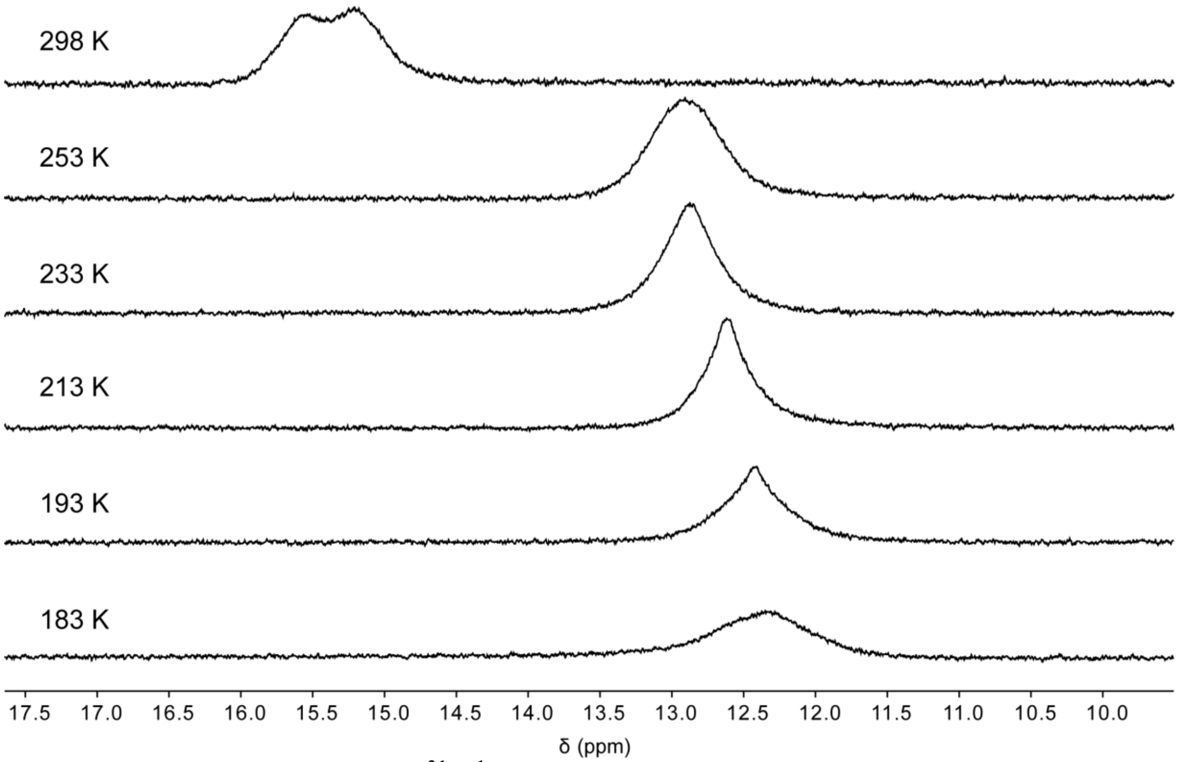

Figure s11. Variable-temperature $^{31}P\{^1H\}$ NMR spectra of *in,out*-**3c**·$2BH_3$ in CD_2Cl_2 (202 MHz).

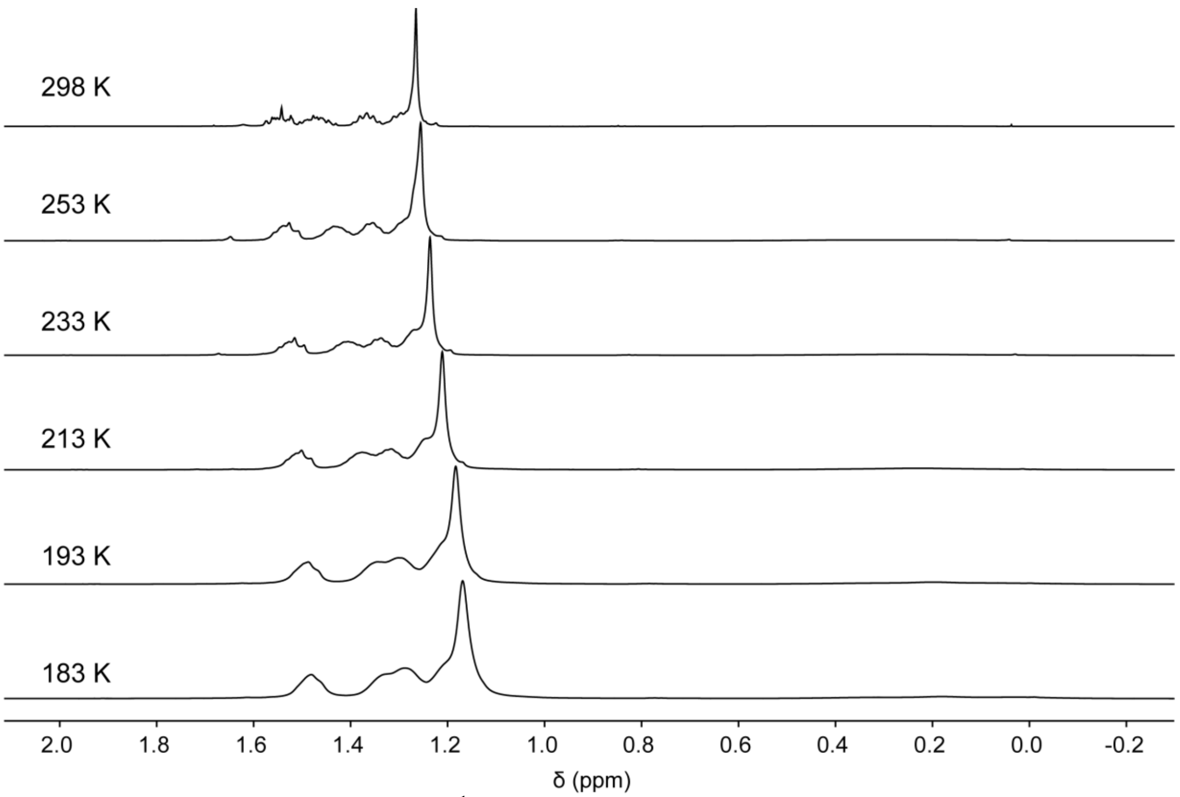

Figure s12. Variable-temperature ^{1}H NMR spectra of *in,out*-**3c**·$2BH_3$ in CD_2Cl_2 (500 MHz).

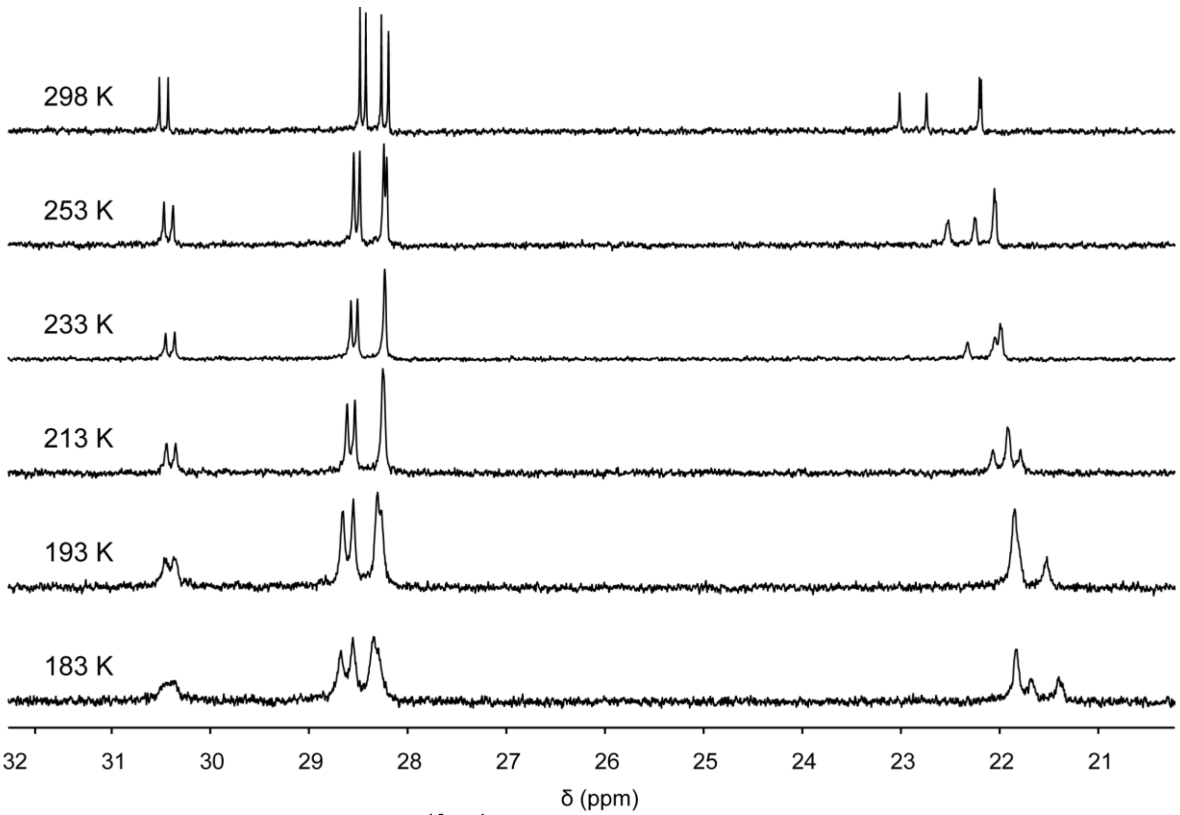

Figure s13. Variable-temperature $^{13}C\{^{1}H\}$ NMR spectra of *in,out*-**3c**·$2BH_3$ in CD_2Cl_2 (126 MHz).

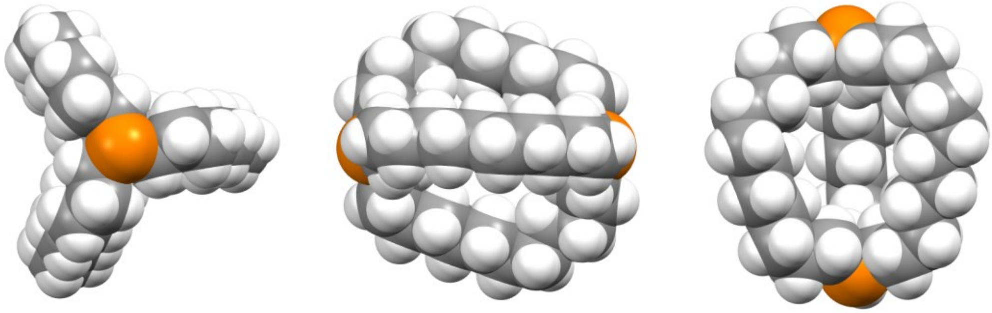

Figure s14. Space filling representations of *out,out*-**3b** comparable to those of *out,out*-**3c** in Figure 6.

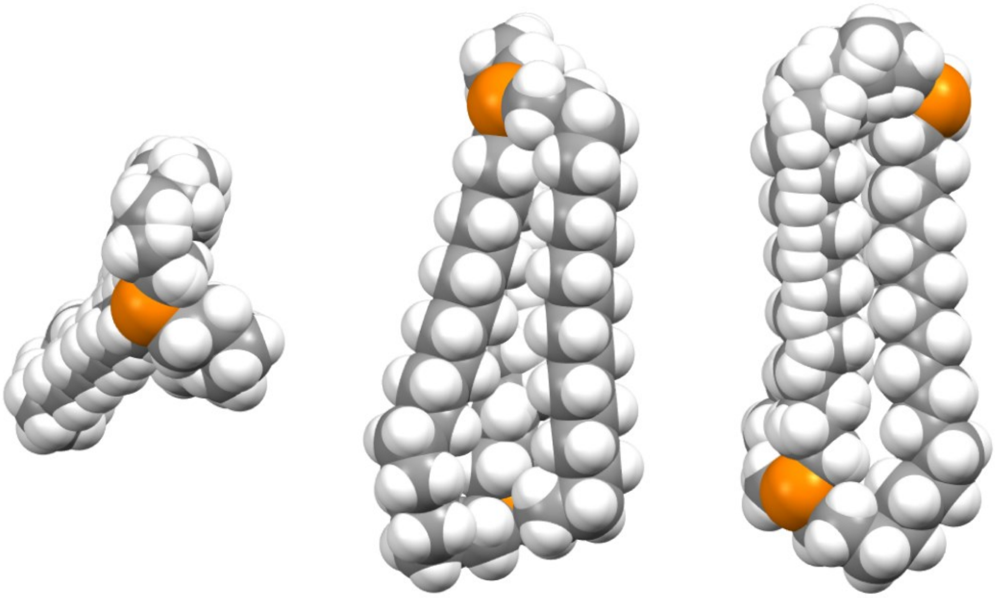

Figure s15. Space filling representations of *out,out*-**3e**.

Caption for video

The interconversion of **III**, **IV**, and **VI** in Scheme 2 is represented by models in which the EX (or P:) bridgeheads are depicted as triangles, initially with the blue sides "*in*" and the white sides "*out*". Threading one $(CH_2)_n$ chain through the macrocycle defined by the other two reverses these relationships (without pyramidal inversion or bond-breaking). This is termed "homeomorphic isomerization".

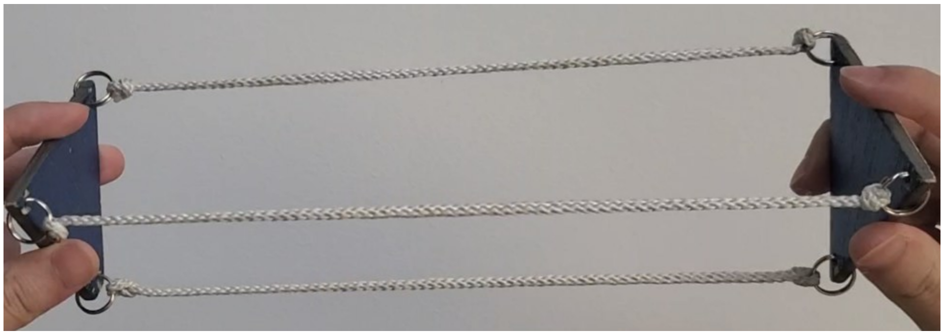

Table s1. Summary of crystallographic data.

	out,out-**3b**	*out,out*-**3c**	*out,out*-**3e**
empirical formula	$C_{36}H_{72}P_2$	$C_{42}H_{84}P_2$	$C_{54}H_{108}P_2$
formula weight	566.87	651.03	819.34
temperature [K]	110.0	110(2)	100.0
diffractometer	Bruker Venture	Bruker Gadds	Bruker Venture
wavelength [Å]	1.54178	1.54178	1.54178
crystal system	Triclinic	Rhombohedral	Monoclinic
space group	*P*-1	*R*-3c	C_12/c_1
unit cell dimensions:			
a[Å]	9.2062(4)	9.1903(19)	45.099(2)
b[Å]	9.3210(4)	9.1903(19)	12.6445(7)
c[Å]	25.5135(11)	89.58(2)	9.3990(5)
α[°]	85.783(2)	90	90
β[°]	82.881(2)	90	96.694(2)
γ[°]	60.9770(10)	120	90
V[$Å^3$]	1899.43(14)	6552(2)	5323.3(5)
Z	2	6	4
ρ_{calc}[Mg/m^3]	0.991	0.990	1.022
μ[mm^{-1}]	1.163	1.061	0.953
F(000)	636	2196	1848
crystal size [mm^3]	0.042 × 0.038 × 0.015	0.10 × 0.01 × 0.01	0.244 × 0.114 × 0.047
θ limit[°]	1.745 to 62.529	2.96 to 60.00	3.632 to 65.273
index range (h, k, l)	–10, 10, –10, 10, –29, 28	–10, 10, –10, 10, –99, 100	–52, 53, –14, 14, –10, 11
reflections collected	17631	14394	46796
independent reflections	6008	1047	4543
R(int)	0.0361	0.2152	0.0658
completeness to θ	98.9	95.5	99.4
max. and min. transmission	0.7522 and 0.6323	0.9895 and 0.9013	0.7479 and 0.6395
data/restraints/parameters	6008/0/343	1047/0/67	4543/1693/392
goodness-of-fit on F^2	1.046	1.005	1.089
R indices (final) [$I > 2\sigma(I)$]			
R_1	0.0414	0.0401	0.0604
wR_1	0.1038	0.0557	0.1447
R indices (all data)			
R_2	0.0449	0.0671	0.0741
wR_2	0.1063	0.0623	0.1557
largest diff. peak and hole [$eÅ^{-3}$]	0.454 and -0.170	0.257 and –0.250	0.387 and –0.300

Table s1. Summary of crystallographic data (continued).

	out,out-**3c**·$2BH_3$·$(C_5H_9CH_3)$	*out,out*-**3c**·$2BH_3$·$(C_6H_{11}CH_3)$	*out,out*-**3e**·$2BH_3$
empirical formula	$C_{48}H_{102}B_2P_2$	$C_{49}H_{104}B_2P_2$	$C_{54}H_{114}B_2P_2$
formula weight	762.86	776.88	847.01
temperature [K]	110(2)	273(2)	110.0
diffractometer	Bruker Gadds	Bruker Gadds	Bruker Venture
wavelength [Å]	1.54180	1.54178	1.54178
crystal system	Triclinic	Triclinic	Triclinic
space group	*P*-1	*P*-1	*P*-1
unit cell dimensions:			
a[Å]	10.3183(18)	10.312(2)	9.0197(5)
b[Å]	16.194(3)	16.214(3)	26.5241(5)
c[Å]	16.463(3)	16.671(3)	35.839(2)
α[°]	100.865(7)	101.424(10)	81.639(3)
β[°]	91.156(8)	91.088(11)	85.640(3)
γ[°]	106.588(7)	106.512(12)	87.030(3)
V[Å^3]	2581.2(7)	2611.1(10)	8451.2(8)
Z	2	2	6
ρ_{calc}[Mg/m^3]	0.982	0.988	0.999
μ[mm^{-1}]	0.947	0.943	0.907
F(000)	860	876	2868
crystal size [mm^3]	0.30 × 0.16 × 0.01	0.24 × 0.12 × 0.08	0.279 × 0.102 × 0.061
θ limit[°]	2.91 to 45.00	2.71 to 44.99	2.235 to 49.999
index range (*h*, *k*, *l*)	–7, 9, –14, 14, –15, 15	–9, 9, –14, 14, –15, 14	–8, 8, –26, 26, –35, 35
reflections collected	15936	14070	58948
independent reflections	4041	4131	16992
R(int)	0.1338	0.0774	0.0970
completeness to θ	96.8	97.8	98.1
max. and min. transmission	0.9906 and 0.7642	0.9283 and 0.8053	0.7315 and 0.6212
data/restraints/parameters	4041/941/556	4131/206/478	16992/21/1573
goodness-of-fit on F^2	1.059	1.064	1.254
R indices (final) [$I > 2\sigma(I)$]			
R_1	0.1058	0.1339	0.1249
wR_1	0.2672	0.3965	0.2338
R indices (all data)			
R_2	0.1844	0.1768	0.1582
wR_2	0.3107	0.4573	0.2478
largest diff. peak and hole [eÅ^{-3}]	0.392 and –0.313	0.713 and –0.423	0.384 and –0.508

Table s1. Crystallographic distances (Å) and angles (°) for *out,out*-**3b**.

	chain 1	chain 2	chain 3
P(1)···P(2)	10.8271(8)	–	–
P(1)···P(2) ···LP	173.52	–	–
P(2)···P(1) ···LP	172.89	–	–
P(1)-C(1/13/25)	1.8535(15)	1.8544(15)	1.8526(15)
P(2)-C(12/24/36)	1.8541(16)	1.8530(15)	1.8543(15)
C(1/13/25)-C(2/14/26)	1.528(2)	1.525(2)	1.528(2)
C(2/14/26)-C(3/15/27)	1.532(2)	1.530(2)	1.531(2)
C(3/15/27)-C(4/16/28)	1.524(2)	1.522(2)	1.521(2)
C(4/16/28)-C(5/17/29)	1.524(2)	1.520(2)	1.526(2)
C(5/17/29)-C(6/18/30)	1.530(2)	1.525(2)	1.530(2)
C(6/18/30)-C(7/19/31)	1.518(2)	1.517(2)	1.519(2)
C(7/19/31)-C(8/20/32)	1.525(2)	1.531(2)	1.524(2)
C(8/20/32)-C(9/21/33)	1.523(2)	1.526(2)	1.524(2)
C(9/21/33)-C(10/22/34)	1.523(2)	1.522(2)	1.519(2)
C(10/22/34)-C(11/23/35)	1.533(2)	1.529(2)	1.531(2)
C(11/23/35)-C(12/24/36)	1.525(2)	1.527(2)	1.526(2)
C(1/13/25)-P(1)-C(13/25/1)	98.67(7)	98.02(7)	98.68(7)
C(12/24/36)-P(2)-C(24/36/12)	99.21(7)	98.11(7)	97.65(7)
P(1)-C(1/13/25)-C(2/14/26)	114.7(1)	114.68(11)	114.16(10)
P(2)-C(12/24/36)-C(11/23/35)	115.55(11)	114.24(11)	114.28(11)
C(1/13/25)-C(2/14/26)-C(3/15/27)	113.68(12)	113.23(13)	113.01(12)
C(2/14/26)-C(3/15/27)-C(4/16/28)	114.93(13)	114.11(13)	114.70(12)
C(3/15/27)-C(4/16/28)-C(5/17/29)	112.77(13)	114.16(13)	113.17(12)
C(4/16/28)-C(5/17/29)-C(6/18/30)	115.06(13)	113.05(13)	114.66(12)
C(5/17/29)-C(6/18/30)-C(7/19/31)	114.22(13)	114.01(13)	114.35(13)
C(6/18/30)-C(7/19/31)-C(8/20/32)	114.22(13)	114.03(13)	114.64(13)
C(7/19/31)-C(8/20/32)-C(9/21/33)	112.50(13)	114.09(13)	111.51(13)
C(8/20/32)-C(9/21/33)-C(10/22/34)	114.69(13)	113.36(13)	114.99(13)
C(9/21/33)-C(10/22/34)-C(11/23/35)	113.90(13)	113.96(13)	113.42(13)
C(10/22/34)-C(11/23/35)-C(12/24/36)	113.22(13)	113.40(13)	112.75(13)

Table s2. Torsion angles (°) for *out,out*-**3b**.

	chain 1	chain 2	chain 3
C(13/25)-P(1)-C(1)-C(2)	–	176.24(11)	76.69(12)
C(1/25)-P(1)-C(13)-C(14)	80.58(12)	–	–179.31(11)
C(1/13)-P(1)-C(25)-C(26)	177.51(11)	77.41(12)	–
P(1)-C(1/13/25)-C(2/14/26)-C(3/15/27)	177.62(10)	–179.73(10)	–178.07(10)
C(1/13/25)-C(2/14/26)- C(3/15/27)-C(4/16/28)	66.78(18)	69.16(17)	70.81(17)
C(2/14/26)- C(3/15/27)-C(4/16/28)-C(5/17/29)	–179.44(13)	–178.65(13)	–178.23(13)
C(3/15/27)-C(4/16/28)-C(5/17/29)-C(6/18/30)	–179.05(13)	178.22(13)	–171.25(13)
C(4/16/28)-C(5/17/29)-C(6/18/30)-C(7/19/31)	–65.12(19)	–178.90(13)	–60.06(18)
C(5/17/29)-C(6/18/30)-C(7/19/31)-C(8/20/32)	–177.05(14)	–179.56(13)	178.28(13)
C(6/18/30)-C(7/19/31)-C(8/20/32)- C(9/21/33)	–172.15(14)	–59.38(19)	–179.55(13)
C(7/19/31)-C(8/20/32)- C(9/21/33)- C(10/22/34)	174.88(13)	–175.39(13)	–178.71(13)
C(8/20/32)- C(9/21/33)- C(10/22/34)-C(11/23/35)	–173.72(13)	–176.12(13)	–178.10(13)
C(9/21/33)- C(10/22/34)-C(11/23/35)-C(12/24/36)	68.20(17)	69.27(18)	70.67(17)
C(10/22/34)-C(11/23/35)-C(12/24/36)–P(2)	179.28(11)	–178.58(11)	–175.07(10)
C(11)-C(12)-P(2)-C(24/36)	–	75.18(13)	174.73(12)
C(23)-C(24)-P(2)-C(12/36)	173.76(11)	–	74.59(12)
C(35)-C(36)-P(2)-C(12/24)	78.60(12)	179.10(11)	–

Table s3. Crystallographic distances (Å), angles (°), and torsion angles (°) for *out,out*-**3c**.

P(1)···P(2)	12.948(3)	C(2)-C(3)-C(4)	114.44(14)
P(1)-C(1)	1.8361(15)	C(3)-C(4)-C(5)	114.03(13)
C(1)-C(2)	1.5197(19)	C(4)-C(5)-C(6)	113.52(13)
C(2)-C(3)	1.526(2)	C(5)-C(6)-C(7)	114.48(13)
C(3)-C(4)	1.524(2)	C(6)-C(7)-C(7)'	113.66(12)
C(4)-C(5)	1.510(2)	C(1)'-P(1)-C(1)-C(2)	77.09(15)
C(5)-C(6)	1.520(2)	C(1)"-P(1)-C(1)-C(2)	177.28(11)
C(6)-C(7)	1.519(2)	P(1)-C(1)-C(2)-C(3)	–179.20(11)
C(7)-C(7)"'	1.533(3)	C(1)-C(2)-C(3)-C(4)	69.54(18)
P(1)···P(2) ···LP	180.00	C(2)-C(3)-C(4)-C(5)	–177.81(13)
C(1)-P(1)-C(1)'	98.64(6)	C(3)-C(4)-C(5)-C(6)	–179.03(13)
C(1)-P(1)-C(1)"	98.64(6)	C(4)-C(5)-C(6)-C(7)	–177.58(14)
P(1)-C(1)-C(2)	115.63(11)	C(5)-C(6)-C(7)-C(7)"'	–178.65(16)
C(1)-C(2)-C(3)	114.09(12)	C(6)-C(7)-C(7)"'-C(6)"'	-63.1(2)

Table s4. Crystallographic distances (Å) and angles (°) for *out,out*-**3e** (dominant conformation).[a]

	chain 1[b]	chain 2	chain 3
P(1)···P(2)	17.978(1)	–	–
P(1)···P(2) ···LP	108.54	–	–
P(1)-C(1/19/37)	1.848(2)	1.832(2)	1.850(2)
P(2)-C(18/36/54)	1.848(2)	1.850(2)	1.832(2)
C(1/19/37)-C(2/20/38)	1.526(3)	1.560(3)	1.526(4)
C(2/20/38)-C(3/21/39)	1.523(3)	1.525(4)	1.508(5)
C(3/21/39)-C(4/22/40)	1.521(3)	1.506(4)	1.489(5)
C(4/22/40)-C(5/23/41)	1.523(3)	1.544(3)	1.525(4)
C(5/23/41)-C(6/24/42)	1.523(3)	1.504(4)	1.530(4)
C(6/24/42)-C(7/25/43)	1.524(3)	1.524(3)	1.526(4)
C(7/25/43)-C(8/26/44)	1.520(3)	1.522(5)	1.520(4)
C(8/26/44)-C(9/27/45)	1.520(3)	1.523(4)	1.524(4)
C(9/27/45)-C(10/28/46)	1.525(4)	1.524(4)	1.524(4)
C(10/28/46)-C(11/29/47)	1.520(3)	1.524(4)	1.523(4)
C(11/29/47)-C(12/30/48)	1.520(3)	1.520(4)	1.522(5)
C(12/30/48)-C(13/31/49)	1.524(3)	1.526(4)	1.524(3)
C(13/31/49)-C(14/32/50)	1.523(3)	1.530(4)	1.504(4)
C(14/32/50)-C(15/33/51)	1.523(3)	1.525(4)	1.544(3)
C(15/33/51)-C(16/34/52)	1.521(3)	1.489(5)	1.506(4)
C(16/34/52)-C(17/35/53)	1.523(3)	1.508(5)	1.525(4)
C(17/35/53)-C(18/36/54)	1.526(3)	1.526(4)	1.560(3)
C(1/19/37)-P(1)-C(19/37/1)	102.06(10)	100.30(11)	100.32(10)
C(18/36/54)-P(2)-C(36/54/18)	100.32(10)	100.30(11)	102.06(10)
P(1)-C(1/19/37)-C(2/20/38)	119.09(15)	107.63(17)	113.3(3)
P(2)-C(18/36/54)-C(17/35/53)	119.09(15)	113.3(3)	107.63(17)
C(1/19/37)-C(2/20/38)-C(3/21/39)	113.28(17)	114.6(2)	115.3(4)
C(2/20/38)-C(3/21/39)-C(4/22/40)	114.79(18)	115.0(2)	118.2(4)
C(3/21/39)-C(4/22/40)-C(5/23/41)	114.67(17)	113.8(3)	117.8(4)
C(4/22/40)-C(5/23/41)-C(6/24/42)	112.56(17)	112.5(2)	112.0(4)
C(5/23/41)-C(6/24/42)-C(7/25/43)	113.87(17)	115.2(3)	114.4(4)
C(6/24/42)-C(7/25/43)-C(8/26/44)	113.41(18)	115.5(3)	113.0(4)
C(7/25/43)-C(8/26/44)-C(9/27/45)	113.63(17)	112.2(4)	114.8(3)
C(8/26/44)-C(9/27/45)-C(10/28/46)	113.8(2)	115.6(3)	112.4(3)
C(9/27/45)-C(10/28/46)-C(11/29/47)	113.8(2)	112.4(3)	115.6(3)
C(10/28/46)-C(11/29/47)-C(12/30/48)	113.63(17)	114.8(3)	112.2(4)
C(11/29/47)-C(12/30/48)-C(13/31/49)	113.41(18)	113.0(4)	115.5(3)
C(12/30/48)-C(13/31/49)-C(14/32/50)	113.87(17)	114.4(4)	115.2(3)
C(13/31/49)-C(14/32/50)-C(15/33/51)	112.56(17)	112.0(4)	112.5(2)
C(14/32/50)-C(15/33/51)-C(16/34/52)	114.67(17)	117.8(4)	113.8(3)
C(15/33/51)-C(16/34/52)-C(17/35/53)	114.79(18)	118.2(4)	115.0(2)
C(16/34/52)-C(17/35/53)-C(18/36/54)	113.28(17)	115.3(4)	114.6(2)

[a]The atom labels C1 to C18, C19 to C36, and C36 to C54 have been changed from those in the CIF to correspond to those of *out,out*-**3e**·$2BH_3$. [b]This is the unique chain, the midpoint of which contains the C_2 symmetry axis.

Table s5. Torsion angles (°) for *out,out*-**3e** (dominant conformation).[a]

	chain 1[b]	chain 2	chain 3
C(19/37)-P(1)-C(1)-C(2)	–	-46.01(18)	56.99(18)
C(1/37)-P(1)-C(19)-C(20)	–177.95(16)	–	79.05(18)
C(1/19)-P(1)-C(37)-C(38)	69.1(3)	173.5(3)	–
P(1)-C(1/19/37)-C(2/20/38)-C(3/21/39)	–158.42(15)	–165.6(2)	172.9(5)
C(1/19/37)-C(2/20/38)-C(3/21/39)-C(4/22/40)	70.3(2)	62.9(3)	–168.4(6)
C(2/20/38)-C(3/21/39)-C(4/22/40)-C(5/23/41)	68.9(3)	55.9(4)	–169.9(6)
C(3/21/39)-C(4/22/40)-C(5/23/41)-C(6/24/42)	–176.8(2)	173.4(3)	–175.9(5)
C(4/22/40)-C(5/23/41)-C(6/24/42)-C(7/25/43)	–177.8(2)	180.0(3)	–179.9(4)
C(5/23/41)-C(6/24/42)-C(7/25/43)-C(8/26/44)	179.1(2)	–60.6(5)	–178.4(4)
C(6/24/42)-C(7/25/43)-C(8/26/44)-C(9/27/45)	–177.9(2)	–160.9(4)	–178.8(4)
C(7/25/43)-C(8/26/44)-C(9/27/45)-C(10/28/46)	178.14(16)	–175.9(4)	–178.6(4)
C(8/26/44)-C(9/27/45)-C(10/28/46)-C(11/29/47)	–174.1(2)	–175.9(4)	–174.0(4)
C(9/27/45)-C(10/28/46)-C(11/29/47)-C(12/30/48)	178.14(16)	–174.0(4)	–175.9(4)
C(10/28/46)-C(11/29/47)-C(12/30/48)-C(13/31/49)	–177.9(2)	–178.6(4)	–175.9(4)
C(11/29/47)-C(12/30/48)-C(13/31/49)-C(14/32/50)	179.1(2)	–178.8(4)	–160.9(4)
C(12/30/48)-C(13/31/49)-C(14/32/50)-C(15/33/51)	–177.8(2)	–178.4(4)	–60.6(5)
C(13/31/49)-C(14/32/50)-C(15/33/51)-C(16/34/52)	–176.8(2)	–179.9(4)	180.0(3)
C(14/32/50)-C(15/33/51)-C(16/34/52)-C(17/35/53)	68.9(3)	–175.9(5)	173.4(3)
C(15/33/51)-C(16/34/52)-C(17/35/53)-C(18/36/54)	70.3(2)	–169.9(6)	55.9(4)
C(16/34/52)-C(17/35/53)-C(18/36/54)–P(2)	–158.42(15)	–168.4(6)	62.9(3)
C(17)-C(18)-P(2)-C(36/54)	–	56.99(18)	–46.01(18)
C(35)-C(36)-P(2)-C(18/54)	69.1(3)	–	173.5(3)
C(53)-C(54)-P(2)-C(18/36)	–177.95(16)	79.05(18)	–

[a]The atom labels C1 to C18, C19 to C36, and C36 to C54 have been changed from those in the CIF to correspond to those of *out,out*-**3e**·$2BH_3$. [b]This is the unique chain, the midpoint of which contains the C_2 symmetry axis. The other chains are disordered and exchanged by the symmetry axis.

Table s6. Crystallographic distances (Å) and angles (°) for *out,out*-**3c**·$2BH_3 \cdot (C_5H_9CH_3)$ (dominant conformation).[a]

	chain 1	chain 2	chain 3
P(1)···P(2)	13.212(4)	–	–
P(1)···P(2)···B(2)	157.6(4)	–	–
P(2)···P(1)···B(1)	174.2(4)	–	–
P(1)-B(1)	1.906(12)	–	–
P(2)-B(2)	1.900(12)	–	–
P(1)-C(1/15/29)	1.812(8)	1.840(11)	1.87(3)
P(2)-C(14/28/42)	1.852(10)	1.708(13)	1.92(3)
C(1/15/29)-C(2/16/30)	1.480(7)	1.482(8)	1.480(8)
C(2/16/30)-C(3/17/31)	1.518(7)	1.517(8)	1.518(8)
C(3/17/31)-C(4/18/32)	1.504(8)	1.499(8)	1.502(9)
C(4/18/32)-C(5/19/33)	1.504(8)	1.499(8)	1.503(8)
C(5/19/33)-C(6/20/34)	1.509(8)	1.509(9)	1.504(9)
C(6/20/34)-C(7/21/35)	1.500(8)	1.496(8)	1.499(9)
C(7/21/35)-C(8/22/36)	1.505(8)	1.507(8)	1.504(9)
C(8/22/36)-C(9/23/37)	1.495(8)	1.493(9)	1.490(9)
C(9/23/37)-C(10/24/38)	1.520(8)	1.521(9)	1.522(9)
C(10/24/38)-C(11/25/39)	1.495(9)	1.488(9)	1.487(9)
C(11/25/39)-C(12/26/40)	1.508(8)	1.506(8)	1.505(9)
C(12/26/40)-C(13/27/41)	1.527(8)	1.529(9)	1.528(9)
C(13/27/41)-C(14/28/42)	1.525(9)	1.523(9)	1.524(10)
C(1/15/29)-P(1)-B(1)	114.8(5)	117.1(6)	109.2(15)
C(14/28/42)-P(2)-B(2)	112.9(6)	118.8(7)	109.7(10)
C(1/15/29)-P(1)-C(15/29/1)	105.9(4)	106(2)	102.0(16)
C(14/28/42)-P(2)-C(28/42/14)	101.7(5)	114.4(10)	97.1(11)
P(1)-C(1/15/29)-C(2/16/30)	116.0(5)	111.5(7)	128(3)
P(2)-C(14/28/42)-C(13/27/41)	116.0(7)	113.7(8)	110.0(18)
C(1/15/29)-C(2/16/30)-C(3/17/31)	115.4(6)	115.9(7)	115.0(8)
C(2/16/30)-C(3/17/31)-C(4/18/32)	115.2(6)	115.7(7)	115.2(8)
C(3/17/31)-C(4/18/32)-C(5/19/33)	115.5(6)	117.2(7)	115.9(9)
C(4/18/32)-C(5/19/33)-C(6/20/34)	116.6(6)	117.6(7)	116.9(8)
C(5/19/33)-C(6/20/34)-C(7/21/35)	116.4(6)	116.8(7)	117.7(8)
C(6/20/34)-C(7/21/35)-C(8/22/36)	116.2(7)	117.4(7)	115.4(8)
C(7/21/35)-C(8/22/36)-C(9/23/37)	116.0(6)	115.4(7)	117.6(9)
C(8/22/36)-C(9/23/37)-C(10/24/38)	114.8(7)	115.4(8)	114.0(9)
C(9/23/37)-C(10/24/38)-C(11/25/39)	115.4(7)	116.0(8)	116.7(9)
C(10/24/38)-C(11/25/39)-C(12/26/40)	114.3(7)	115.4(7)	115.3(8)
C(11/25/39)-C(12/26/40)-C(13/27/41)	115.0(6)	114.6(7)	114.7(8)
C(12/26/40)-C(13/27/41)-C(14/28/42)	111.9(6)	112.7(7)	112.2(8)

[a]The atom labels C1 to C14, C15 to C28, and C29 to C42 have been changed from those in the CIF to correspond to those of *out,out*-**3c**·$2BH_3 \cdot (C_6H_{11}CH_3)$.

Table s7. Crystallographic distances (Å) and angles (°) for *out,out*-**3c**·$2BH_3$·($C_6H_{11}CH_3$).

	chain 1	chain 2	chain 3
P(1)···P(2)	13.220(4)	–	–
P(1)···P(2) ···B(2)	158.4(4)	–	–
P(2)···P(1) ···B(1)	176.1(4)	–	–
P(1)-B(1)	1.864(12)	–	–
P(2)-B(2)	1.881(12)	–	–
P(1)-C(1/15/29)	1.823(8)	1.770(11)	1.929(12)
P(2)-C(14/28/42)	1.837(10)	1.738(12)	1.825(11)
C(1/15/29)-C(2/16/30)	1.509(11)	1.533(15)	1.430(15)
C(2/16/30)-C(3/17/31)	1.514(11)	1.563(15)	1.533(15)
C(3/17/31)-C(4/18/32)	1.516(12)	1.385(17)	1.434(17)
C(4/18/32)-C(5/19/33)	1.519(12)	1.441(16)	1.233(19)
C(5/19/33)-C(6/20/34)	1.502(13)	1.457(16)	1.418(19)
C(6/20/34)-C(7/21/35)	1.511(12)	1.468(15)	1.332(19)
C(7/21/35)-C(8/22/36)	1.530(13)	1.483(15)	1.466(18)
C(8/22/36)-C(9/23/37)	1.490(12)	1.460(15)	1.32(2)
C(9/23/37)-C(10/24/38)	1.528(13)	1.541(17)	1.43(2)
C(10/24/38)-C(11/25/39)	1.534(12)	1.382(17)	1.415(19)
C(11/25/39)-C(12/26/40)	1.529(13)	1.487(16)	1.481(18)
C(12/26/40)-C(13/27/41)	1.530(12)	1.602(15)	1.523(16)
C(13/27/41)-C(14/28/42)	1.499(13)	1.475(13)	1.400(16)
C(1/15/29)-P(1)-B(1)	115.9(5)	120.4(6)	103.7(5)
C(14/28/42)-P(2)-B(2)	112.8(6)	116.5(6)	110.7(6)
C(1/15/29)-P(1)-C(15/29/1)	105.7(5)	102.6(5)	107.0(5)
C(14/28/42)-P(2)-C(28/42/14)	105.0(5)	108.5(6)	102.3(5)
P(1)-C(1/15/29)-C(2/16/30)	114.2(6)	110.1(7)	118.7(9)
P(2)-C(14/28/42)-C(13/27/41)	116.4(7)	114.9(8)	122.2(10)
C(1/15/29)-C(2/16/30)-C(3/17/31)	114.1(7)	117.9(10)	112.8(10)
C(2/16/30)-C(3/17/31)-C(4/18/32)	115.4(7)	115.9(11)	114.4(13)
C(3/17/31)-C(4/18/32)-C(5/19/33)	114.6(8)	123.0(12)	129.5(16)
C(4/18/32)-C(5/19/33)-C(6/20/34)	116.3(8)	124.5(11)	131.9(17)
C(5/19/33)-C(6/20/34)-C(7/21/35)	114.6(7)	121.6(10)	128.7(18)
C(6/20/34)-C(7/21/35)-C(8/22/36)	115.4(8)	120.4(10)	128.4(15)
C(7/21/35)-C(8/22/36)-C(9/23/37)	115.2(8)	117.8(9)	127.5(17)
C(8/22/36)-C(9/23/37)-C(10/24/38)	113.8(8)	120.1(12)	116.5(18)
C(9/23/37)-C(10/24/38)-C(11/25/39)	114.1(8)	122.7(13)	105.0(17)
C(10/24/38)-C(11/25/39)-C(12/26/40)	112.7(8)	117.5(13)	115.2(15)
C(11/25/39)-C(12/26/40)-C(13/27/41)	114.3(8)	114.8(11)	116.6(12)
C(12/26/40)-C(13/27/41)-C(14/28/42)	112.6(8)	111.7(9)	115.7(12)

Table s8. Torsion angles (°) for *out,out*-**3c**·$2BH_3$·($C_5H_9CH_3$) (dominant conformation).[a]

	chain 1	chain 2	chain 3
C(15/29)-P(1)-C(1)-C(2)	–	–177.6(7)	–67(2)
C(1/29)-P(1)-C(15)-C(16)	–74.8(9)	–	177.2(15)
C(1/15)-P(1)-C(29)-C(30)	–58(5)	53(5)	–
P(1)-C(1/15/29)-C(2/16/30)-C(3/17/31)	179.7(6)	168.8(7)	–171(4)
C(1/15/29)-C(2/16/30)-C(3/17/31)-C(4/18/32)	–71.1(10)	–68.3(16)	–175(4)
C(2/16/30)-C(3/17/31)-C(4/18/32)-C(5/19/33)	–175.6(8)	167.6(12)	–67(3)
C(3/17/31)-C(4/18/32)-C(5/19/33)-C(6/20/34)	–179.5(8)	–179.3(13)	169.6(18)
C(4/18/32)-C(5/19/33)-C(6/20/34)-C(7/21/35)	61.9(12)	164.5(13)	173.0(19)
C(5/19/33)-C(6/20/34)-C(7/21/35)-C(8/22/36)	163.4(9)	–173.5(13)	–176.1(19)
C(6/20/34)-C(7/21/35)-C(8/22/36)-C(9/23/37)	–179.3(9)	174.1(13)	–155(2)
C(7/21/35)-C(8/22/36)-C(9/23/37)-C(10/24/38)	166.0(9)	179.1(13)	157(2)
C(8/22/36)-C(9/23/37)-C(10/24/38)-C(11/25/39)	178.9(9)	75.2(19)	160(2)
C(9/23/37)-C(10/24/38)-C(11/25/39)-C(12/26/40)	–69.6(12)	171.6(12)	62(3)
C(10/24/38)-C(11/25/39)-C(12/26/40)-C(13/27/41)	174.7(9)	–178.9(13)	55(3)
C(11/25/39)-C(12/26/40)-C(13/27/41)-C(14/28/42)	176.7(9)	–63.1(16)	74(3)
C(12/26/40)-C(13/27/41)-C(14/28/42)–P(2)	177.6(8)	–168.0(8)	167.5(16)
C(13)-C(14)-P(2)-C(28/42)	–	–56.9(10)	–173.6(12)
C(27)-C(28)-P(2)-C(14/42)	–167.2(9)	–	–63.8(16)
C(41)-C(42)-P(2)-C(14/28)	78(2)	–28(2)	–

[a]The atom labels C1 to C14, C15 to C28, and C29 to C42 have been changed from those in the CIF to correspond to those of *out,out*-**3c**·$2BH_3$·($C_6H_{11}CH_3$).

Table s9. Torsion angles (°) for *out,out*-**3c**·$2BH_3$·($C_6H_{11}CH_3$).

	chain 1	chain 2	chain 3
C(15/29)-P(1)-C(1)-C(2)	–	–173.7(7)	–64.9(8)
C(1/29)-P(1)-C(15)-C(16)	–71.4(10)	–	176.6(9)
C(1/15)-P(1)-C(29)-C(30)	–47.3(10)	63.7(10)	–
P(1)-C(1/15/29)-C(2/16/30)-C(3/17/31)	179.9(7)	173.4(8)	–173.7(7)
C(1/15/29)-C(2/16/30)-C(3/17/31)-C(4/18/32)	–71.1(10)	–80.2(18)	177.6(12)
C(2/16/30)-C(3/17/31)-C(4/18/32)-C(5/19/33)	–176.1(7)	163.3(15)	–99(2)
C(3/17/31)-C(4/18/32)-C(5/19/33)-C(6/20/34)	177.7(7)	–174.4(17)	173(2)
C(4/18/32)-C(5/19/33)-C(6/20/34)-C(7/21/35)	62.2(11)	167.4(15)	–167(2)
C(5/19/33)-C(6/20/34)-C(7/21/35)-C(8/22/36)	164.3(8)	–171.3(12)	163(2)
C(6/20/34)-C(7/21/35)-C(8/22/36)-C(9/23/37)	–178.7(8)	174.8(12)	–174(2)
C(7/21/35)-C(8/22/36)-C(9/23/37)-C(10/24/38)	164.4(9)	–175.8(11)	171.9(15)
C(8/22/36)-C(9/23/37)-C(10/24/38)-C(11/25/39)	–178.9(8)	71(2)	–150.6(18)
C(9/23/37)-C(10/24/38)-C(11/25/39)-C(12/26/40)	–73.3(11)	179.2(13)	161.3(14)
C(10/24/38)-C(11/25/39)-C(12/26/40)-C(13/27/41)	175.6(8)	–179.8(12)	–78(2)
C(11/25/39)-C(12/26/40)-C(13/27/41)-C(14/28/42)	–178.0(9)	–65.0(13)	177.8(15)
C(12/26/40)-C(13/27/41)-C(14/28/42)–P(2)	177.6(7)	–172.7(8)	–177.3(11)
C(13)-C(14)-P(2)-C(28/42)	–	–56.9(9)	–170.1(9)
C(27)-C(28)-P(2)-C(14/42)	–166.3(8)	–	–57.6(10)
C(41)-C(42)-P(2)-C(14/28)	49.4(15)	–61.3(15)	–

Table s10. Crystallographic distances (Å) and angles (°) for *out,out*-**3e**·$2BH_3$ (independent molecule 1 in Table 2).

	chain 1	chain 2	chain 3
P(1)···P(2)	19.407(3)	–	–
P(1)···P(2) ···B(2)	133.1(3)	–	–
P(2)···P(1) ···B(1)	149.2(3)	–	–
P(1)-B(1)	1.903(10)	–	–
P(2)-B(2)	1.903(9)	–	–
P(1)-C(1/19/37)	1.825(8)	1.827(8)	1.801(8)
P(2)-C(18/36/54)	1.825(8)	1.819(8)	1.831(8)
C(1/19/37)-C(2/20/38)	1.521(10)	1.543(11)	1.509(11)
C(2/20/38)-C(3/21/39)	1.541(11)	1.524(11)	1.523(11)
C(3/21/39)-C(4/22/40)	1.515(10)	1.517(11)	1.526(11)
C(4/22/40)-C(5/23/41)	1.517(11)	1.540(11)	1.536(11)
C(5/23/41)-C(6/24/42)	1.519(11)	1.519(11)	1.525(11)
C(6/24/42)-C(7/25/43)	1.517(11)	1.526(11)	1.512(11)
C(7/25/43)-C(8/26/44)	1.519(11)	1.505(11)	1.537(11)
C(8/26/44)-C(9/27/45)	1.511(11)	1.521(11)	1.523(11)
C(9/27/45)-C(10/28/46)	1.521(11)	1.525(11)	1.509(11)
C(10/28/46)-C(11/29/47)	1.503(11)	1.529(11)	1.503(11)
C(11/29/47)-C(12/30/48)	1.507(10)	1.521(11)	1.515(11)
C(12/30/48)-C(13/31/49)	1.509(11)	1.529(11)	1.517(11)
C(13/31/49)-C(14/32/50)	1.523(10)	1.522(11)	1.513(11)
C(14/32/50)-C(15/33/51)	1.502(11)	1.533(11)	1.528(11)
C(15/33/51)-C(16/34/52)	1.534(11)	1.518(11)	1.528(11)
C(16/34/52)-C(17/35/53)	1.529(11)	1.524(11)	1.524(11)
C(17/35/53)-C(18/36/54)	1.520(11)	1.515(11)	1.531(11)
C(1/19/37)-P(1)-B(1)	111.5(4)	112.7(5)	114.4(4)
C(18/36/54)-P(2)-B(2)	112.8(4)	113.2(4)	109.6(5)
C(1/19/37)-P(1)-C(19/37/1)	107.2(4)	103.9(4)	106.6(4)
C(18/36/54)-P(2)-C(36/54/18)	103.7(4)	109.3(4)	107.8(4)
P(1)-C(1/19/37)-C(2/20/38)	117.1(6)	116.6(6)	115.4(6)
P(2)-C(18/36/54)-C(17/35/53)	114.9(6)	118.3(6)	112.8(6)
C(1/19/37)-C(2/20/38)-C(3/21/39)	111.6(6)	112.1(7)	114.5(7)
C(2/20/38)-C(3/21/39)-C(4/22/40)	113.5(6)	112.7(6)	115.0(7)
C(3/21/39)-C(4/22/40)-C(5/23/41)	113.9(6)	114.2(7)	114.0(7)
C(4/22/40)-C(5/23/41)-C(6/24/42)	113.9(7)	114.6(7)	114.2(7)
C(5/23/41)-C(6/24/42)-C(7/25/43)	114.7(7)	113.1(7)	114.2(7)
C(6/24/42)-C(7/25/43)-C(8/26/44)	113.4(7)	113.2(7)	114.3(7)
C(7/25/43)-C(8/26/44)-C(9/27/45)	115.1(7)	115.1(7)	113.8(7)
C(8/26/44)-C(9/27/45)-C(10/28/46)	114.2(7)	113.7(7)	116.3(7)
C(9/27/45)-C(10/28/46)-C(11/29/47)	116.0(7)	113.2(7)	113.9(7)

C(10/28/46)-C(11/29/47)-C(12/30/48)	113.1(7)	113.7(7)	116.7(7)
C(11/29/47)-C(12/30/48)-C(13/31/49)	113.8(6)	112.4(7)	113.9(7)
C(12/30/48)-C(13/31/49)-C(14/32/50)	113.8(6)	114.5(7)	114.7(7)
C(13/31/49)-C(14/32/50)-C(15/33/51)	114.9(7)	112.4(7)	114.1(7)
C(14/32/50)-C(15/33/51)-C(16/34/52)	115.0(7)	113.4(7)	113.2(7)
C(15/33/51)-C(16/34/52)-C(17/35/53)	115.4(7)	114.3(7)	116.0(7)
C(16/34/52)-C(17/35/53)-C(18/36/54)	113.5(7)	114.5(7)	113.7(7)

Table s11. Torsion angles (°) for *out,out*-**3e**·$2BH_3$ (independent molecule 1 in Table 2).

	chain 1	chain 2	chain 3
C(19/37)-P(1)-C(1)-C(2)	–	66.3(7)	–44.5(7)
C(1/37)-P(1)-C(19)-C(20)	53.8(7)	–	166.3(6)
C(1/19)-P(1)-C(37)-C(38)	–65.8(6)	–178.8(6)	–
P(1)-C(1/19/37)-C(2/20/38)-C(3/21/39)	–170.8(5)	–97.9(8)	–176.5(5)
C(1/19/37)-C(2/20/38)-C(3/21/39)-C(4/22/40)	–174.9(7)	178.1(7)	–61.5(9)
C(2/20/38)-C(3/21/39)-C(4/22/40)-C(5/23/41)	175.9(7)	168.6(7)	–59.4(10)
C(3/21/39)-C(4/22/40)-C(5/23/41)-C(6/24/42)	–176.4(7)	55.4(9)	–179.1(7)
C(4/22/40)-C(5/23/41)-C(6/24/42)-C(7/25/43)	177.7(7)	176.3(7)	–164.1(7)
C(5/23/41)-C(6/24/42)-C(7/25/43)-C(8/26/44)	–178.1(7)	176.8(7)	–179.6(7)
C(6/24/42)-C(7/25/43)-C(8/26/44)-C(9/27/45)	179.1(7)	179.8(7)	–160.4(7)
C(7/25/43)-C(8/26/44)-C(9/27/45)-C(10/28/46)	179.6(7)	–177.7(7)	172.4(7)
C(8/26/44)-C(9/27/45)-C(10/28/46)-C(11/29/47)	–179.6(7)	–178.7(7)	–175.0(7)
C(9/27/45)-C(10/28/46)-C(11/29/47)-C(12/30/48)	179.3(7)	–178.2(7)	179.3(7)
C(10/28/46)-C(11/29/47)-C(12/30/48)-C(13/31/49)	–178.0(7)	–178.7(7)	–179.8(7)
C(11/29/47)-C(12/30/48)-C(13/31/49)-C(14/32/50)	177.4(7)	–176.0(7)	179.9(7)
C(12/30/48)-C(13/31/49)-C(14/32/50)-C(15/33/51)	–172.0(7)	–177.6(7)	177.9(7)
C(13/31/49)-C(14/32/50)-C(15/33/51)-C(16/34/52)	169.3(7)	–164.1(7)	–179.3(7)
C(14/32/50)-C(15/33/51)-C(16/34/52)-C(17/35/53)	–46.4(10)	–175.6(7)	–173.7(7)
C(15/33/51)-C(16/34/52)-C(17/35/53)-C(18/36/54)	-51.7(10)	68.3(9)	–66.5(10)
C(16/34/52)-C(17/35/53)-C(18/36/54)–P(2)	159.6(6)	65.9(9)	–164.6(6)
C(17)-C(18)-P(2)-C(36/54)	–	178.5(6)	–65.7(7)
C(35)-C(36)-P(2)-C(18/54)	32.3(7)	–	–82.5(7)
C(53)-C(54)-P(2)-C(18/36)	–178.6(6)	–66.5(7)	–

Table s12. Crystallographic distances (Å) and angles (°) for *out,out*-**3e**·$2BH_3$ (independent molecule 2 in Table 2).

	chain 1	chain 2	chain 3
P(1)···P(2)	19.800(3)	–	–
P(1)···P(2) ···B(2)	129.2(4)	–	–
P(2)···P(1) ···B(1)	153.0(3)	–	–
P(1)-B(1)	1.903(10)	–	–
P(2)-B(2)	1.920 (10)	–	–
P(1)-C(1/19/37)	1.827(8)	1.817(8)	1.814(8)
P(2)-C(18/36/54)	1.822(9)	1.779(10)	1.817(9)
C(1/19/37)-C(2/20/38)	1.520(11)	1.528(12)	1.523(11)
C(2/20/38)-C(3/21/39)	1.523(11)	1.490(13)	1.524(12)
C(3/21/39)-C(4/22/40)	1.513(11)	1.520(12)	1.521(12)
C(4/22/40)-C(5/23/41)	1.525(11)	1.524(11)	1.531(12)
C(5/23/41)-C(6/24/42)	1.515(11)	1.531(11)	1.511(11)
C(6/24/42)-C(7/25/43)	1.514(11)	1.530(11)	1.517(12)
C(7/25/43)-C(8/26/44)	1.507(11)	1.516(11)	1.527(11)
C(8/26/44)-C(9/27/45)	1.515(11)	1.517(11)	1.512(12)
C(9/27/45)-C(10/28/46)	1.517(11)	1.496(10)	1.539(12)
C(10/28/46)-C(11/29/47)	1.500(12)	1.498(11)	1.509(12)
C(11/29/47)-C(12/30/48)	1.509(12)	1.515(11)	1.534(12)
C(12/30/48)-C(13/31/49)	1.508(12)	1.503(11)	1.531(12)
C(13/31/49)-C(14/32/50)	1.508(12)	1.516(11)	1.526(12)
C(14/32/50)-C(15/33/51)	1.527(12)	1.507(11)	1.519(12)
C(15/33/51)-C(16/34/52)	1.472(13)	1.539(11)	1.544(13)
C(16/34/52)-C(17/35/53)	1.549(13)	1.504(11)	1.534(12)
C(17/35/53)-C(18/36/54)	1.499(12)	1.543(11)	1.534(13)
C(1/19/37)-P(1)-B(1)	112.2(4)	112.8(4)	115.1(4)
C(18/36/54)-P(2)-B(2)	106.4(5)	113.9(5)	112.2(5)
C(1/19/37)-P(1)-C(19/37/1)	105.8(4)	104.4(4)	105.7(4)
C(18/36/54)-P(2)-C(36/54/18)	105.8(5)	107.1(4)	111.3(5)
P(1)-C(1/19/37)-C(2/20/38)	116.0(6)	113.6(6)	113.6(6)
P(2)-C(18/36/54)-C(17/35/53)	122.1(7)	119.0(6)	110.8(6)
C(1/19/37)-C(2/20/38)-C(3/21/39)	113.6(6)	115.1(8)	114.7(7)
C(2/20/38)-C(3/21/39)-C(4/22/40)	113.5(7)	115.1(8)	114.2(7)
C(3/21/39)-C(4/22/40)-C(5/23/41)	114.3(7)	116.4(8)	114.3(8)
C(4/22/40)-C(5/23/41)-C(6/24/42)	114.1(7)	112.4(7)	113.7(7)
C(5/23/41)-C(6/24/42)-C(7/25/43)	114.2(7)	114.5(7)	115.2(7)
C(6/24/42)-C(7/25/43)-C(8/26/44)	115.6(7)	113.6(7)	113.4(7)
C(7/25/43)-C(8/26/44)-C(9/27/45)	114.5(7)	114.5(7)	114.8(7)
C(8/26/44)-C(9/27/45)-C(10/28/46)	116.2(7)	115.9(7)	113.3(7)
C(9/27/45)-C(10/28/46)-C(11/29/47)	114.4(7)	116.2(7)	113.6(7)

C(10/28/46)-C(11/29/47)-C(12/30/48)	115.9(7)	115.5(7)	112.1(8)
C(11/29/47)-C(12/30/48)-C(13/31/49)	115.0(8)	116.0(7)	114.2(8)
C(12/30/48)-C(13/31/49)-C(14/32/50)	115.2(8)	114.8(7)	110.8(8)
C(13/31/49)-C(14/32/50)-C(15/33/51)	113.3(8)	114.4(7)	114.5(8)
C(14/32/50)-C(15/33/51)-C(16/34/52)	113.9(9)	113.9(7)	110.1(8)
C(15/33/51)-C(16/34/52)-C(17/35/53)	113.3(8)	113.8(7)	115.6(8)
C(16/34/52)-C(17/35/53)-C(18/36/54)	113.6(8)	112.8(7)	113.7(8)

Table s13. Torsion angles (°) for *out,out*-**3e**·$2BH_3$ (independent molecule 2 in Table 2).

	chain 1	chain 2	chain 3
C(19/37)-P(1)-C(1)-C(2)	–	–46.7(7)	63.7(7)
C(1/37)-P(1)-C(19)-C(20)	–59.7(7)	–	–171.0(6)
C(1/19)-P(1)-C(37)-C(38)	62.8(7)	174.2(6)	–
P(1)-C(1/19/37)-C(2/20/38)-C(3/21/39)	–173.2(6)	170.5(6)	–174.4(6)
C(1/19/37)-C(2/20/38)-C(3/21/39)-C(4/22/40)	–169.1(7)	–65.4(11)	57.6(11)
C(2/20/38)-C(3/21/39)-C(4/22/40)-C(5/23/41)	–172.0(8)	–73.7(11)	60.8(10)
C(3/21/39)-C(4/22/40)-C(5/23/41)-C(6/24/42)	–171.6(8)	176.1(8)	–178.7(8)
C(4/22/40)-C(5/23/41)-C(6/24/42)-C(7/25/43)	–171.7(8)	170.9(7)	174.1(8)
C(5/23/41)-C(6/24/42)-C(7/25/43)-C(8/26/44)	–175.0(8)	175.9(7)	174.1(8)
C(6/24/42)-C(7/25/43)-C(8/26/44)-C(9/27/45)	–175.4(7)	175.8(7)	158.1(8)
C(7/25/43)-C(8/26/44)-C(9/27/45)-C(10/28/46)	–172.9(8)	176.6(7)	–175.3(8)
C(8/26/44)-C(9/27/45)-C(10/28/46)-C(11/29/47)	–175.1(8)	–179.0(7)	172.4(8)
C(9/27/45)-C(10/28/46)-C(11/29/47)-C(12/30/48)	–166.6(8)	179.0(7)	–178.0(9)
C(10/28/46)-C(11/29/47)-C(12/30/48)-C(13/31/49)	–172.4(8)	–176.0(7)	177.0(9)
C(11/29/47)-C(12/30/48)-C(13/31/49)-C(14/32/50)	–167.3(8)	–177.3(7)	–179.3(9)
C(12/30/48)-C(13/31/49)-C(14/32/50)-C(15/33/51)	–164.4(8)	–177.9(8)	–179.7(9)
C(13/31/49)-C(14/32/50)-C(15/33/51)-C(16/34/52)	75.5(11)	–172.3(7)	178.3(9)
C(14/32/50)-C(15/33/51)-C(16/34/52)-C(17/35/53)	78.5(10)	–67.9(10)	171.0(9)
C(15/33/51)-C(16/34/52)-C(17/35/53)-C(18/36/54)	–173.4(8)	176.9(7)	68.7(12)
C(16/34/52)-C(17/35/53)-C(18/36/54)–P(2)	91.8(9)	173.1(6)	176.1(6)
C(17)-C(18)-P(2)-C(36/54)	–	44.6(9)	–71.5(9)
C(35)-C(36)-P(2)-C(18/54)	-91.5(8)	–	27.3(9)
C(53)-C(54)-P(2)-C(18/36)	-173.9(6)	70.9(7)	–

Table s14. Crystallographic distances (Å) and angles (°) for *out,out*-**3e**·$2BH_3$ (independent molecule 3 in Table 2).

	chain 1	chain 2	chain 3
P(1)···P(2)	19.540(3)	–	–
P(1)···P(2) ···B(2)	133.3(4)	–	–
P(2)···P(1) ···B(1)	149.2(3)	–	–
P(1)-B(1)	1.907(10)	–	–
P(2)-B(2)	1.910(10)	–	–
P(1)-C(1/19/37)	1.805(8)	1.802(8)	1.814(9)
P(2)-C(18/36/54)	1.819(8)	1.830(8)	1.825(9)
C(1/19/37)-C(2/20/38)	1.529(10)	1.535(11)	1.536(12)
C(2/20/38)-C(3/21/39)	1.532(11)	1.528(11)	1.533(12)
C(3/21/39)-C(4/22/40)	1.510(11)	1.526(12)	1.501(12)
C(4/22/40)-C(5/23/41)	1.534(11)	1.518(12)	1.534(12)
C(5/23/41)-C(6/24/42)	1.515(11)	1.509(12)	1.535(12)
C(6/24/42)-C(7/25/43)	1.532(11)	1.510(12)	1.510(11)
C(7/25/43)-C(8/26/44)	1.518(11)	1.503(12)	1.535(12)
C(8/26/44)-C(9/27/45)	1.523(11)	1.505(13)	1.515(11)
C(9/27/45)-C(10/28/46)	1.521(11)	1.514(13)	1.519(11)
C(10/28/46)-C(11/29/47)	1.528(11)	1.499(13)	1.533(11)
C(11/29/47)-C(12/30/48)	1.513(11)	1.502(12)	1.511(12)
C(12/30/48)-C(13/31/49)	1.513(11)	1.500(13)	1.514(12)
C(13/31/49)-C(14/32/50)	1.520(10)	1.496(12)	1.512(11)
C(14/32/50)-C(15/33/51)	1.532(11)	1.511(12)	1.517(12)
C(15/33/51)-C(16/34/52)	1.539(11)	1.503(12)	1.522(11)
C(16/34/52)-C(17/35/53)	1.548(11)	1.544(12)	1.535(11)
C(17/35/53)-C(18/36/54)	1.517(11)	1.545(12)	1.534(11)
C(1/19/37)-P(1)-B(1)	110.7(4)	116.0(4)	111.1(4)
C(18/36/54)-P(2)-B(2)	112.8(5)	109.4(5)	114.7(4)
C(1/19/37)-P(1)-C(19/37/1)	106.9(4)	101.7(4)	110.0(4)
C(18/36/54)-P(2)-C(36/54/18)	108.1(4)	108.2(4)	103.3(4)
P(1)-C(1/19/37)-C(2/20/38)	118.2(6)	115.8(5)	115.3(6)
P(2)-C(18/36/54)-C(17/35/53)	114.8(6)	111.9(6)	118.2(6)
C(1/19/37)-C(2/20/38)-C(3/21/39)	113.8(6)	113.8(7)	110.0(7)
C(2/20/38)-C(3/21/39)-C(4/22/40)	113.1(6)	113.8(7)	113.0(7)
C(3/21/39)-C(4/22/40)-C(5/23/41)	115.0(7)	116.3(7)	111.9(7)
C(4/22/40)-C(5/23/41)-C(6/24/42)	112.9(7)	114.6(8)	113.3(7)
C(5/23/41)-C(6/24/42)-C(7/25/43)	115.1(7)	114.5(7)	113.4(7)
C(6/24/42)-C(7/25/43)-C(8/26/44)	114.0(7)	117.3(7)	114.3(7)
C(7/25/43)-C(8/26/44)-C(9/27/45)	113.7(7)	114.0(8)	114.0(7)
C(8/26/44)-C(9/27/45)-C(10/28/46)	114.6(7)	117.3(8)	113.8(7)
C(9/27/45)-C(10/28/46)-C(11/29/47)	114.1(7)	113.9(8)	112.4(7)

C(10/28/46)-C(11/29/47)-C(12/30/48)	113.8(7)	116.9(8)	114.7(7)
C(11/29/47)-C(12/30/48)-C(13/31/49)	115.4(7)	116.8(8)	113.5(7)
C(12/30/48)-C(13/31/49)-C(14/32/50)	114.0(7)	116.5(8)	115.9(7)
C(13/31/49)-C(14/32/50)-C(15/33/51)	113.9(7)	117.4(8)	112.9(7)
C(14/32/50)-C(15/33/51)-C(16/34/52)	115.1(7)	117.9(8)	114.4(7)
C(15/33/51)-C(16/34/52)-C(17/35/53)	113.8(7)	117.7(8)	114.4(7)
C(16/34/52)-C(17/35/53)-C(18/36/54)	114.1(7)	114.8(7)	115.8(7)

Table s15. Torsion angles (°) for *out,out*-**3e**·$2BH_3$ (independent molecule 3 in Table 2).

	chain 1	chain 2	chain 3
C(19/37)-P(1)-C(1)-C(2)	–	-39.4(7)	70.2(7)
C(1/37)-P(1)-C(19)-C(20)	-68.6(6)	–	176.1(6)
C(1/19)-P(1)-C(37)-C(38)	48.6(7)	161.7(6)	–
P(1)-C(1/19/37)-C(2/20/38)-C(3/21/39)	-172.1(5)	-174.6(6)	-98.5(8)
C(1/19/37)-C(2/20/38)-C(3/21/39)-C(4/22/40)	-173.5(7)	-54.9(10)	173.9(7)
C(2/20/38)-C(3/21/39)-C(4/22/40)-C(5/23/41)	177.4(7)	-56.7(10)	171.6(7)
C(3/21/39)-C(4/22/40)-C(5/23/41)-C(6/24/42)	-177.8(7)	177.0(7)	61.6(10)
C(4/22/40)-C(5/23/41)-C(6/24/42)-C(7/25/43)	176.7(7)	–167.6(8)	176.5(8)
C(5/23/41)-C(6/24/42)-C(7/25/43)-C(8/26/44)	178.9(7)	–177.7(8)	–176.6(7)
C(6/24/42)-C(7/25/43)-C(8/26/44)-C(9/27/45)	–179.8(7)	–170.3(9)	179.4(7)
C(7/25/43)-C(8/26/44)-C(9/27/45)-C(10/28/46)	177.2(7)	174.9(9)	–179.4(7)
C(8/26/44)-C(9/27/45)-C(10/28/46)-C(11/29/47)	–178.3(7)	–174.8(10)	178.7(7)
C(9/27/45)-C(10/28/46)-C(11/29/47)-C(12/30/48)	177.5(7)	–178.8(10)	–178.4(7)
C(10/28/46)-C(11/29/47)-C(12/30/48)-C(13/31/49)	–178.3(7)	179.5(10)	–179.2(8)
C(11/29/47)-C(12/30/48)-C(13/31/49)-C(14/32/50)	178.1(7)	–176.8(10)	–171.8(8)
C(12/30/48)-C(13/31/49)-C(14/32/50)-C(15/33/51)	–172.9(7)	–179.5(9)	–177.0(8)
C(13/31/49)-C(14/32/50)-C(15/33/51)-C(16/34/52)	171.7(7)	–177.3(9)	–169.1(8)
C(14/32/50)-C(15/33/51)-C(16/34/52)-C(17/35/53)	–47.4(10)	–171.0(8)	–179.4(7)
C(15/33/51)-C(16/34/52)-C(17/35/53)-C(18/36/54)	–53.7(10)	–64.3(11)	65.8(10)
C(16/34/52)-C(17/35/53)-C(18/36/54)–P(2)	159.3(6)	–170.0(6)	659.(9)
C(17)-C(18)-P(2)-C(36/54)	–	–65.8(7)	179.7(6)
C(35)-C(36)-P(2)-C(18/54)	–178.1(6)	–	–66.9(7)
C(53)-C(54)-P(2)-C(18/36)	31.0(7)	–83.4(7)	–
